# Two- and three-photon processes during photopolymerization in 3D laser printing†

**DOI:** 10.1039/d4sc03527e

**Published:** 2024-07-15

**Authors:** Anna Mauri, Pascal Kiefer, Philipp Neidinger, Tobias Messer, N. Maximilian Bojanowski, Liang Yang, Sarah Walden, Andreas-Neil Unterreiner, Christopher Barner-Kowollik, Martin Wegener, Wolfgang Wenzel, Mariana Kozlowska

**Affiliations:** a Institute of Nanotechnology (INT), Karlsruhe Institute of Technology (KIT) Kaiserstraße 12 76131 Karlsruhe Germany mariana.kozlowska@kit.edu; b Institute of Applied Physics (APH), Karlsruhe Institute of Technology (KIT) Kaiserstraße 12 76131 Karlsruhe Germany; c Institute of Physical Chemistry (IPC), Karlsruhe Institute of Technology (KIT) Kaiserstraße 12 76131 Karlsruhe Germany; d School of Chemistry and Physics, Centre for Materials Science, Queensland University of Technology (QUT) 2 George Street Brisbane QLD 4000 Australia

## Abstract

The performance of a photoinitiator is key to control efficiency and resolution in 3D laser nanoprinting. Upon light absorption, a cascade of competing photophysical processes leads to photochemical reactions toward radical formation that initiates free radical polymerization (FRP). Here, we investigate 7-diethylamino-3-thenoylcoumarin (DETC), belonging to an efficient and frequently used class of photoinitiators in 3D laser printing, and explain the molecular bases of FRP initiation upon DETC photoactivation. Depending on the presence of a co-initiator, DETC causes radical generation either upon two-photon- or three-photon excitation, but the mechanism for these processes is not well understood so far. Here, we show that the unique three-photon based radical formation by DETC, in the absence of a co-initiator, results from its excitation to highly excited triplet states. They allow a hydrogen-atom transfer reaction from the pentaerythritol triacrylate (PETA) monomer to DETC, enabling the formation of the reactive PETA alkyl radical, which initiates FRP. The formation of active DETC radicals is demonstrated to be less spontaneous. In contrast, photoinitiation in the presence of an onium salt co-initiator proceeds *via* intermolecular electron transfer after the photosensitization of the photoinitiator to the lowest triplet excited state. Our quantum mechanical calculations demonstrate photophysical processes upon the multiphoton activation of DETC and explain different reactions for the radical formation upon DETC photoactivation. This investigation for the first time describes possible pathways of FRP initiation in 3D laser nanoprinting and permits further rational design of efficient photoinitiators to increase the speed and sensitivity of 3D laser nanoprinting.

## Abstract 2

This study explores the mechanisms of photoactivation and radical formation during 3D laser printing using photoresists with a DETC photoinitiator. The role of DETC in high triplet states during radical polymerization is revealed.

## Graphical abstract

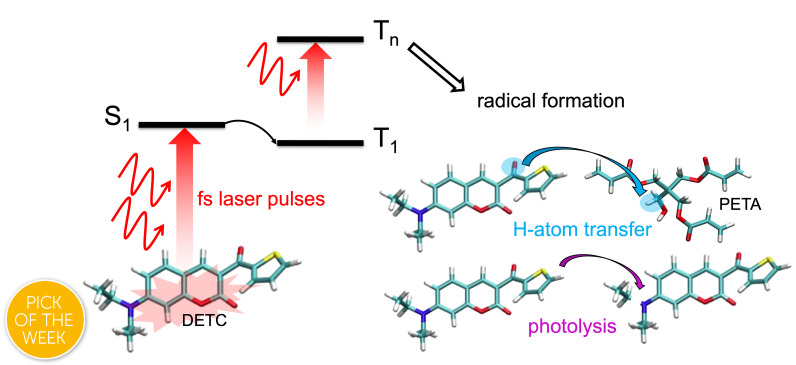

## Introduction

3D laser nanolithography is intensely investigated^1,2^ to enable manufacturing of devices with increasing speed and resolution.^2,3^ The strong three-dimensional spatial confinement of the exposed region in two-photon polymerization (TPP) enables resolutions that cannot be achieved using traditional one-step one-photon absorption (1PA) processes.^2,4,5^ Despite the tremendous progress in recent years,^6^ 3D laser nanoprinting using TPP still faces different limitations^7–10^ in resolution and speed that correlates strongly with the threshold laser power. This, in part, results from the limitations of the available photoinitiators (PIs) and resins: Kiefer *et al.*^11^ have reported a strong dependence of the printing sensitivity on the TPP initiation and, therefore, on the photochemical properties of the photoinitiator. Unfortunately, the photoinduced properties of common PIs cannot be deduced straightforwardly from their chemical composition and the electronic structure in the ground state or the lowest triplet state. Moreover, despite significant progress in 3D laser nanoprinting and the design of new two-photon PIs,^12–16^ a deep understanding of the photophysical and photochemical processes occurring upon multiphoton absorption is still scarce.^17,18^ Molecular-based structure–activity relationships for the dependencies observed experimentally, as well as the virtual design of new PIs towards higher 3D laser nanoprinting sensitivity should be improved.

**Fig. 1:**
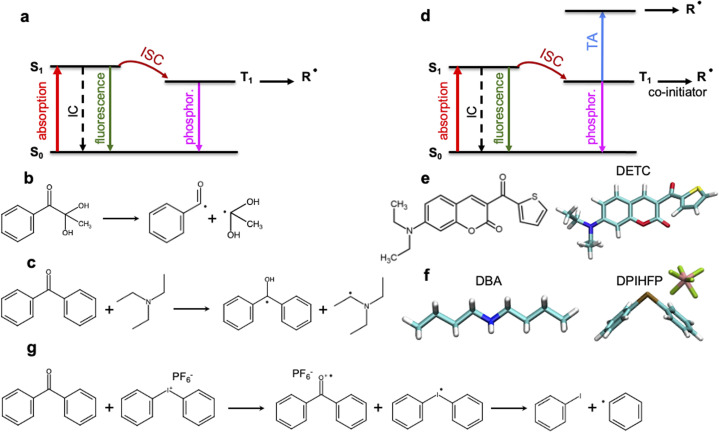
Schematic representation of the radical formation mechanisms in 3D laser nanoprinting. (a) Norrish type I PIs: photoinduced transition from the ground to the singlet excited state followed by intersystem crossing (ISC) to the lowest triplet state and the formation of radicals. (b) Radical formation *via* bond cleavage in Norrish type I photoinitiators. (c) Radical formation *via* hydrogen abstraction from a co-initiator in the presence of Norrish type II PIs. (d) Multiphoton activation of Norrish type II PIs with the formation of radicals either from T_1_ (in most cases based on electron transfer process) or from higher triplet states upon triplet state absorption (TA). In TPP, singlet–singlet absorption is often based on 2PA processes. (e) DETC photoinitiator. (f) Dibutylamine (DBA) and diphenyliodonium hexafluorophosphate (DPIHFP) co-initiators. (g) Radical generation during electron transfer from photoinitiator to DPIHFP onium salt. Note: besides phosphorescence, nonradiative decay from the lowest triplet state to the ground state can also occur (not shown in the figure).

Multiphoton photoinitiation involves complex photophysical processes between the excited states of the photoinitiator that goes beyond the conventional studies of the reactivity of photoactive molecules. It is commonly known that light irradiation often enables excitation of PI molecules from the ground state (S_0_) to the singlet excited state (S_1_), *e.g.* by two-photon absorption in TPP. From there, intersystem crossing to a triplet excited state (often T_1_) occurs (see Fig. 1a). This process can be followed by a cleavage reaction of the PI into two highly reactive radical fragments that start the polymerization.^4,5^ This photochemical cleavage, often assigned to Norrish type I PIs,^4,5,19^ is schematically depicted in Fig. 1b and is characterized by an α-scission of aldehydes and ketones (such as indole, pyrrole, (thio)phenol and aniline^19–21^) into two free radical intermediates. The photoinitiation process proceeds without α-scission in the case of PIs that behave as, so-called, Norrish type II PIs, such as benzophenone, thioxanthone, or ketocoumarins,^19–23^ where co-initiators are also added to the photoresist and two radicals are formed: one on the co-initiator and the other on the PI, respectively (see Fig. 1c). Depending on the nature of the triplet excited state of the Norrish type II PI (mostly T_1_) and a co-initiator molecule, there are different pathways towards radical formation: hydrogen abstraction, electron transfer or a combination of both, *e.g.* a hydrogen atom transfer (HAT) reaction,^19,24^ where carbon-centered radical intermediates^25^ are formed *via* photoexcited electron transfer followed by a proton transfer.^22,26^ HAT reactions were reported to occur with, *e.g.*, amines or amine synergists,^25^ which play the role of a hydrogen donor. Noticeable preference for an electron-based transfer reaction was observed in the case of electron-accepting co-initiators, *e.g.* onium salts (Fig. 1g).^19,27^

The performance of PIs used in 3D laser nanoprinting depends on the radical generation mechanism and the reactivity of the radical formed, as well as the effectiveness of the S_0_ → S_1_ transition, which controls the light absorbance characteristics of photoinitiator molecules. As depicted in Fig. 1, it is modulated by the intersystem crossing (ISC) between singlet and triplet states that depends on the spin–orbit coupling (SOC); it competes with internal conversion (IC) and radiative decay either from a singlet or a triplet state. Therefore, sufficiently fast ISC and long-living triplet states (in the case of Norrish type II PIs) are also required for the radical generation process. In addition, for two-photon based 3D printing of materials, high 2PA cross sections are important.^28^

Among the most sensitive photoresist systems that have successfully been applied in 3D laser nanoprinting, the combination of pentaerythritol triacrylate (PETA) monomer, that is cross-linking upon FRP, with 7-diethylamino-3-thenoylcoumarin (DETC) photoinitiator,^1,4,5,11,29,30^ is broadly used. DETC has shown high printing efficiency even in formulations with 0.25 and 0.50 wt% ^2,4,11,17,29^ of the initiator. Higher DETC concentrations are difficult to achieve due to limited solubility in PETA.^2,11^ It should be noted that other PIs, specifically Norrish type I, may show similar efficiency in 3D laser nanoprinting as DETC, but for much higher concentrations.^2,4,11^ Owing to its chemical structure (depicted in Fig. 1e), DETC behaves as Norrish type II PI^23^ and, for photoresists with co-initiators, shows *N* = 2 photon absorption dependence (using femtosecond lasers with around 800 nm).^4^ It was also reported to efficiently initiate polymerization in 3D laser nanoprinting without any co-initiator.^4,29^ In such a case, absorption of three photons of the same energy was required.^29^ Similar *N* = 3 photon absorption dependence of DETC was reported in other 3D printing studies, which suggested the singlet–singlet transition by 2PA and triplet–triplet transition by 1PA (*i.e.* triplet absorption, TA) to higher triplets^4,31^ (schematically depicted in Fig. 1d). *N*-photon absorption in both cases was estimated using the “reciprocity law” of the laser power at the polymerization threshold with exposure time.^29^ Presently, there is a lack of understanding of the different *N*-photon absorption of DETC and the radical formation mechanism with the use of this and similar PIs. This limits the further optimization of initiators for rapid 3D laser nanoprinting.

Here, we report quantum mechanical (QM) calculations of DETC in the ground and excited states to fully elucidate the relevant photophysical processes and photochemical reactions of DETC in the presence and absence of co-initiators such as dibutylamine (DBA) and diphenyliodonium hexafluorophosphate (DPIHFP). On this basis, we reveal the radical formation mechanisms upon the multiphoton activation of this PI in 3D laser nanoprinting. In combination with experimental data (3D laser nanoprinting), we analyze the two-photon and three-photon processes in DETC photosensitization and identify pathways for an efficient radical generation. Among the hypothetical radical polymerization mechanisms considered, we explain in detail the intermolecular HAT of DETC with DBA and PETA, as well as demonstrate the impact of the photoinduced electron transfer of DETC in the presence of DPIHFP. We investigate the formation of alkyl radicals by PETA or DBA *via* the reaction of monomers or co-initiators with DETC in higher triplet states. Moreover, we reveal the alkyl radicals formed as the source of the most reactive radical species necessary for FRP of PETA in 3D laser nanoprinting with DETC. We believe that our contribution is important in rationalizing experimental observations that have been poorly understood for many years and in comprehending the designing principles for new PIs for faster 3D printing. Given the limitations and challenges of available QM methods, we attempted to demonstrate possible pathways of radical generation and polymerization initiation.

## Computational details

### Geometry optimization and rates calculation

All calculations were performed using Density Functional Theory (DFT) and Time-Dependent DFT (TD-DFT) methods. Ground state geometry optimization of DETC was performed using temperature annealing approach utilizing the CAM-B3LYP^32^ functional with def2-TZVP^33^ basis set and Grimme's D3-dispersion correction with Becke–Johnson damping, *i.e.* D3(BJ).^34^ Global minimum structures in the ground state were used for excited state calculations (singlet and triplet states), including spectra generation and optimization of excited states using TD-CAM-B3LYP-D3(BJ)/def2-TZVP. T_2_ and T_5_ excited states could not be properly optimized and therefore were not reported in the paper. Geometry optimization was performed using Gaussian16 Rev. C.01 ^35^ with default ultra-fine grid for numerical integrations and energy convergence criterion of 10^−8^ Hartree. Calculations were performed both in the gas phase and acetonitrile (ACN) solvent using polarizable-continuum-model (PCM)^36^ implicit solvent model, *i.e.*, for excited states non-equilibrium solvation method for all single point calculations and equilibrium PCM procedure for excited states geometry optimizations. All optimized geometries obtained were confirmed with vibrational analysis and, in the case of excited states, confirmed also *via* molecular orbitals analysis. The computational scheme used was optimized considering available experimental data and calculations of 0–0 transition energy *ν*_00_ ^37,38^ (*i.e.*, between vibrational states of the ground and the first singlet excited electronic states), as described in Section 2 in ESI.† The quality of the TD-DFT setup for vertical excitation energies of DETC was confirmed by the GW (one-body Green's function with the dynamically screened Coulomb interaction) approximation and Bethe–Salpeter equation (BSE)^39,40^ implemented in TURBOMOLE v7.4 ^41^ (see Tables S2 and S22†). Analysis of the charge (electron and hole) differences between the ground and excited states were performed using Multiwfn (version 3.6) analyzer.^42^

Spin-unrestricted DFT scheme (U)CAM-B3LYP-D3(BJ) was used for the optimization of radicals and biradicals considered in this study. All thermodynamic quantities were computed after vibrational analysis using implicit ACN (PCM model) using (U)CAM-B3LYP-D3(BJ)/def2-TZVP level of theory in Gaussian16. The reaction temperature of 298 K and pressure of 1 atm were considered. Rates for IC, ISC and Reversible Intersystem Crossing (RISC) were calculated employing the Franck–Condon approximation (FC)^43^ (see details in ESI†) using MOMAP 2022A (2.3.3)^44–49^ (Molecular Materials Property Prediction Package). TD formalism was used. The radiative rate constants, *i.e.*, fluorescence and phosphorescence, were calculated using DETC optimized singlet and triplet state structures at ACN and in the gas phase with MOMAP. Adiabatic Hessian (AH) potential energy surfaces (PES) model within TD-DFT was used for the computation of all the radiative rates.

### Absorption spectra calculation

1PA,^50^ 2PA^51,52^ and three-photon absorption (3PA)^52,53^ spectra were computed both in the gas phase and implicit ACN and PETA (PCM model) using TD-CAM-B3LYP-D3(BJ)/def2-TZVP in Dalton 2020.0 ^54^ and Gaussian16 Rev. C.01 (only 1PA). 2PA and 3PA spectra were calculated considering a single laser beam, linearly polarized light with parallel polarization (as experimental set-up); transition moments were defined on two- and three photons of the same frequency, respectively. Spectra were plotted using half width at half maximum (HWHM) of 0.1 eV and Lorentzian type broadening function. TA spectra were computed with TD-(U)CAM-B3LYP-D3(BJ)/def2-TZVP approach in ACN starting from the T_1_ optimized geometry, obtained *via* TD-DFT using Gaussian16. To evaluate the intensities of transitions between two vibronic states, the Franck–Condon principle was employed considering that the nuclear positions are mostly unaltered by the electron jump which takes place during the electronic transition.

Vibrationally resolved singlet and triplet spectra were computed at TD-DFT level of theory within the Franck–Condon^55,56^ (FC) approximation at 100 K with Adiabatic Hessian (AH-G16) as PES model. Spectra were plotted with HWHM of 80 cm^−1^ (*i.e.*, 0.01 eV), convergence factor of 1.0 × 10^−4^ a.u. and Lorentzian type broadening function. The energy transitions from vibrational levels of the initial state to the vibrational levels of the final state, *i.e.*, higher triplet excited state, were considered. The strength of a particular transition was obtained as a result of the population of that state and the overlap between initial and final vibrational wavefunctions for that transition. Spectra were computed using Dynavib^57,58^ in implicit ACN with TD-CAM-B3LYP-D3(BJ)/def2-TZVP level of theory, starting from the lowest triplet excited state and higher triplet excited states. Due to significant geometry differences between T_1_ and T_3_ states in ACN (see Fig. S11†), which go beyond the harmonic vibrational potentials within FC approximation, the corresponding spectrum could not be generated. Several different models, employed for the computation of the PES, *i.e.* time-independent and -dependent FC and non-Condon simulation including Duschinsky effect^59^ and FC and non-Condon simulations based on linear coupling model (LCM) model,^60,61^ have not improved the output.

### Experiments

To investigate the polymerization laser threshold of various DETC-containing resists, the previously reported setup^11^ was applied. Herein, a mode-locked femtosecond laser oscillator (Mai Tai HP, Spectra-Physics), working at *R*_p_ = 80 MHz repetition rate and *λ* = 820 nm center wavelength, a microscope lens (Leica HCX PL APO 100x/1.4-0.7 Oil CS) for beam focussing in oil-immersion mode, and an acousto-optic modulator (AA MT80-A1.5-IR) were utilized. The resists were prepared by adding 7-diethylamino-3-thenoylcoumarin (DETC; 30 μmol), dibutylamine (DBA; 7.5 μmol, 15 μmol, 30 μmol, 60 μmol, and 120 μmol), or diphenyliodonium hexafluorophosphate (DPIHFP, 15.6 μmol, 31.2 μmol, and 62.4 μmol), respectively, to pentaerythritol triacrylate (PETA; 2 g) in a vial under constant stirring. DETC was purchased from Exciton. DPIHFP (>97.5% purity) was purchased from TCI. PETA (technical grade) and DBA (99.5% purity) were purchased from Sigma-Aldrich.

To measure the laser polymerization threshold for a given exposure time *t*_exp_, separated points of the photoresist were illuminated with different laser powers *P*. The lowest laser power, which still forms a visible polymer dot^29^ after development with acetone is then defined as the laser polymerization threshold power *P*_th_.

## Results and discussion

### One-photon and multiphoton absorption

**Fig. 2:**
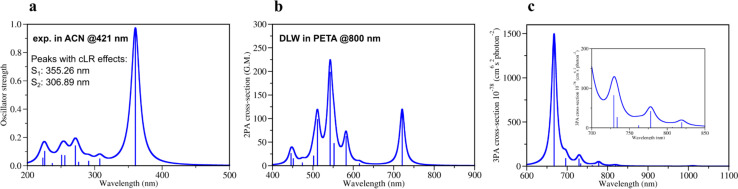
Theoretically calculated spectra of DETC using TD-CAM-B3LYP/def2-TZVP: (a) one-photon absorption spectrum and (b) two-photon absorption spectrum in acetonitrile (PCM implicit solvent model) (c) three-photon absorption spectrum in the gas phase with the insertion of the zoomed spectrum in the region 700–850 nm. All spectra were obtained starting from the optimized ground state geometry of DETC using CAM-B3LYP-D3(BJ)/def2-TZVP level of theory. Spectra were plotted using half width at half maximum (HWHM) of 0.1 eV and Lorentzian type broadening function. Vibrationally-resolved 1PA spectrum is depicted in Fig. S2.† Note: DLW denotes direct laser writing.

Quantum mechanical calculations of photochemical processes and photoreactivity of molecules are either computationally very expensive or not accurate enough, *i.e. via* the use of multireference based post-Hartree–Fock methods or DFT formalism, respectively. To calibrate the accuracy of the computational protocol a set of DFT and GW approximation Bethe–Salpeter equation (GW-BSE)^39,40^ calculations were performed and compared to available experimental data (Sections 1, 2 and 7 in ESI†) as well as several wavefunction-based methods (Section 5 in ESI†). Considering reported 1PA and emission data of DETC in PETA^4^ and in acetonitrile (ACN) (see Fig. S3†), we have calculated 1PA spectra using linear-response TD-DFT with long-range corrected CAM-B3LYP^32^ to describe the one-photon process (Fig. 2a, S2, and Table S1†). The calculated 1PA spectrum of DETC in ACN, as well as the excitation energies computed with GW-BSE approach and the values of *E*_0–0_ energies (*ν*_00_ ^37,38^*i.e.*, between the ground vibrational states of the two electronic states considered) show good agreement with the experiment. The 1PA spectrum of DETC (visualized in Fig. 2a) indicates the blue-shifted absorption maximum (by ∼60 nm) in comparison with the measured spectrum of DETC in ACN (359.30 nm *vs.* 421 nm). However, the consideration of vibronic effects modulates the red-shift in spectra, being closer to the experiment (see Fig. S2 and S3†). The computation of absorption and emission energies has also been performed while including corrected linear response (cLR)^62^ and state specific (SS) corrections for the solvation model, *i.e.*, external iteration (EI).^63,64^ This data is reported in Tables S6–S8.† The value of *E*_0–0_ is 439.04 nm (2.82 eV) with cLR instead of 407.44 nm (3.04 eV) with LR, indicating an impact of state-specific formulations in TD-DFT. The experimental Stokes shift of DETC in ACN is 460.50 nm (2.69 eV, see Table S3†), which could be well reproduced using an optimized computational protocol. The calculated emission characteristics of DETC are consistent with the reported literature data as well, *i.e.*, the fluorescence rate in ACN is 4.30 × 10^8^ s^−1^ (Table S20†), while the experimentally reported values in toluene and methanol (22) are ∼2–4 × 10^9^ s^−1^.

For the estimation of the character of vertical excitations of DETC, the transition orbitals and the analysis of its charge transfer (CT) characteristics^65^ have been performed. They are depicted and described in detail in Section 3 in ESI,† where the comparison with the spin-component scaled coupled cluster (SCS-CC2) method is provided. From Table S12,† it is clearly seen that CT in DETC is rather strong: transitions S_0_ → S_1_, S_0_ → T_1_ and S_0_ → T_7_ exhibit less negative *t* parameters, suggesting higher CT, while the remaining transitions have highly negative *t* values, indicating a mixed character of CT and local exciton (LE) transitions, as depicted in Fig. S6.† The change of the electronic structure of DETC following light-induced transitions, the molecular orbitals (MOs) and the electron density delocalization over its π-conjugated system are depicted in Fig. S4–S7† and explained in detail in Section 3 in ESI.†

The quality of calculated 1PA spectra directly impacts the accuracy of 2PA spectra necessary to evaluate the efficiency of PI in TPP. Even if 1PA and 2PA involve strictly defined transitions between ground and excited states of a molecule, the underlying light-induced processes are different in nature and follow different selection rules, *e.g.*, in terms of symmetry of the state and the electric dipole transitions. Therefore, not all molecules are both 1PA and 2PA active, however, the calculated 2PA spectrum of DETC (Fig. 2b) reveals its 2PA activity. The 2PA spectrum of DETC in ACN shows maximum absorption at 720.84 nm with the highest calculated cross-section at this wavelength of 119.0 GM (Fig. 2b, and Table S13†). The spectrum possesses a blue-shift in comparison to experiments^16,66^ which is a repercussion of the blue-shift in the 1PA spectrum (Fig. 2a), where the calculated and measured absorption maximum are located at 359.30 nm and 421.00 nm, respectively (see Table S1 and Fig. S3†). The calculated 2PA cross-section is higher than the recently reported 2PA cross-sections of DETC in dichloromethane (82 GM)^16^ and dimethyl sulfoxide (40 GM)^66^ at 780 nm and 800 nm, respectively. The calculated 2PA using the implicit representation of these solvents is reported in Fig. S8.† Considering spectral shifts in the 1PA spectra, they still demonstrate good correlations.^2–5,30,31^ Here, we note that the direct comparison of 2PA cross-sections between experiment and theory is challenging due to the high sensitivity of cross-section values to the variety of experimental parameters and referencing methods, as well as the selection of parameters for the calculation of theoretical spectra^51^ and consideration of environmental, *i.e.*, solvent, effects. Moreover, experimentally reported 2PA cross-sections of DETC differ between 82 GM and 40 GM, depending on the solvent and measurement technique (two-photon induced fluorescence and z-scan, respectively).^16,66^ Several factors contribute to the discrepancy in these values. Firstly, the determination of 2PA absorption cross-sections using two-photon fluorescence has an accuracy limited to 8–10%.^67^ Secondly, it is known that z-scan data typically underestimates 2PA cross-section by up to a factor of 2.5–5,^68^ bringing the true value measured *via* z-scan closer to 100–200 GM. Finally, it is important to keep in mind that neither of the mentioned experimental studies of DETC considered the potential for higher-order absorption processes.

To evaluate the possibility for DETC to simultaneously absorb three photons during singlet–singlet 3PA, the respective spectrum in the gas phase was calculated (Fig. 2c, and Table S14†). A peak related to S_0_ → S_1_ transition was found to be at 1010.74 nm with a 3PA cross-section of 5.6 × 10^−78^ cm^6^ s^2^ photon^−1^. The cross-section values related to transitions between S_0_ and higher excited states using the laser wavelength of 819–778 nm (*i.e.*, S_0_ → S_3_, S_0_ → S_4_ and S_0_ → S_5_) are in the range of 1–42 × 10^−78^ cm^6^ s^2^ photon^−1^. Therefore, theoretical calculations indicate that DETC might be prone to instantaneously absorb three photons, leading to very high singlet excited states. Since such highly excited states tend to relax rapidly *via* conical intersections (typical vibrational frequencies of modes coupled to the electronic transition have been calculated to be on the order 20 THz, *i.e.*, 50 fs),^69^ which are unstable, they will decay to the lowest singlet excited state. Since the S_1_ state is typically reached *via* 2PA using femtosecond lasers, no additional photons (*i.e.*, more than 2) would be detected in the nonlinearity fits of the threshold laser power *versus* exposure time in the TPP (discussed further). Due to the same reason, 3PA processes may not be observed at all using fluorescence as the detection system, since two- and three-photon induced fluorescence quantum yields can significantly vary due to channel branching in the excited state manifold (singlet and/or triplet in nature) of dye molecules. It has been shown for fluorene derivatives that shifting the excitation wavelength from the UV to the near-infrared region significantly increases the contribution of three-order processes compared to first and second order.^70^ 3PA processes were not considered in the fits to the z-scan data of DETC.^66^ Therefore, accurate excitation wavelength z-scan data would be needed to simultaneously analyze contributions both from two- and three order processes.^71^ To the best of our knowledge, no such analysis has been performed on DETC, likely due to experimental challenges such as contributions from self-phase modulation, impulsive stimulated Raman scattering, wavelength-dependence of higher-order processes *etc.*

### Multiphoton 3D laser nanoprinting using DETC

**Fig. 3:**
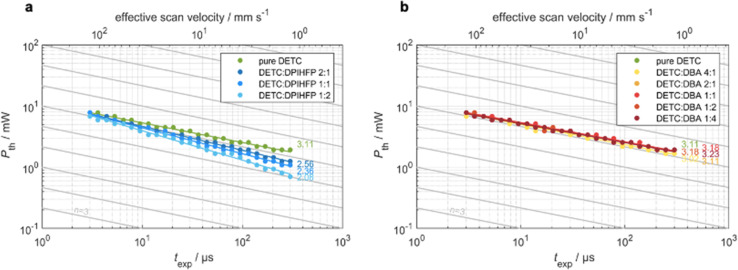
Measured threshold laser power *versus* exposure time on a double-logarithmic scale for resist systems with only DETC as initiator as well as resist systems with DETC and additionally (a) diphenyliodonium hexafluorophosphate (DPIHFP) and (b) dibutylamine (DBA) co-initiators in varying ratios. The slopes of the fits indicate the nonlinearity of the resist system. The gray lines are guides to the eye with a slope of *N* = 3 as expected for the DETC system. (a) With increasing concentration of DPIHFP, the nonlinearity changes from *N* = 3 to *N* = 2. (b) Independent on the concentration of DBA, the nonlinearity stays at *N* = 3.

To analyze the various aspects of DETC performance in 3D laser nanoprinting, we investigate different photoresist compositions, *i.e.*, with pure DETC as the photoinitiator and after the addition of the co-initiators: DBA or DPIHFP. Both co-initiators were considered since they were demonstrated to decrease the threshold laser power for rapid 3D laser nanoprinting.^11^ Here, DBA was found to be more efficient than triethylamine, which is a more typical amine-based co-initiator than DBA. Moreover, no lowering in the degree of co-sensitization due to volatility, side reactions or concentration-induced absorption changes, reported for other co-initiators based on tertiary amines, have been noticed.^72^ Point exposure experiments were performed using PETA as a monomer to evaluate the initiation mechanism of these photoresist compositions. In Fig. 3, we observe significant differences in the experimental threshold laser power *vs.* exposure time, *i.e.*, different scaling behaviors, for all resist systems. Here, the *N* ∼ 2 photon dependence^11^ is solely observed for resists comprising DETC and DPIHFP (Fig. 3a). As previously reported, pure DETC shows *N* ∼ 3 scaling.^29^ No changes in scaling after the addition of the DBA as a co-initiator are observed (Fig. 3b).

The underlying mechanisms for radical generation in all these cases may proceed *via* photoinduced HAT, electron transfer or TA. With onium salts, DETC is most likely to participate in electron transfer (ET) and/or energy transfer from the photoexcited sensitizer to the iodonium cation, followed by the rapid decomposition of the resulting unstable diaryliodine radical that prevents back electron transfer and renders the overall process irreversible.^27,58^ HAT was reported for DETC (and similar ketocoumarins) with amine-based co-initiators.^4,73,74^ Since ET happens on the picosecond timescale,^75^ it is faster than HAT, which was shown to be a diffusion modulated process.^76^ This agrees with the observation from 3D printing of PETA: the electron transfer process between DETC and DPIHFP results in the 2PA-initiated polymerization. The HAT reaction between DBA and DETC, involving a two-step intermolecular transfer (see Fig. 1c), should not occur from the T_1_ excited state of DETC, because the nonlinearity does not decrease from *N* = 3 to 2, as observed for onium salt. As mentioned above, 3PA of DETC has not been reported up to date, however such a process may well occur, in addition to 2PA, as was reported for other chromophores.^77^ This may be the reason why the nonlinearity dependence is slightly higher than *N* = 3. Such 3PA with 775–800 nm may excite DETC to S_3_–S_5_ states. However, due to fast decay to the S_1_ excited state, higher singlet states of DETC do not participate in the radical formation. Therefore, TA, resulting in highly reactive DETC triplet states, seems to be more probable to occur. This should allow the polymerization reaction, while the radical reaction channel from the T_1_ state of DETC is hindered due to diffusion-controlled reactions, particularly in high-viscosity resins.^76^

We have compared the reactivity of DETC (in the T_1_ state) in HAT reaction with DBA in terms of reaction energy profile and transition state that leads to the radical formation according to Fig. 1c. Obtained energies and reaction mechanisms are depicted in Fig. S19 and Table S25.† HAT reaction from co-initiator to DETC is not spontaneous from S_0_ state of DETC since the Gibbs free energy is 48.19 kcal mol^−1^ in ACN. Upon photoactivation of DETC and the formation of the T_1_ state, the Gibbs free energy lowers to 0.44 kcal mol^−1^ and the energy barrier (from the transition state calculation) is 6.72 kcal mol^−1^. Thus, it allows HAT with the rate of 7.33 × 10^7^ s^−1^, calculated employing the Eyring equation^78^ at room temperature and pressure, in ACN. Therefore, HAT between DBA and DETC in the T_1_ state should theoretically occur, resulting in the *N* ∼ 2 in 3D printing, however, it is not observed in printing conditions. Recently, Xue *et al.*^79^ have reported that the reaction of benzylidene ketone PIs with the amine-based co-initiators is predominantly controlled by material diffusion rather than by an activation process. We believe this may be the reason why resists with DBA do not show two-photon scaling during 3D printing of PETA using DETC, reported in Fig. 3b. The QM calculations of HAT reaction between DETC and DBA are presented in Section 8 in ESI.†

Consequently, bimolecular radical initiation shows a dependence on the exposure time and the concentration of the co-initiator (Fig. 3a). For longer exposure times or higher concentrations of DPIHFP, the bimolecular mechanism dominates over TA, however, it lowers the sensitivity of printing.

### Photophysical processes of DETC upon singlet–singlet excitation

**Fig. 4:**
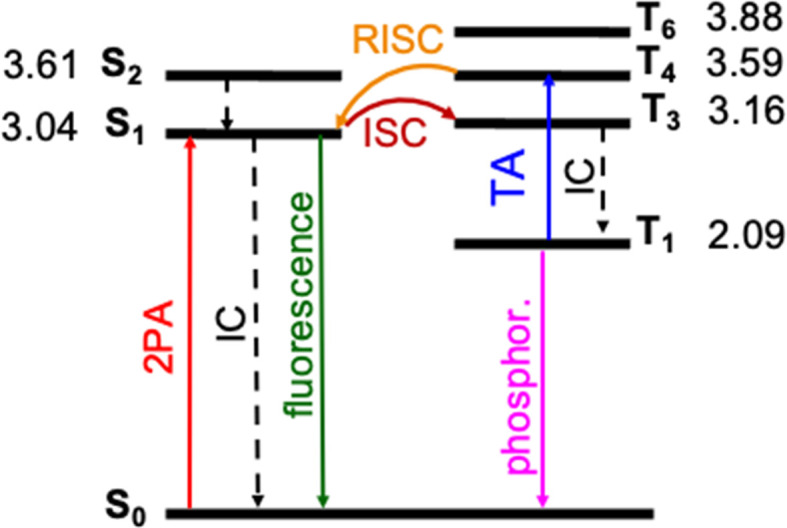
Jablonski diagram of DETC. Excited states were optimized in acetonitrile (PCM model) using TD-CAM-B3LYP-D3(BJ)/def2-TZVP level of theory. Adiabatic energy values, including zero-point-energy (ZPE) correction, are reported in eV. Rates of transitions between different states, calculated using electronic structure parameters, obtained with TD-DFT approach, are listed in Table 1. Note: besides phosphorescence, nonradiative decay from the lowest triplet state to the ground state can also occur (not shown in the figure).

The photosensitization of PIs can be activated in T_1_ triplet state (see Fig. 1), which, in principle, is achieved when SOC, thus, the ISC from singlet to triplet excited states is high enough. The direct SOC between the S_1_ state of DETC and the T_1_ state is 0.97 cm^−1^ and happens with an ISC rate of 8.84 × 10^4^ s^−1^ (see Table 1). This observation suggests that the ISC pathways *via* conical intersections and other triplet states would impact the photoactivation of DETC. Indeed, after geometry optimization (see description in ESI†), analysis of electronic properties and calculation of SOC and ISC rates involving two singlet states (S_1_ and S_2_) and several triplet (T_1_, T_3_, T_4_, T_6_, while T_2_ and T_5_ did not converge) states of DETC, we can conclude that direct ISC between S_1_ → T_1_ should not be the main pathway for the formation of the T_1_ state. From the Jablonski diagram of DETC in ACN, depicted in Fig. 4, it can be stated that upon absorption from the ground state, S_1_ excited state couples strongly to the T_3_ excited state with the SOC of 7.55 cm^−1^ (see Table 1), resulting in a fast and efficient ISC rate of ∼1.72 × 10^8^ s^−1^. Here, we have to note several important observations. Firstly, the T_3_ state, optimized in ACN, shows a twist of −97.7° of the thiophenyl group of DETC with respect to the core (dihedral angle C9–C8–C11–C17, see Fig. S1, S10, and S11†). Such a perpendicular-like position, as previously noted by Tozer *et al.*^80^ is unnatural for the DETC molecule and contrasts with the dihedral angle in the ground state geometry of −45.9°. It is not present in the case of T_3_ computed in the gas phase, where the twist is only by −41.45°. Such strong structural differences were not observed between the ground state and the S_1_ geometries optimized in ACN and gas phase (see Fig. S11†). Secondly, we encountered the poor convergence of the correlation function in the computation of ISC rates between T_3_ and S_1_, which were both optimized in implicit ACN (see explanation in Section 6 in ESI, Table S19a and Fig. S16†). This suggests an influence of the polarization included by the implicit solvent and subtle (complex) electronic nature of higher excited states of DETC that could not be resolved with the LR-TD-DFT. Higher level methods and the consideration of nonequilibrium conditions might be necessary, however it is beyond the present investigations. The ISC rate successfully converged utilizing the T_3_ state optimized in the gas phase (see Fig. S16†), therefore it was used for the computation of photophysical rates of DETC (Table 1).

Nonradiative and radiative rates. Internal conversion (IC), intersystem crossing (ISC) and reverse ISC (RISC) of DETC in implicit ACN and gas phase calculated with TD-CAM-B3LYP-D3(BJ)/def2-TZVP level of theory. IC, ISC and RISC rates (in s^−1^) were calculated using nonadiabatic coupling matrix elements (NACME, in au), spin–orbit-coupling (SOC, in cm^−1^), considering non-equilibrium solvent effects, and reorganization energy (*λ*, in eV) using approach described in methods and explained in ESI (Section 6). Radiative rates (fluorescence and phosphorescence) of DETC in implicit ACN were calculated with the same level of theory using Δ*E* (in eV) and lifetime (in s). NA refers to “not available” and in the case of the rate signifies that the correlation function was not converged. All rates are calculated considering equilibrium states

**Table:** 

Nonradiative rate		Rate (s^−1^)	NACME (au)	*λ* (eV)
IC	S_1_ → S_0_	1.60 × 10^8^	0.009	0.205
	S_2_ → S_0_	NA	0.004	1.341
	T_6_ → T_1_	1.25 × 10^10^	0.006	0.171
	T_4_ → T_1_	4.50 × 10^10^	0.006	0.478
	aT_3_ → T_1_	1.76 × 10^11^	0.003	0.572

a T_3_ was optimized in the gas phase, while S_1_, S_2_ and T_1_ in ACN. The energy gap considered was computed between T_3_ and S_1_, S_2_ or T_1_ in ACN and SOC in ACN.

**Table:** 

Nonradiative rate		Rate (s^−1^)	SOC (cm^−1^)	*λ* (eV)
ISC	S_1_ → T_1_	8.84 × 10^4^	0.97	0.027
	aS_1_ → T_3_	1.72 × 10^8^	7.55	0.316
	S_1_ → T_4_	3.94 × 10^6^	4.49	0.201
	S_1_ → T_6_	6.56 × 10^6^	9.51	0.336
	S_2_ → T_1_	6.33 × 10^8^	22.96	0.474
	aS_2_ → T_3_	1.57 × 10^11^	26.96	0.390
	S_2_ → T_4_	7.94 × 10^10^	25.81	0.429
	S_2_ → T_6_	5.75 × 10^6^	9.43	0.960
	T_1_ → S_0_	8.64 × 10^4^	2.16	0.181
RISC	T_1_ → S_1_	5.18 × 10^4^	0.80	0.021
	aT_3_ → S_1_	5.39 × 10^9^	5.47	0.284
	T_4_ → S_1_	2.44 × 10^9^	7.68	0.600
	T_6_ → S_1_	1.38 × 10^8^	2.09	0.362
	T_1_ → S_2_	3.59 × 10^6^	15.52	0.351
	aT_3_ → S_2_	5.34 × 10^6^	6.41	0.384
	T_4_ → S_2_	6.89 × 10^6^	4.30	1.974
	T_6_ → S_2_	3.52 × 10^8^	2.35	0.712

**Table:** 

Radiative rate	Rate (s^−1^)	Δ*E* (eV)	Lifetime (s)
Fluorescence	4.30 × 10^8^	3.04	2.33 × 10^−9^
Phosphorescence	7.69 × 10^−1^	2.09	1.30

The energy difference between the S_1_ and T_3_ states in ACN is −0.12 eV, as depicted in Fig. 4, *i.e.*, the adiabatic T_3_ state lies slightly higher than the S_1_ state. According to the calculation of the vertical energy differences by several wavefunction-based methods using the optimized structures from TD-DFT (see Tables S16 and S17† with the respective explanation), the T_2_ state in ACN is closer to the S_1_ state, but both T_2_ and T_3_ are still higher than S_1_. However, all post-HF-based energies obtained represent only the differences in the vertical energies, while TD-DFT data are for the optimized states. Unfortunately, the T_2_ state of DETC could not be reliably optimized in TD-DFT in both ACN and the gas phase to evaluate the competitiveness of the ISC *via* S_1_ → T_2_ transition. We assume that this fact may be connected to the limitations of the LR-TD-DFT for this specific state, *i.e.*, the transition electron density of T_2_ (as well as T_5_, which could not be obtained) is differently captured in TD-DFT than in SCS-CC2 in ACN (see Fig. S7†). Electron density differences for other states of DETC are more consistent within TD-DFT and SCS-CC2, therefore, we rely on them.

The ISC between S_1_ → T_3_ shows dominant ππ* → nπ* character (60% in the gas phase), supporting El-Sayed rule^81–83^ for ISC processes. It also indicates vibronic spin–orbit interactions that increase the SOC.^84^ Since SOC is impacted by the way the solvent effects are included (see Table S18†), for all calculations we use the SOC with the included non-equilibrium effects for the solvent, resulting in data that correlates better with experiments (Table S19†). Even if such SOC value is lower than in the gas phase, the ISC rate, considering the Δ*E*(S_1_–T_3_) of −0.12 eV is still reasonable (1.72 × 10^8^ s^−1^), competing with IC (1.60 × 10^8^ s^−1^) and fluorescence (4.30 × 10^8^ s^−1^). We have to point out that the parameters described and the shape of molecular orbitals during the transitions are strongly dependent on molecular vibrations, vibronic couplings and solvation effects, resulting in the mixing of nπ* and ππ* states, which is observed for S_1_ → T_3_. Therefore, more detailed analysis of transition dynamics and subtle differences should be accessed by a higher level of theory and non-adiabatic molecular dynamics to obtain more realistic values. This was not in the scope of the current investigation.

Once DETC reaches T_3_ state, it undergoes fast IC decay to the T_1_ state with the rate of 1.76 × 10^11^ s^−1^ (Table 1). The lifetime of 1.16 × 10^−5^ s (11.6 μs) for the non-radiative decay of T_1_ (*i.e.*, ISC T_1_–S_0_) allows other processes to occur. Charge density analysis, *i.e.*, the localization of electron-donating (hole) and electron-accepting (electron) parts of T_1_ excited state of DETC demonstrates the activation of carbonyl moiety (atoms C11–O12, see areas in green in Fig. S6†) with higher local electron densities that should participate in electron transfer and HAT. Therefore, DETC should be prone to attack the amine-based co-initiator permitting H-atom transfer reaction. However, as discussed above, it is hindered in the present study. This is shown by the deviation of nonlinearities far away from 2 that is expected for the typical Norrish type II PIs after PI activation to the T_1_ state.

### Multiphoton activation of DETC to higher triplet states

DETC initiates FRP in the absence of any co-initiator molecule with the use of three photons at ∼800 nm^2,4,11,29,30^ (see Fig. 3). It is an interesting observation since DETC ketyl radicals alone are less active: the bond dissociation energy of O–H bond in ketyl radicals is relatively low (*e.g.* 16 kcal mol^−1^ in acetone^85^). This indicates a different pathway for PI photoreactivity in TA, shifting the electronic state of DETC to higher triplet states. To define which triplet excited state can be reached by the upcoming third photon during 3D printing, a one-photon based triplet–triplet excitations in ACN were calculated (listed in Tables S21, and S22†).

**Fig. 5:**
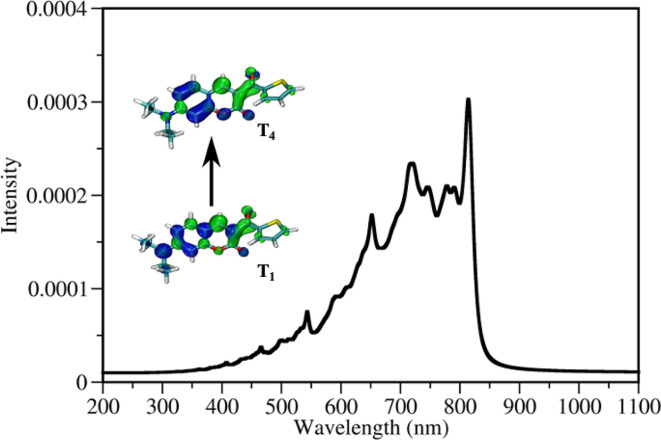
Vibrationally resolved triplet–triplet spectrum of DETC for its T_1_ → T_4_ transition employing T_1_ as starting geometry. Visualization of the electron density difference in T_1_ and T_4_ excited states compared to the electronic structure of DETC in the ground state is reported in the inset. Electron donating regions, *i.e.* holes, and electron accepting, *i.e.* electrons, are marked in blue and green, respectively. An isovalue of 0.002 a.u. was used for visualization.

The presence of a peak located at 815 nm (see Table S21†), *i.e.* value close to the fs laser wavelength used in experiment, confirms the TA absorption of photons in the T_1_ state. However, such a treatment neglects the influence of vibronic coupling of the electronic transition, providing a simplified view of absorption spectra. To overcome this limitation and to assign the transition to the activated triplet state, vibrationally resolved triplet spectra^55^ for several transitions between T_1_ → T_*n*_ (*n* = 4, 6) were computed (see Fig. S17†). Among spectra obtained, the transition T_1_ → T_4_ overlaps with the experimentally used photon energy (see Fig. 5). Therefore, the excitation of DETC in 3D printing should allow the transition of the PI most probably to the T_4_ state with the charge density difference depicted on the upper left panel in Fig. 5. Interestingly, the oscillator strength of TA at the printing wavelength is much higher for DETC than for other Norrish type II PIs.^18,31^ Fast TA may also be the reason why the HAT reaction with DBA (from T_1_ of DETC) is hindered, indicating a high probability of the DETC photoreactivity from higher triplet states. Note that in such states DETC may also decay to the lowest triplet state (calculated IC_T_4_−T_1__ is 4.50 × 10^10^ s^−1^, see Table 1) or return to singlet states *via* reverse intersystem crossing (RISC). RISC rates of DETC from the T_3_–T_4_ states (*e.g.* rate of 5.39 × 10^9^ s^−1^ for T_3_ → S_1_, 2.44 × 10^9^ s^−1^ for T_4_ → S_1_), might compete with IC permitting deactivation of DETC, *i.e.* its depletion, which was also reported to occur using the second inhibition laser source of 800 nm (femtosecond)^4^ and 808 nm (continuous).^24^

### Mechanisms of radical formation

To further estimate the radical formation of DETC in high triplet states, initiating FRP, several mechanisms were considered. Similarly, to the formation of aminoalkyl radicals of DBA (see Fig. 1c) photoactivated H-abstraction reaction^85^ was calculated for DETC (see Section 9.1). Higher C–H activation possibility with lower bond dissociation enthalpy (BDE) is triggered by PI in high triplets (BDE lower than 8.8 kcal mol^−1^ in comparison to 82 kcal mol^−1^ in S_0_, see Table S26†). However, the process is not spontaneous (see the Gibbs free energy values in Table S27†) and could not even compete with spontaneous internal conversion (Table 1), disabling the radical formation *via* C–H bond breaking, depicted in Fig. S21a.† This is also the reason for the hindered formation of the hypothetically highly reactive DETC biradicals that were suggested to occur for similar chromophore molecules^43,86–88^ (see Section 9.3 in ESI†). Indeed, if such biradicals are formed, they should initiate FRP from an excited state of DETC (higher than T_3_) spontaneously (see Section 10 in ESI†). However, neither such intramolecular HAT was confirmed experimentally, nor the calculated Gibbs free energies *vs.* nonradiative decay should permit it. Unfortunately, transition state calculations using higher triplet states of DETC could not be obtained to further predict the kinetics of this reaction. Interestingly, the formation of DETC radicals *via* photolysis should in theory be spontaneous (Section 9.2). The cleavage of N14–C15, C15–C16 or C8–C11 bonds of DETC (see Fig. S21c†) is thermodynamically more favorable in T_4_ (Table S29†) with the Gibbs free energy even −26.96 kcal mol^−1^ in the case of N14–C15 bond cleavage. However, the entropic contribution to the Gibbs free energy, calculated using the vibrational frequencies within harmonic approximation and considering implicit ACN, is relatively high, *i.e.*, of around 0.05 kcal mol^−1^ K^−1^. This may be impacted by explicit treatment of solvent and anharmonic effects, therefore, experimental validation of DETC photolysis is essential. Such a reaction should also be kinetically accessible to compete with spontaneous IC or RISC processes, which happen with the rate of 4.50 × 10^10^ s^−1^/1.25 × 10^10^ s^−1^ and 2.44 × 10^9^ s^−1^/1.38 × 10^8^ s^−1^ from T_4_/T_6_ excited state, respectively (see Table 1). Fig. S21† illustrates the key finding regarding the formation of DETC radicals according to the mentioned reaction mechanisms. This study indicates that if DETC spontaneously form reactive radicals for FRP in 3D printing, they may originate from photolysis. Moreover, the formation of the DETC ketyl radical (shown in Fig. 1c) is possible, however, it is less reactive than other possible species (Table S32† and ref. 89 and 90).

**Fig. 6:**
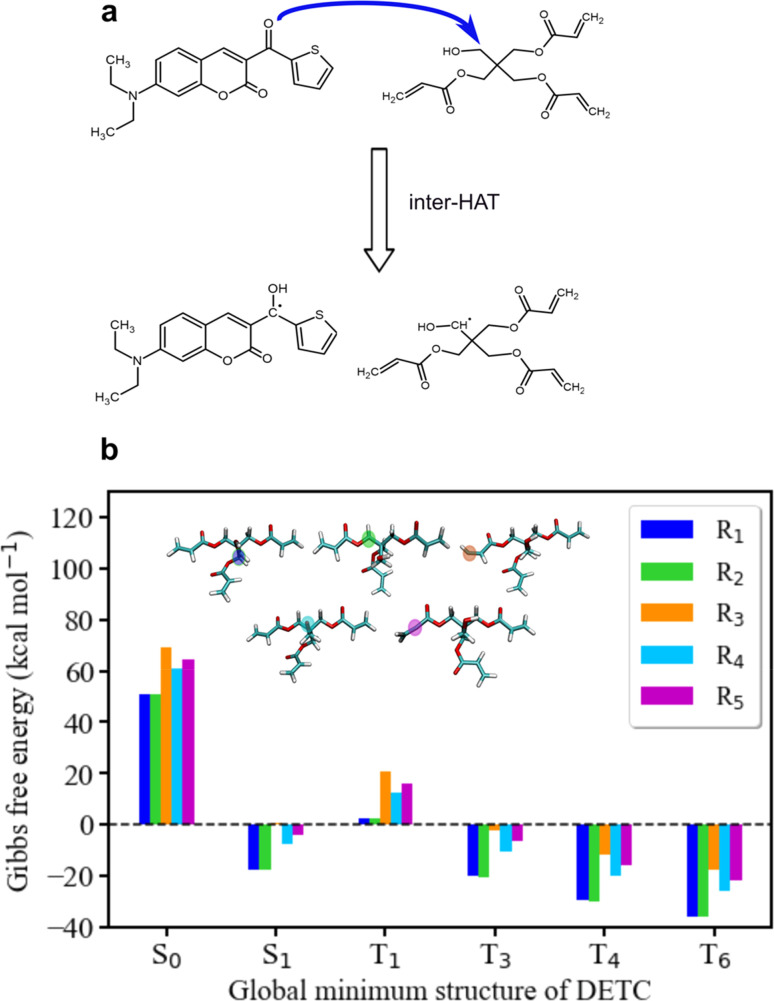
Radical reaction mechanisms between DETC and PETA. (a) HAT reaction between DETC in a high triplet state and PETA in the ground state that leads to the formation of a ketyl radical on DETC and an alkyl radical on PETA (R_1_ or R_2_) that consequently initiates the polymerization (R_1_ is reported in the figure). It is important to note that all carbon atoms in the alpha position with respect to the central carbon atom in the pentaerythritol fragment of PETA are equally susceptible to form radicals. (b) Gibbs free energy of the reaction involving different PETA radicals (R_1_–R_5_, see Fig. S18†) as a function of the electronic state of DETC (see Table S24†). Energies were computed employing (U)CAM-B3LYP-D3(BJ)/def2-TZVP approach in implicit ACN.

Since the photoresist contains also PETA monomers, we have investigated the impact of DETC on the radical formation directly by PETA. The mechanism of PETA radical formation should be similar to the reaction between DETC and the DBA co-initiator (Section 8 in ESI†). The intermolecular HAT should involve the C–H bond cleavage of the alkyl chain of PETA upon DETC attack *via* the carbonyl functional group (see Fig. 6). C–H bond cleavage of PETA without DETC possesses high BDE (the lowest value is of 84.05 kcal mol^−1^, see Table S23 and Fig. S18†) and it requires a higher number of photons to enable the 3D printing.^2,4,29^ (in general, such printing loses its resolution in comparison to the photoresists that include DETC). According to the Gibbs free energies reported in Fig. 6 and Table S24,† the C–H cleavage of PETA with the inter-HAT reaction in the presence of DETC should be easier permitted if DETC is in, *e.g.*, T_1_ state, resulting in the formation of the R_1_ or R_2_ type PETA radical. However, such a reaction is still not spontaneous.

The formation of radicals with the participation of the T_1_ state of DETC (for photoresists without co-initiator) was suggested in several reports.^4,16,17,91^ To our knowledge, there is no experimental evidence of such radicals formed or reaction mechanisms reported. Since the Gibbs free energy of a similar HAT reaction between DETC (in T_1_) and DBA is even lower than for the reaction with PETA (0.44 kcal mol^−1^*vs.* 5.47 kcal mol^−1^, see Table S25†) and this reaction could not be observed in the printing conditions (see Fig. 3b) (even if the reaction rate is 7.33 × 10^7^ s^−1^ as calculated using the activation energy barrier presented in Fig. S19†), we tend to believe that the HAT reaction between PETA and DETC should not occur, when DETC is in T_1_ excited state. The Gibbs free energy for this reaction when DETC is in higher triplet states is significantly reduced, *i.e.*, even to −29.47 kcal mol^−1^ or to −35.62 kcal mol^−1^ for DETC in T_4_ and T_6_ triplet states, respectively (Fig. 6b and Table S25†). Thus, they are more prone to compete with internal photophysical processes in DETC, suggesting reactions to happen from such states. Due to the convergence problems in optimizing transition states involving PETA and DETC in high triplet states, we cannot provide rates of such reactions, however, the radical formation pathway mentioned helps in rationalizing experimental observations. It should be noted that the calculated rates and Gibbs free energies represent values in equilibrium conditions without population distribution of excited states, various decay pathways, light intensity *etc.* They were aimed to reveal molecular bases of the complex processes during TPP with DETC.

From all reaction pathways examined, we conclude that DETC may be susceptible to radical generation through the inter-HAT involving the PETA monomer or photolysis, which are both spontaneous even in the absence of a co-initiator. Experimental confirmation of an exact radical species by, *e.g.*, the in-depth analysis of the chemical composition of the 3D laser printed materials is still necessary. Since these processes are more spontaneous with the participation of DETC in higher triplet states, reached upon TA, the third-order dependency and exceptional efficiency of this PI in FRP are experimentally observed.

**Fig. 7:**
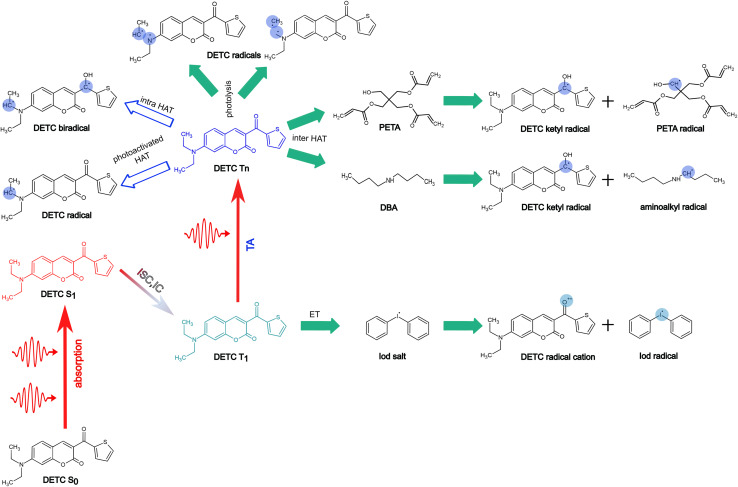
Summary of radical formation mechanisms during 3D printing in the presence of the DETC photosensitizer considered in the present study. Multiphoton excitation of DETC to the first singlet excited state S_1_, subsequent ISC to triplet T_3_ and IC to triplet T_1_ followed by TA to higher triplet states are depicted. Radical formation pathways, involving T_1_ and T_4_ excited states; ET (electron transfer) with onium salt (diphenyliodonium hexafluorophosphate, DPIHFP) involves T_1_ of DETC, thus nonlinearity of *N* ∼ 2, whereas higher triplet state(s) should activate inter-HAT reactions with PETA or DBA co-initiator or photolysis, scaling with *N* ∼ 3. Formation of aminoalkyl DETC radicals or biradicals *via* photoactivated HAT is hindered.

In Fig. 7, we summarize pathways of the multiphoton activation of DETC in 3D printing with fs lasers of the wavelength of ∼800 nm, as well as the radical generation mechanisms considered in this work. Pathways, resulting in possibly the most active radical formation, are marked with the filled green arrows, whereas pathways that are hindered due to various reasons, explained in Section 9 in ESI† (*i.e.* DETC radicals), are marked with empty blue arrows. Note that different PETA radicals with the activated carbon atoms in the alpha position with respect to the central carbon atom in the pentaerythritol fragment of PETA can occur. The mechanisms of FRP are explained in detail in Section 10 in ESI.† More investigation from an experimental point of view is, therefore, necessary to further validate theoretically predicted radical formation mechanisms and the reactivity of DETC in its T_1_ state in comparison to the higher triplet states.

## Conclusions

3D laser nanoprinting techniques and photoresist systems have advanced significantly over the past decade. A consensus has emerged that the PI is a key ingredient in the complex interplay of factors that control the speed and resolution of 3D laser nanoprinting. However, the molecular basis of photoactivation during 3D printing is still not well understood. Herein, we have provided an in-depth analysis of the photoactivation and photoreactivity of DETC, one of the most efficient, presently used, multiphoton photoinitiators, to explain the mechanisms of FRP in 3D printing using DETC. We have demonstrated the second-order scaling of the DETC reactivity with DPIHFP onium salt and provided a molecular understanding of the formation of radicals in a two-component bimolecular system comprising DETC and the DBA co-initiator as well as DETC and the PETA monomer.

We have shown that in a one-component system, *i.e.*, in the absence of a co-initiator, the FRP initiation with nonlinearity of 2 is not allowed, since the electronic structure of DETC in the T_1_ excited state does not permit active radical formation, which typically occurs for unimolecular processes for Norrish type I PIs. The BDE of C–H, C–C or C–N bond cleavage of DETC, aimed to open the pathway for reactive radicals, is too high in T_1_ (∼22–51 kcal mol^−1^). However, bond breaking affinity of DETC changes significantly upon TA absorption of the third photon at *ca.* 800 nm. This wavelength fits well to the triplet–triplet transition of DETC from its T_1_ to higher triplet states, *e.g.*, T_4_, modulating the electronic structure of DETC towards easier H-atom abstraction reactions or C–C and C–N bond cleavage. However, H-atom abstraction-based hypothetical pathways for the radical creation are not spontaneous and cannot compete with fast internal conversion to T_1_, hindering the formation of active DETC radicals and biradicals during 3D printing that could directly initiate FRP. The Gibbs free energy of photolysis of DETC in high excited states shows higher spontaneity (*i.e.*, −0.52 to −26.96 kcal mol^−1^ from the T_4_ excited state), however further experimental validation is required.

We demonstrate that the radicals that could, indeed, initiate FRP (especially in the absence of a co-initiator) are alkyl-based radicals of PETA, formed upon H-atom abstraction reaction between from PETA to DETC. The rate of this reaction, from the T_1_ excited state of DETC, should be lower than the reaction of DETC with DBA (*i.e.*, the rate of 7.33 × 10^7^ s^−1^). Since the latter is not observed experimentally, we demonstrate that the formation of reactive radicals occurs in the presence of DETC excited to higher triplet states. In both cases, for PETA and DBA, HAT with DETC in high triplets should be spontaneous. When these reactions are capable of competing with photophysical processes, such as IC and RISC, they will initiate FRP according to mechanisms suggested in this study.

The provided in-depth investigation of DETC photosensitization permits the understanding of the pathways initiating polymerization of photoresist compositions comprising DETC during 3D printing. It addresses ongoing challenges in rapid 3D laser nanoprinting by providing possible ways for more sensitive PIs, such as enhanced capabilities of HAT abstraction either from a co-initiator or a monomer used in a such photopolymerization.

## Data availability

The raw data of this publication are permanently available on our institutional repository and can be referenced *via* this link https://www.radar-service.eu/radar/en/dataset/KjeSQpjIkSHeqhFY?token=ZOuvquDpJMfFJJufuEfO.

## Author contributions

A. M.: quantum mechanical calculations, investigation, data analysis and visualization, drafting the paper, paper review and editing; P. K.: nonlinearity measurements, experiment-theory feedback loops and analysis, paper review and editing; P. N.: discussion, data interpretation, paper review and editing; T. M.: nonlinearity measurements, discussion and data interpretation, paper review and editing; N. M. B: discussion, data interpretation, paper review and editing; L. Y.: discussion, data interpretation, paper review and editing; S. W.: discussion, data interpretation, paper review and editing; A. N. U.: discussion, data interpretation, paper review and editing; C. B. K.: discussion, data interpretation, paper review and editing; M. W.: discussion and data interpretation, paper review and editing; W. W.: discussion and data interpretation, funding acquisition, resources, paper review and editing; M. K.: conceptualization, supervision, project administration, investigation, data analysis, funding acquisition, paper writing, review and editing.

## Conflicts of interest

There are no conflicts to declare.

## Supplementary Material

SC-OLF-D4SC03527E-s001
