# Supplementary material for: Two- and three-photon processes during photopolymerization in 3D laser printing
Source: Chem Sci. 2024 Jul 15;15(32):12695–709. doi: 10.1039/d4sc03527e (PMC11309088; doi:10.1039/d4sc03527e)
Supplement: SC-015-D4SC03527E-s001 [file SC-015-D4SC03527E-s001.pdf]

Electronic Supplementary Material (ESI) for Chemical Science.
This journal is © The Royal Society of Chemistry 2024

Supplementary Information

Two- and three-photon processes during photopolymerization in 3D laser printing

Anna Mauri[1], Pascal Kiefer[2], Philipp Neidinger[3,4] Tobias Messer[2], N. Maximilian Bojanowski[1,2,$], Liang Yang[1,2,#], Sarah Walden[4], Andreas-Neil Unterreiner[3], Christopher Barner-Kowollik[1,4], Martin Wegener[1,2], Wolfgang Wenzel[1], Mariana Kozlowska[1,*]

[1] Institute of Nanotechnology (INT), Karlsruhe Institute of Technology (KIT), Germany
[2] Institute of Applied Physics (APH), Karlsruhe Institute of Technology (KIT), Germany
[3] Institute of Physical Chemistry (IPC), Karlsruhe Institute of Technology (KIT), Germany
[4] School of Chemistry and Physics, Queensland University of Technology (QUT), Brisbane, Australia
[$]Present address: Organic and Carbon Nanomaterials Unit, Okinawa Institute of Science and Technology Graduate University, Japan
[#]Present address: Suzhou Institute for Advanced Research, University of Science and Technology of China (USTC), 215127 Suzhou, China
[*] corresponding author

Table of Contents

1. Ground state properties of DETC. Absorption and emission spectra **1**
2. Comparison of DFT functionals **4**
3. Transition orbitals. Intramolecular charge transfer analysis **8**
4. Two- and three-photon spectra of DETC **14**
5. Optimization of excited states of DETC **17**
6. Radiative and nonradiative rates **26**
7. Triplet-triplet absorption spectra **36**
8. Reactivity of DETC with a co-initiator and PETA **39**
8.1 Reaction of DETC with amine co-initiator 40
8.2 Reaction of DETC with PETA 41
9. DETC radical formation mechanisms **43**
9.1. H-abstraction of DETC 43
9.2. Photolysis of DETC 45
9.3. Biradicals formation of DETC 46
10. Polymerization mechanism **50**
References **53**

1. Ground state properties of DETC. Absorption and emission spectra

All calculations were performed using Density Functional Theory (DFT) and Time-Dependent DFT (TD-DFT) methods using Gaussian16 Rev. C.01[1]. For the selected properties of DETC (7-diethylamino-3-thenoylcoumarin) in excited states, MOMAP Version 2022A (2.3.3)[2–7], ADF 2020.1[8], Dalton 2020.0[9], Dynavib[10,11] and TURBOMOLE V7.4[12] codes were also used. This is denoted in detail in the respective section.

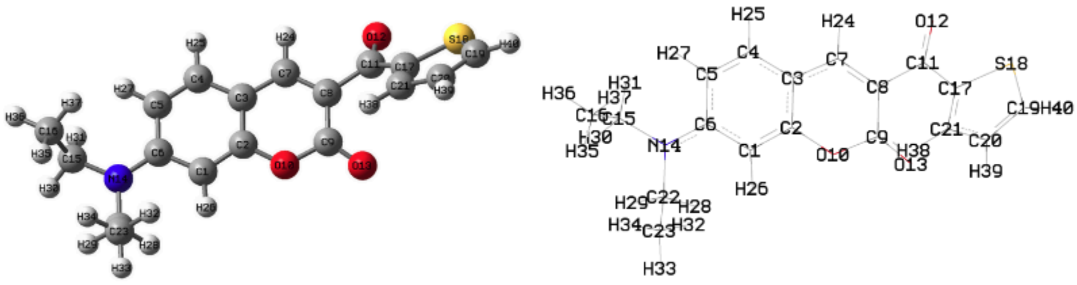

Fig. S1: Ground state structure of DETC photoinitiator optimized using CAM-B3LYP in *implicit* Acetonitrile, ACN, (PCM model)[16] (left panel). DETC molecule with labeled atom types and atom numbers (right panel). The coordinates of this structure are available via DOI: 10.35097/1056. It also contains structures of excited states, which are analyzed in Section 5.

Table S1: Vertical excitation energies for the first ten singlet and triplet excitations **in ACN** starting from optimized ground state geometry (S_0) using **CAM-B3LYP[17]-D3(BJ)/def2-TZVP** within a time-dependent (TD) approach. Oscillator strength values for triplet excitations are zero and therefore not reported in the table.

	**Singlet excitation (ACN)**			**Triplet excitation (ACN)**	
**State**	Energy (eV)	Wavelength (nm)	Oscillator strength	Energy (eV)	Wavelength (nm)
**Excited State 1**	**3.45**	**359.30**	**0.9611**	2.36	525.77
Excited State 2	4.06	305.52	0.0505	2.84	436.56
Excited State 3	4.35	285.10	0.0296	3.52	352.61
Excited State 4	4.56	272.09	0.0217	3.77	328.77
Excited State 5	4.66	266.19	0.1405	3.80	326.27
Excited State 6	4.92	251.90	0.0647	3.96	312.81
Excited State 7	4.99	248.36	0.0756	4.04	306.70
Excited State 8	5.25	236.06	0.0160	4.25	291.61

Excited State 9	5.55	223.33	0.0613	4.31	287.87
Excited State 10	5.61	221.01	0.0909	4.87	254.44

Table S2: Vertical excitation energies for the first ten singlet excitations **in ACN** (COSMO *implicit* solvation) starting from optimized ground state geometry (S_0). Excitations were computed In TURBOMOLE v7.4[12] using **eigenvalue-only self-consistent GW**[18,19] (evGW) calculations, employing the contour deformation (CD) variant with CAM-B3LYP functional with def2-TZVP basis set and def2-TZVP auxiliary basis set. Resolution of Identity (RI) approximation for the Coulomb term was also included. The solvation effects were included via the solvated orbitals, i.e. the omega_0 term.

	**Singlet excitation evGW-BSE**		
**State**	Energy (eV)	Wavelength (nm)	Oscillator strength
Excited State 1	**3.25**	**381.91**	**0.87**
Excited State 2	3.82	324.44	0.04
Excited State 3	4.07	304.51	0.08
Excited State 4	4.26	290.87	0.03
Excited State 5	4.34	285.53	0.02
Excited State 6	4.52	274.16	0.12
Excited State 7	4.76	260.74	0.03
Excited State 8	4.96	249.75	0.00
Excited State 9	5.41	229.07	0.11
Excited State 10	5.47	226.64	0.02

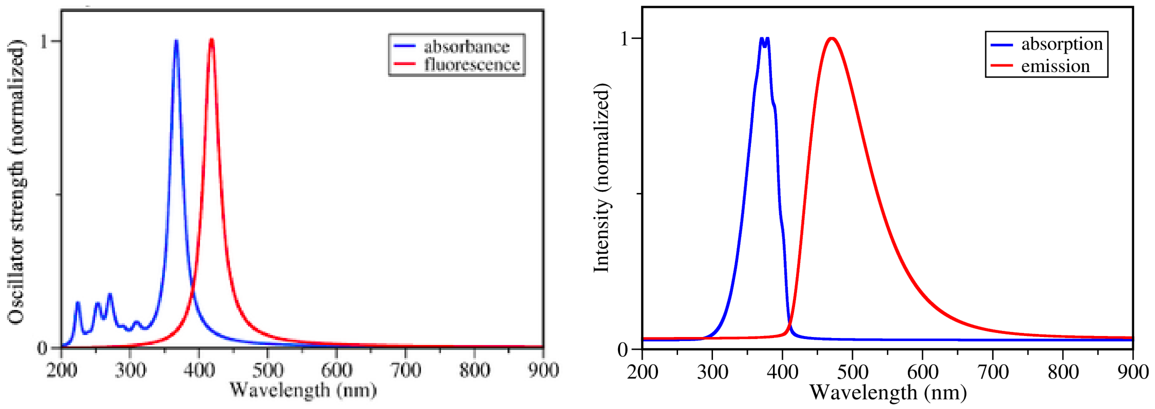

Fig. S2: Calculated absorption and emission (fluorescence) spectra of DETC in implicit ACN using TD-CAM-B3LYP-D3(BJ)/def2-TZVP (left) and including vibronic effects (right). Vibrationally-resolved spectra were calculated within the Franck-Condon (FC)[20–22] approximation at 100K with Adiabatic Hessian (AH-G16) as the PES model in Dynavib. Spectra without the inclusion of vibronic effects were plotted using half width at half maximum (HWHM) of 0.1 eV and Lorentzian type broadening function. Vibrationally-resolved absorption spectrum was plotted with HWHM of 80 cm^{-1} (i.e. 0.01 eV), convergence factor of $1.0x10^{-4}$ and Lorentzian type broadening function.

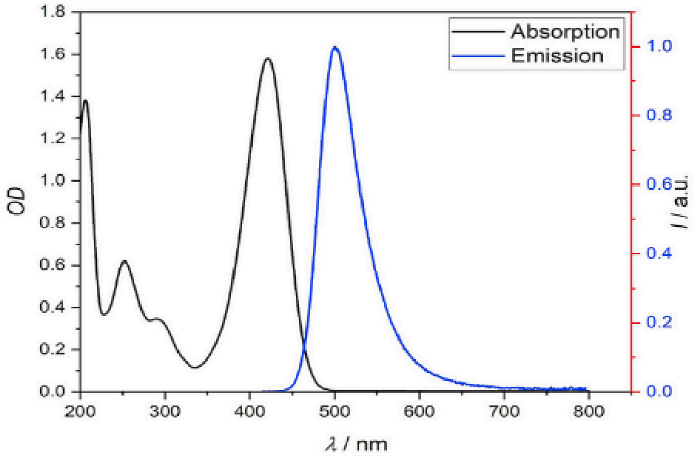

Fig. S3: Experimental absorption and emission (fluorescence) spectra of DETC in ACN. Maxima of absorption and emission are at 421 nm and 500 nm, respectively.

2. Comparison of DFT functionals

Table S3: Values of absorption and emission peak maxima obtained experimentally and the respective ½ Stokes shift (i.e. 0-0 energies)[23,24] of DETC in PETA and ACN (see Fig. S3). Comparison with energies obtained by different DFT functionals is reported in Table S4.

	**PETA**			**ACN**		
	**Absorption**	**Emission**	**½ Stokes shift (E_{0-0})**	**Absorption**	**Emission**	**½ Stokes shift (E_{0-0})**
**eV**	2.90	2.52	**2.71**	2.94	2.48	**2.69**
**nm**	427	492	457.5	421	500	460.5

Table S4: Values of E_{0-0} energies (v_{00} i.e., between the ground vibrational states of the two electronic states) and the corresponding wavelengths of DETC obtained using B3LYP and CAM-B3LYP functionals with def2-TZVP basis set within linear response (LR) time-dependent (TD) approach. ½Stokes shift from experiment (Table S3) was compared to 0-0 energies[23,25], therefore only absorption values are listed.

**Energy / λ**	**B3LYP**	**CAM-B3LYP**
**E_{0-0} gas (eV)**	2.73	3.39
**λ gas (nm)**	454	366
**E_{0-0} ACN (eV)**	**2.54**	**3.04**
**λ ACN (nm)**	488	409

Table S5: Values of the vertical excitation energies of DETC in the gas phase, ACN and PETA, obtained using different DFT functionals and def2-TZVP basis set within a linear response time-dependent (TD) approach starting from the geometry obtained as a single point on the optimized B3LYP geometry. Values for the CAM-B3LYP functional were obtained both using B3LYP-optimized geometry (SP CAM-B3LYP) and upon optimization with CAM-B3LYP (CAM-B3LYP).

	**B3LYP**	**SP CAM-B3LYP**	**CAM-B3LYP**
**Vertical energy gas (eV)**	3.23	3.62	3.69
**Vertical wavelength gas (nm)**	384	342	336

**Vertical energy ACN (eV)**	2.93	3.38	3.45
**Vertical wavelength ACN (nm)**	423	367	359
**Vertical energy PETA (eV)**	3.00	3.49	-
**Vertical wavelength PETA (nm)**	401	355	-

Table S6: Vertical excitation energies of DETC in *implicit* ACN computed in Gaussian16 employing linear-response (LR), cLR[26] (corrected linear-response), and state-specific (SS) approaches such as External Iteration[27] (EI). The experimentally estimated absorption maximum at ACN is 421 nm (2.94 eV). Data with CAM-B3LYP were computed starting from the geometry obtained as a single point on the optimized B3LYP geometry (marked as SP CAM-B3LYP) and upon optimization with CAM-B3LYP (marked as CAM-B3LYP).

	**LR**		**cLR**		**EI**	
	**eV**	**nm**	**eV**	**nm**	**eV**	**nm**
**B3LYP**	2.93	422.64	2.86	433.22	2.27	544.70
**SP CAM-B3LYP**	3.38	367.04	3.42	362.33	3.20	387.94
**CAM-B3LYP**	3.45	359.30	3.49	355.26	3.25	381.96

From data reported in Tables S4-S6, a slightly larger blue-shift can be observed for DETC in the gas phase caused by the lack of the proper polarization environment induced by the solvent. 1PA and 0-0 energies obtained using long-range corrected CAM-B3LYP[17] showed higher or equivalent deviations (i.e. for 1PA in ACN +0.43 eV, while for 0-0 energies +0.34 eV) from experimental values than B3LYP (-0.01 and -0.09 eV for 1PA and 0-0 energy). 0-0 energies were compared to the ½ Stokes shift calculated from experimental spectra (Table S3, S4).

Table S7: Comparison of the emission energy (fluorescence) computed with B3LYP and CAM-B3LYP functionals using several approaches. The S_0 and S_1 geometry were optimized first with DFT and TD-DFT using the respective functional. The equilibrium corrected linear response (cLR eq.), non-equilibrium cLR (cLR noneq.), and non-equilibrium External iteration (EI noneq.) were computed as a single point on the S_1 minimum geometry obtained with TD-DFT with both functionals. Vibronic emission could not be computed with B3LYP due to the fact that S_0 and S_1 are not harmonically related (see Fig. S9).

	TD-DFT		TD-DFT vibr.		EI noneq*		cLR noneq**		cLR eq***	
	nm	eV	nm	eV	nm	eV	nm	eV	nm	eV
B3LYP	553	2.24	-	-	1878	0.66	1512	0.82	961	1.29

CAM-B3LYP	418	2.96	470	2.64	420	2.95	394	3.15	390	3.18

*Emission state-specific solvation external iteration approach (non-equilibrium solvation based)
**Emission corrected linear-response approach (non-equilibrium solvation based)
***Emission corrected linear-response approach (equilibrium solvation based: the equilibrium solvation of the excited state S_1 at its equilibrium geometry)

From data reported in Table S7, we can observe that, although TD-DFT results seem to be better with B3LYP compared to experiment (emission at 553 nm using B3LYP vs 418 nm in CAM-B3LYP), the cLR eq., cLR noneq. and EI noneq. (SS effects) significantly influence these results, turning them into physically not consistent data for B3LYP, which cannot be compared with experiments. From (non)-equilibrium corrected linear response (cLR eq and noneq) data, it can be clearly seen that the energy obtained with B3LYP (i.e 1.29 eV and 0.82 eV) is too low when compared to experimental emission of 2.48 eV. **Data obtained with CAM-B3LYP, although with a slightly larger shift of the maximum peak absorption than for the B3LYP (in TD-DFT), keeps physical consistency. This observation motivated us to use the CAM-B3LYP functional to calculate DETC**. Further comparison of data generated by both functionals is given in Tables S8, S9, S11, S12.

Table S8: Adiabatic and 0-0 energy values computed with B3LYP and CAM-B3LYP functionals with LR and cLR approaches.

	B3LYP using B3LYP optimized structures		CAM-B3LYP using CAM-B3LYP optimized structures	
	eV	nm	eV	nm
LR E_adiabatic	2.60	476.86	3.11	399.17
LR E 0-0	2.54	487.36	3.04	407.44
Δ ZPVE (eV)	-0.002		-0.063	
cLR E_adiabatic	1.65	751.88	2.89	429.46
cLR E 0-0	1.65	752.79	2.82	439.04

Results reported in Table S8 show that the energy obtained by the B3LYP functional after the addition of i.e. cLR is decreasing (1.65 eV) and significantly deviates from the crossing point between absorption and emission in experiment (i.e. of 2.69 eV). The data with CAM-B3LYP are more consistent (2.82 eV vs 2.69 eV in experiment).

Table S9: Fluorescence peaks of DETC in ACN. Note: emission in the experiment was reported to be at 492 nm in PETA and 500 nm in ACN.

Fluorescence			
	**Wavelength, nm**	**Energy, eV**	**Oscillator strength**
CAM-B3LYP, ACN	418.17	2.96	1.2973
B3LYP, ACN	553.78	2.24	0.0093
B3LYP, gas phase	625.67	1.98	0.0008

Even if B3LYP reproduces absorption characteristics of DETC for the best (see Table S4, S5), similarly was also shown by the set of benchmark calculations by e.g. Fang et al[30], we see that it works worse than other functionals at capturing emission processes (see Table S7). This is a reason for the low fluorescence rate, as listed in Table S9 (the oscillator strength is much lower than with the CAM-B3LYP functional). It might also be connected to the unexpected larger difference between the vertical and 0-0 energies in the gas phase (3.23-2.73=0.50 eV) than in ACN (2.93-2.54=0.39 eV). The relaxation changes with CAM-B3LYP functional are captured correctly, i.e. the differences between the vertical and 0-0 energies are: 3.62 - 3.39 = 0.23 eV in the gas phase and 3.38 - 3.04 = 0.34 eV in ACN (Table S4, S5). The mean absolute error of 0–0 transitions using TD-DFT in the present study is in the range of 0.19-0.27 eV based on comparison to experiments and high level theory CC2 calculations[31–36].

In addition, we compared the quality of the TD-DFT approach for the vertical excitation energies of DETC to the GW approximation and Bethe-Salpeter equation (BSE) approach. For this, we have used GW-BSE as implemented in TURBOMOLE v7.4. Vertical excitation energies were corrected by eigenvalue-only self-consistent GW (evGW) calculations, employing the contour deformation (CD) variant[37], where the Green's function $G0$ was computed from the DFT wavefunction and the quasiparticle eigenvalues were updated iteratively in the calculation of G and W (evGW). We have used the scheme reported in Ref.[38], where firstly the electron self-energy operator within the GW approximation is constructed and the one-particle Green's function is calculated, yielding the quasi-particle energies that correspond to single-electron ionization energy and electron affinity[39]. In a second step, the two-particle interaction is constructed, and the BSE for the two-particle Green's function is solved, resulting in the neutral excitation energies[39,40].
Calculations of GW-BSE triplet vertical excitation energies of DETC were performed in TURBOMOLE v7.4 starting from S_0 (Table S2) and T_1 (Table S22) geometry. For these calculations the def2-TZVP basis set and def2-TZVP auxiliary basis set[41,42], as well as the Resolution of Identity (RI) approximation[43,44] were employed. The convergence criterion of SCF energies was set to 10^{-8} Hartree.

3. Transition orbitals. Intramolecular charge transfer analysis

Significant electron density delocalization of DETC all over the π-conjugated system is demonstrated by molecular orbitals (MOs, Fig. S4-S6) and the charge transfer analyses (Table S12). They are associated with singlet and triplet excitations reported in Fig. S2 and Table S1. Specifically, the Highest Occupied Molecular Orbital (HOMO) and the Lowest Unoccupied Molecular Orbital (LUMO) are strongly delocalized, showing the ππ* character associated with the $S_0 \rightarrow S_1$ transition of DETC, contributing to the observed low-energy spectral line. A weaker $S_0 \rightarrow S_2$ absorption band centered at 306 nm corresponds also to strongly delocalized electron density on π-scaffold representing HOMO→LUMO+1 transition. We observe the nπ* character for transitions $S_0 \rightarrow S_2$ and $S_0 \rightarrow T_3$, while the $S_0 \rightarrow T_{1,2,4,5,6}$ transitions are characterized by ππ* character with different grades of delocalization all over the molecule (see Fig. S4-S6).

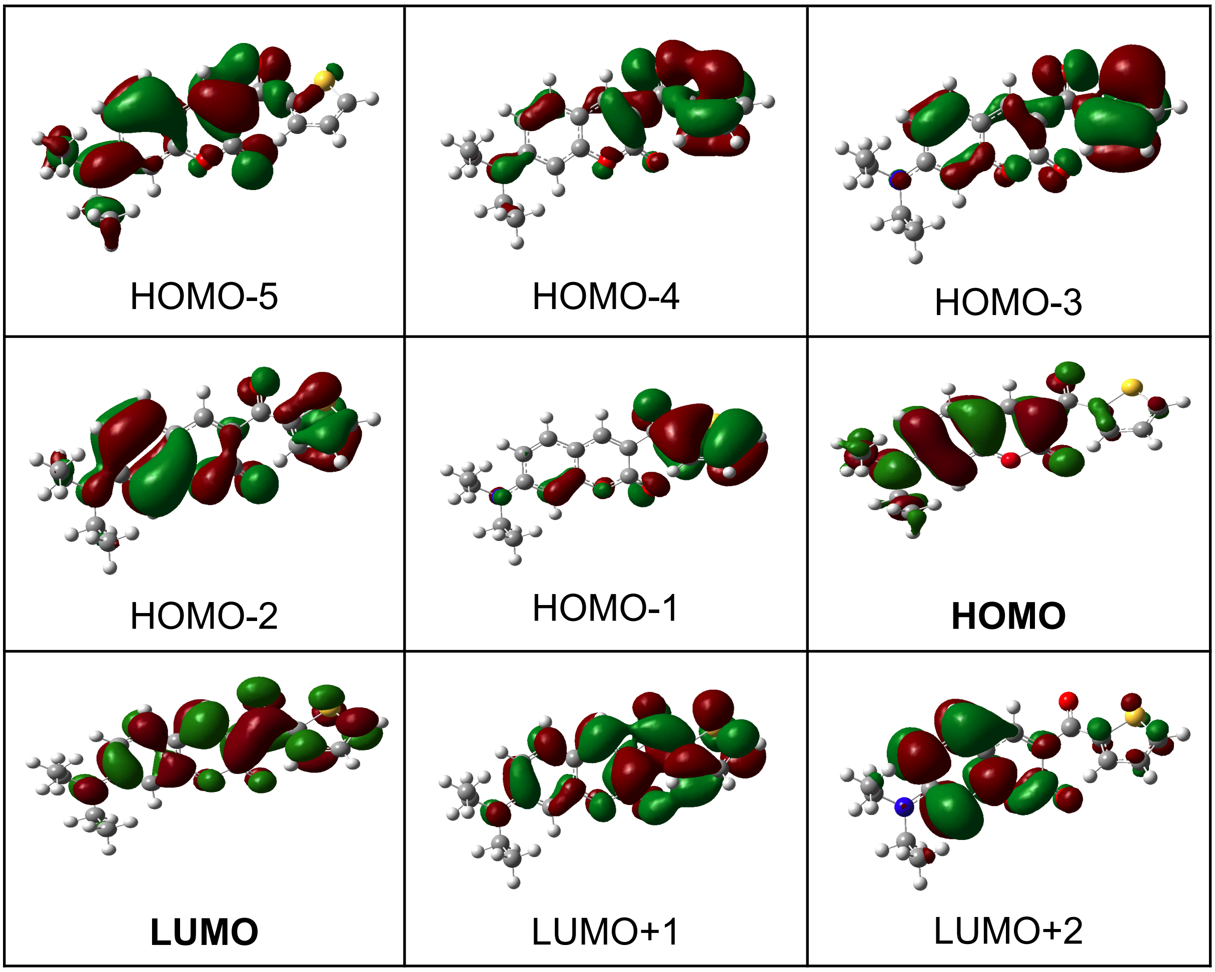

Fig. S4: Visualization of molecular orbitals of DETC in ACN obtained using CAM-B3LYP-D3(BJ)/def2-TZVP level of theory. HOMO and LUMO correspond to the Highest Occupied Molecular Orbital and Lowest Unoccupied Molecular Orbital, respectively. Isovalue of 0.002 a.u. was used for visualization.

Table S10: Transition orbitals of DETC **in ACN** with CAM-B3LYP-D3(BJ)/def2-TZVP using the single point calculation on the B3LYP optimized ground state structure. The respective contributions involved in the S_0–$S_{1,}$ S_0–$S_{2,}$ S_0–$T_{1,}$ S_0–T_2 ,S_0–T3, S_0–$T_{4,}$ S_0–$T_{5,}$ S_0–$T_{6,}$ and S_0–T_7 transitions are listed in Table S1.

Transition	Molecular Orbitals	Contribution (%)
$S_0 \rightarrow S_1$	HOMO → LUMO	97
$S_0 \rightarrow S_2$	HOMO → LUMO +1	94
$S_0 \rightarrow T_1$	HOMO → LUMO	72
$S_0 \rightarrow T_2$	HOMO-1 → LUMO<br>HOMO -1 → LUMO +1	45<br>40
$S_0 \rightarrow T_3$	HOMO -4 → LUMO<br>HOMO -3 → LUMO<br>HOMO -5 → LUMO<br>HOMO → LUMO +1	21<br>20<br>18<br>19
$S_0 \rightarrow T_4$	HOMO -2 → LUMO<br>HOMO → LUMO +2<br>HOMO -2 → LUMO +1	32<br>19<br>11
$S_0 \rightarrow T_5$	HOMO -3 → LUMO +1<br>HOMO -3 → LUMO<br>HOMO -2 → LUMO	22<br>20<br>20
$S_0 \rightarrow T_6$	HOMO-5→ LUMO<br>HOMO → LUMO +3<br>HOMO → LUMO +2<br>HOMO -4 → LUMO +1	17<br>17<br>15<br>15
$S_0 \rightarrow T_7$	HOMO-2 → LUMO<br>HOMO → LUMO+1<br>HOMO → LUMO	12<br>33<br>10

Table S11: Transition orbital of DETC **ACN** with respective contributions involved in the S_0–S_1 transition using the B3LYP functional.

Transition	Molecular Orbitals	Contribution (%)
$S_0 \rightarrow S_1$	HOMO → LUMO	97

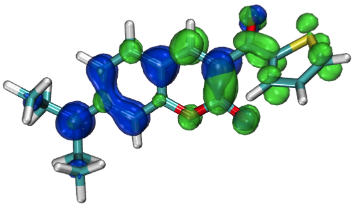

Fig. S5: Visualization of electron donating (hole, in blue) and electron accepting (electron, in green) density transfer upon the excitation of DETC from the ground state to the first singlet state **in ACN**. Electron-hole analysis and visualization of the respective contributions were performed using Multiwfn (version 3.6) analyzer[40,41] based on data obtained using TD-B3LYP-D3(BJ)/def2-TZVP level of theory. Isovalue of 0.003 a.u. was used for visualization.

TDDFT CAM-B3LYP ACN (PCM)	
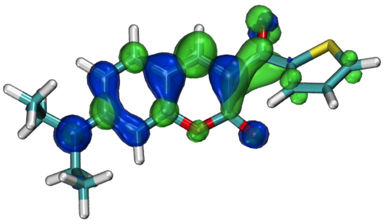 S_1	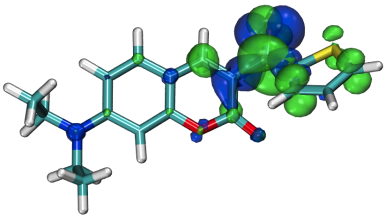 S_2
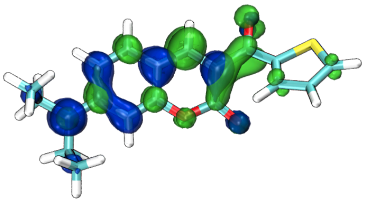 T_1	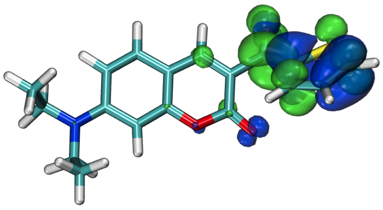 T_2
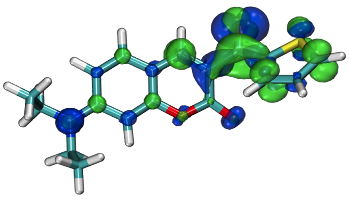 T_3	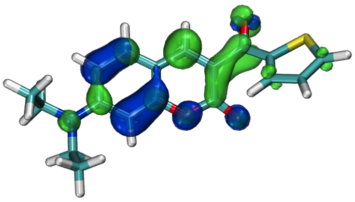 T_4

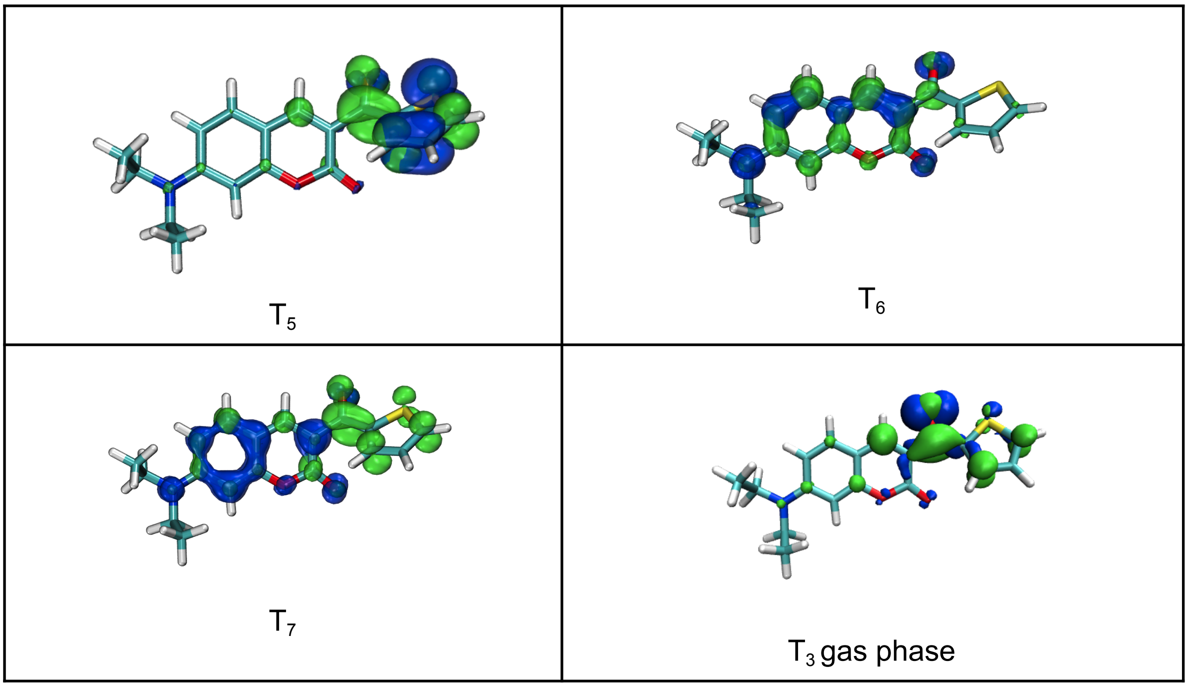

Fig. S6: Visualization of electron donating (hole, in blue) and electron accepting (electron, in green) density transfer upon the excitation of DETC from the ground state to the singlet and triplet states **in ACN**. Electron-hole analysis and visualization of the respective contributions were performed using Multiwfn (version 3.6) analyzer[40,41] based on data obtained using TD-CAM-B3LYP-D3(BJ)/def2-TZVP level of theory. Singlet excited and triplet excited states are labeled with S and T, respectively. Isovalue of 0.003 a.u. was used for visualization.

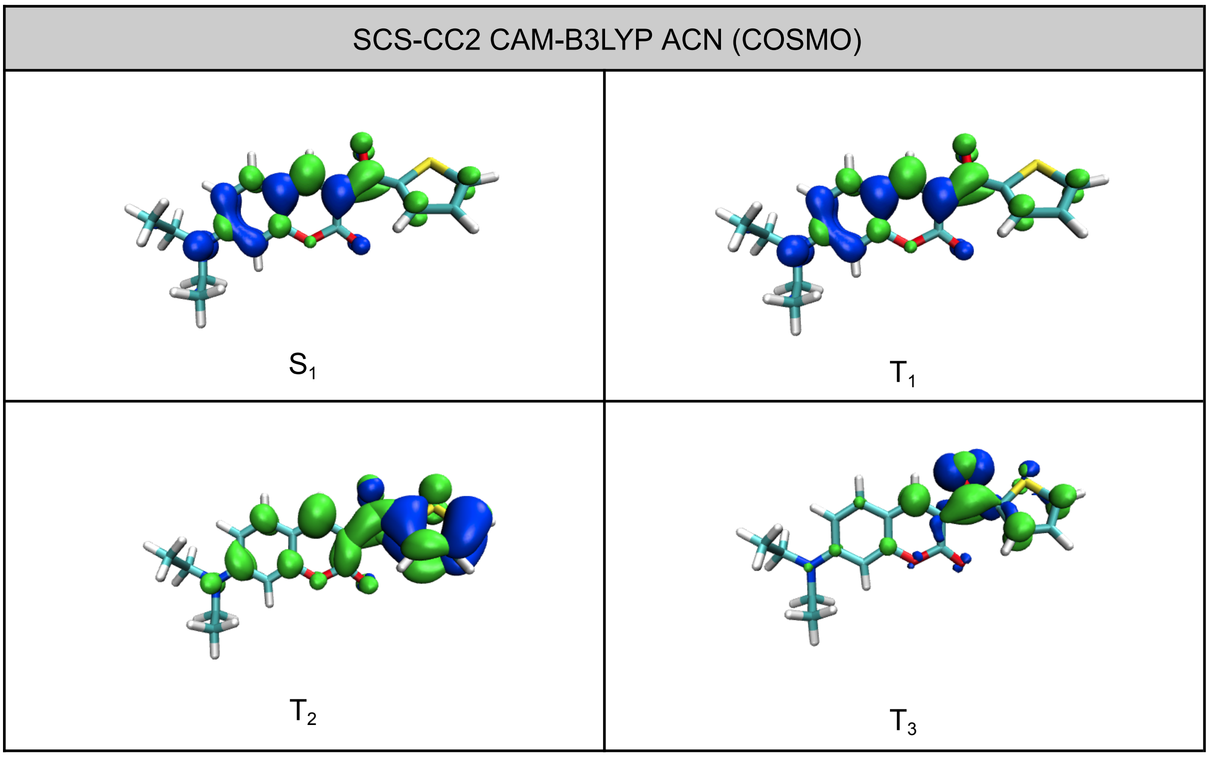

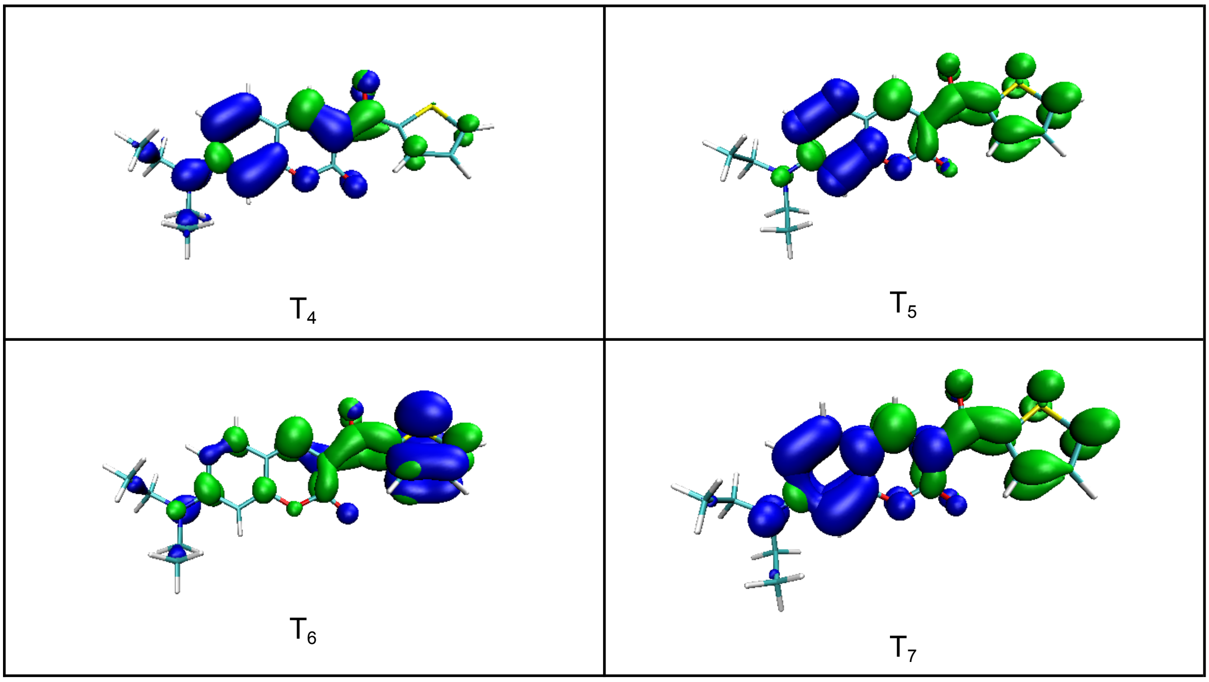

Fig. S7: Visualization of electron donating (hole, in blue) and electron accepting (electron, in green) obtained using SCS-CC2 (spin-component scaled coupled cluster) in ACN (COSMO) with the CAM-B3LYP optimized structure. Singlet excited and triplet excited states are labeled with S and T, respectively. Isovalue of 0.003 a.u. was used for visualization.

From holes and electrons analysis reported in Fig. S6 can be seen that the HOMO → LUMO excitation for S_1 and T_1 includes mostly charge transfer from nitrogen atom of alkylamine chain (atom N14) to the carbonyl functionality (atoms C11-O12). It agrees with the density transition obtained in the SCS-CC2 method. Charge transfer for triplet excited state T_3 and T_4 is mostly localized on carbonyl bond C11-O12 while charge transfer for T_2 and high triplet excited states, i.e. T_5-T_7 involves mainly the thiophenyl ring and the alkylamine chain (N14, C15, C16, C22 and C23).

Note that in the case of the T_2 state, the electron density difference using CAM-B3LYP differs from the SCS-CC2 transition, where the electron part is more delocalized within DETC. Due to the very low energy of this state in CAM-B3LYP (~1.30 eV in comparison to the S_0 state) and further convergence problems during ISC rate calculations involving this state, we have not considered the T_2 state in further calculations. Similar difference is also visible for the T_5 state in CAM-B3LYP. The optimization of this state was always connected with the breaking of the DETC structure, suggesting TD-DFT methodologic limitations for these excited states.

The vertical excitations of DETC were extensively analyzed based on CT parameters[42] (see Table S12). All transitions exhibit relatively large values of the average distribution of electrons and holes (H). Transitions $S_0 \rightarrow T_2$, $S_0 \rightarrow T_3$ and $S_0 \rightarrow T_5$ are characterized by lower H values (below 2.9 Å) with respect to the others, due to a localized distribution of holes and electrons in a specific region (refer to Fig. S6). Conversely, for other transitions, the wider distribution of holes and electrons results in higher H indices. Most other transitions display negative t values, except for $S_0 \rightarrow T_1$, which shows a slightly positive value, indicating a certain degree of separation between its hole and electron. Transitions $S_0 \rightarrow S_1$, $S_0 \rightarrow T_1$ and $S_0 \rightarrow T_7$ exhibit less negative t parameters, suggesting higher CT. The remaining transitions have highly negative t values, indicating a mixed character of CT and local exciton (LE)

transitions, as confirmed by Fig. S6. The D parameter, indicating the distance between the main distribution regions of holes and electrons, shows high average values, exceeding 0.5 for all transitions. Specifically, $S_0 \rightarrow S_1$ (B3LYP) and $S_0 \rightarrow S_1$, $S_0 \rightarrow T_1$ and $S_0 \rightarrow T_7$ (CAM-B3LYP) have the highest D values, confirming their higher CT character, while others exhibit a more mixed CT and LE character. Additionally, the overlap of electrons and holes (Sr) exceeds 0.5 in all cases, showing a certain degree of localized and CT, i.e., hybrid (mixed), character for most transitions.

In the case of B3LYP, although computed absorption spectra are in good agreement with experiment (see Tables S4,S5), the CT involved in the optimized S_1 geometry (see Fig. S9) leads to an unphysical and uncorrect reproduction of the final optimized geometry and emission properties (see Tables S7, S9), especially when involving cLR and SS corrections.

Table S12: Charge transfer (CT) data of excited states of DETC computed in B3LYP-D3(BJ)/def2-TZVP and CAM-B3LYP-D3(BJ)/def2-TZVP. Data calculated employing Multiwfn (version 3.6) analyzer. H is the average distribution of electron and hole, H_{CT} is the average degree of spatial extension of hole and electron distribution in CT direction, t is the separation degree of electron and hole in the charge transfer direction (t = D - H_{CT}), D is the total magnitude of CT length and Sr is the overlap of electrons and holes.

**DETC in ACN (B3LYP)**					
**State**	**H (Å)**	**H_{CT} (Å)**	**t (Å)**	**D (Å)**	**Sr (au)**
**S_1**	3.294	2.751	1.160	3.911	0.548
**DETC in ACN (CAM-B3LYP)**					
**State**	**H (Å)**	**H_{CT} (Å)**	**t (Å)**	**D (Å)**	**Sr (au)**
**S_1**	3.088	2.479	-0.262	2.216	0.600
**T_1**	2.969	2.309	-0.648	1.661	0.619
**T_2**	2.436	1.584	-0.795	0.789	0.652
**T_3**	2.888	2.092	-1.135	0.958	0.549
**T_4**	3.059	2.284	-1.411	0.874	0.673
**T_5**	2.690	2.084	-1.310	0.774	0.606
**T_6**	3.241	2.433	-1.753	0.680	0.649
**T_7**	3.318	2.761	-0.129	2.633	0.667

4. Two- and three-photon spectra of DETC

One-photon absorption (1PA), two-photon absorption (2PA) and three-photon absorption (3PA) spectra were computed in the gas phase (3PA) and *implicit* solvent (PCM model) using CAM-B3LYP-D3(BJ)/def2-TZVP in Dalton 2020.0. The macroscopic 2PA and 3PA cross sections, σ_{2PA} and σ_{3PA}, were calculated according to the following equations[43,44]:

$$< \sigma^{2PA} > \; = \; \frac{N\pi^2 \alpha a_0^5 \omega^2}{c} < \delta^{2PA} > g(2\omega, \omega_0, \Gamma), \tag{1}$$

$$< \sigma^{3PA} > \; = \; \frac{N\pi^3 \alpha a_0^8 \omega^3}{3c^2} < \delta^{3PA} > g(2\omega, \omega_0, \Gamma), \tag{2}$$

where N is an integer value, α is the fine structure constant, α0 is the Bohr radius, ω is the photon energy in atomic units, c is the speed of light, $<\delta_{2PA}>$ ($<\delta_{3PA}>$) is the rotationally averaged 2PA (3PA) strength and g(2ω,ω0,Γ) is the lineshape function related to spectral broadening effects. The rotationally averaged 2PA strength (in atomic units), $<\delta_{2PA}>$, was obtained from the 2PA transition moments S for linearly polarized light with parallel polarization and two photons of the same energy. It was calculated according to equation (3)[43,45]:

$$< \delta^{2PA} > = \frac{1}{15} \left(2 \sum_{a,b} S_{ab} S^*_{ab} + \sum_{a,b} S_{aa} S^*_{ab}\right), \tag{3}$$

where the Sa and Sb are the Cartesian components. Similarly, the rotationally averaged 3PA strength (in atomic units), $<\delta^{3PA}>$, for linearly polarized light with parallel polarization was be defined as[43,44]:

$$< \delta^{3PA} > = \frac{1}{35} \left(2 \sum_{a,b} S_{ab} S^*_{ab} + 3 \sum_{a,b} S_{aa} S^*_{ab}\right). \tag{4}$$

To estimate the impact of the solvent on the 2PA cross-sections, 2PA spectra in implicit ACN, dichloromethane (DCM) and dimethyl sulfoxide (DMSO) were calculated. The 2PA spectrum in PETA was calculated in Turbomole V7.4 (COSMO, epsilon of 2.2) due to the absence of the solvation model in Dalton 2020.0.

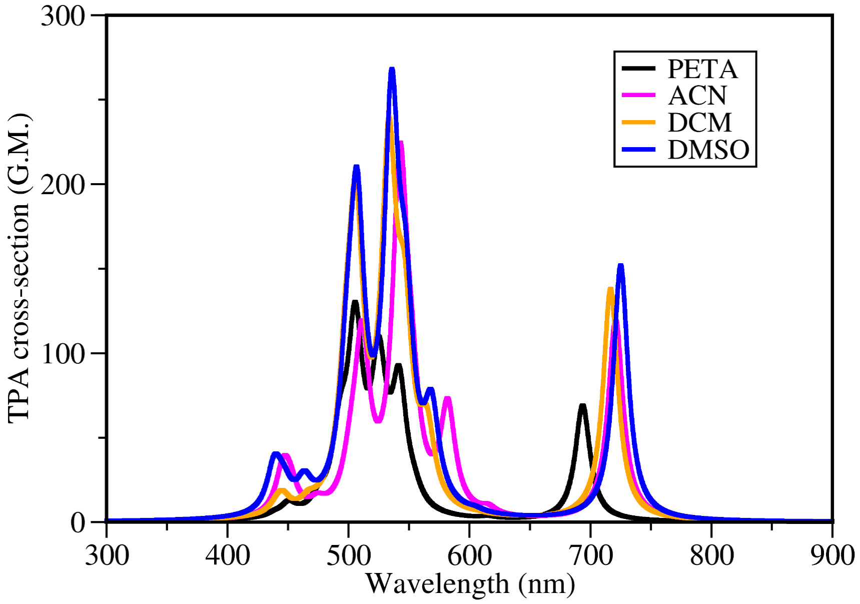

Fig. S8: Two-photon absorption (2PA) spectra of DETC obtained using TD-CAM-B3LYP/def2-TZVP level of theory in *implicit* ACN, DCM and DMSO (PCM model) performed in Dalton 2020.0. Spectrum in PETA (COSMO model) was performed in Turbomole V7.5. Spectra were plotted using half width at half maximum (HWHM) of 0.1 eV, Lorentzian type broadening function and considering a single laser beam as experimental set-up. Spectrum was plotted using half width at half maximum (HWHM) of 0.1 eV, Lorentzian type broadening function and considering single laser beam as experimental set-up. Spectrum in ACN is depicted in Fig. 2 in the main body.

Table S13: Values for one-photon absorption (1PA): excitation energy (E), wavelength (λ_{1PA}), oscillator strength (f), and two-photon absorption (2PA): wavelength (λ_{2PA}), 2PA strength (δ_{2PA}), cross section (σ_{2PA}) of DETC in *implicit* **ACN** (PCM model) computed in Dalton 2020.0. Data obtained using TD-CAM-B3LYP/def2-TZVP level of theory and optimized ground state geometry of DETC. No rescaling with regard to experimental data was applied. 1 GM corresponds to 1 × 10^{-50} cm^4 s $photon^{-1}$ $molecule^{-1}$

	**1PA**			**2PA**		
**Excitation**	**E (eV)**	**λ_{1PA} (nm)**	**f**	**λ_{2PA} (nm)**	**δ_{2PA} (a.u.)**	**σ_{2PA} (GM)**
1	3.44	360.42	0.97	720.84	1.38×10^4	119.00
2	4.08	307.65	0.05	615.31	3.25×10^2	3.96
3	4.33	291.04	0.03	582.08	4.48×10^3	61.60
4	4.55	276.13	0.02	552.27	3.09×10^3	46.90
5	4.65	271.30	0.14	542.60	1.25×10^4	198.00

6	4.91	255.64	0.07	511.27	$5.52x10^3$	97.60
7	4.99	250.98	0.07	501.96	$1.11x10^3$	20.30
8	5.28	237.06	0.02	474.13	$2.83x10^2$	5.80
9	5.55	225.84	0.10	451.67	$6.44x10^2$	14.60
10	5.61	222.99	0.05	445.99	$1.11x10^3$	25.60

Note: The 1PA spectrum computed with Dalton 2020.0 shows a slight difference with respect to the one computed with Gaussian16, which is reported in the main paper, i.e. the first excitation is at 360.42 nm and 359.30 nm using Dalton and Gaussian software, respectively.

Table S14: Values for one-photon absorption (1PA): excitation energy (E), wavelength (λ_{1PA}), oscillator strength (f), and three-photon absorption (3PA): wavelength (λ_{3PA}), strength (δ_{3PA}), cross section (σ_{3PA}) of DETC **in the gas phase** computed in Dalton 2020.0. Data obtained using TD-CAM-B3LYP/def2-TZVP level of theory and optimized ground state geometry of DETC in CAM-B3LYP-D3(BJ)/def2-TZVP in ACN.

	**1PA**			**3PA**		
**Excitation**	**E (eV)**	**λ_{1PA} (nm)**	**f**	**λ_{3PA} (nm)**	**$\delta_{3PA}x10^6$ (a.u.)**	**$\sigma_{3PA}x10^{-78}$ (cm^6 s^2 $photon^{-1}$)**
1	3.68	336.91	0.7617	1010.74	100	5.577
2	3.95	313.88	0.1303	941.65	8.85	0.628
3	4.54	273.09	0.0670	819.28	64.3	13.38
4	4.61	268.95	0.0269	806.84	63.7	1.098
5	4.78	259.38	0.0364	778.14	143	41.51
6	4.88	254.07	0.0333	762.20	100	5.771
7	5.07	244.54	0.0611	733.63	8.85	26.41
8	5.10	243.11	0.0227	729.32	64.3	81.69
9	5.34	232.18	0.0148	696.54	63.7	86.11
10	5.57	222.59	0.0184	667.78	143	1490

5. Optimization of excited states of DETC

Singlet excited (S_1) and triplet excited (T_1, T_3, T_4, T_6) states of DETC photoinitiator were fully optimized TD-CAM-B3LYP-D3(BJ)/def2-TZVP level of theory in ACN. (The optimization of the S_1 state using TD-B3LYP-D3(BJ)/def2-TZVP is discussed for comparison.) All optimized geometries were validated with vibrational analysis and molecular orbitals contributions. Comparison of the global minima structures in the ground state (S_0) and excited state potential energy surfaces is depicted in Fig. S9 and S11. All optimized structures, adiabatic energy differences and reorganization energies, corresponding to the geometric change of DETC in different states, were used to calculate nonadiabatic coupling matrix elements (NACME), spin-orbit coupling matrix elements (SOC) and photophysical rates as listed in Tables 1, S18-S20.

Figure S9 illustrates changes in the optimized S_0 and S_1 geometries when employing two different functionals. While the ground state structure is similar, the significant divergences are evident in the S_1 geometries obtained using B3LYP and CAM-B3LYP functionals (Fig. S9b). In the B3LYP-optimized S_1 geometry (cyan), a considerable relaxation is observed compared to the S_0 geometry (orange), resulting in a substantial side group twist of 97.7° with respect to the dye core (C9C8C11C17, Fig. S9d). In contrast, the twist in the S_0 geometry is 45.9°. Conversely, the S_1 geometry optimized with CAM-B3LYP only slightly differs from the S_0 geometry, exhibiting a twist of 29.5° (47.3° in S_0), as depicted in Fig. S9c. The significant relaxation observed in the B3LYP-optimized geometry, leading to a perpendicular orientation of the side group with respect to the core, is likely unphysical. Tozer et al.[46] proposed that a drop in the overlap of HOMO and LUMO orbitals in specific nuclear conformation space regions results in underestimated excitation energies, collapsing the excited state surface towards the ground state surface. This effect is particularly notable in cases where a side group experiences a substantial twist (~90°), leading to the formation of an intramolecular charge transfer (ICT) state. Authors have shown that this breakdown is significant with PBE and B3LYP functionals, while it is mitigated using a Coulomb-attenuated functional like CAM-B3LYP. Similar observation is found in the case of the DETC molecule studied here.

Additionally, the experimental Stoke shift of ~3710.2 cm^{-1} (see Table S3) for DETC indicates a charge transfer (CT) character for S_1, accurately described by CAM-B3LYP, rather than a twisted intramolecular charge transfer (TICT) state, which would yield a much higher shift. Previous research by Cao et al.[47] has demonstrated that a typical TICT emission in organic molecules, such as N,N-dimethyl-4-((2-methylquinolin-6-yl)ethynyl)aniline in DMSO, results in a mega-Stokes shift of ~210 nm. This substantial geometry relaxation primarily accounts for the pronounced fluorescent solvatochromism observed. The reported Stokes shift for DETC in experiment in ACN is ~80 nm (see Fig. S3 and Table S3), which is significantly lower than the mega-Stokes shift typical of a TICT state. Therefore, DETC does not possess the TICT that B3LYP suggests, demonstrating the B3LYP functional fails to accurately describe the nature of DETC. This observation emphasizes the necessity of employing the CAM-B3LYP functional instead of B3LYP, even if the 1PA spectra calculated differ from the experimentally measured spectra (see Fig. S2, S3).

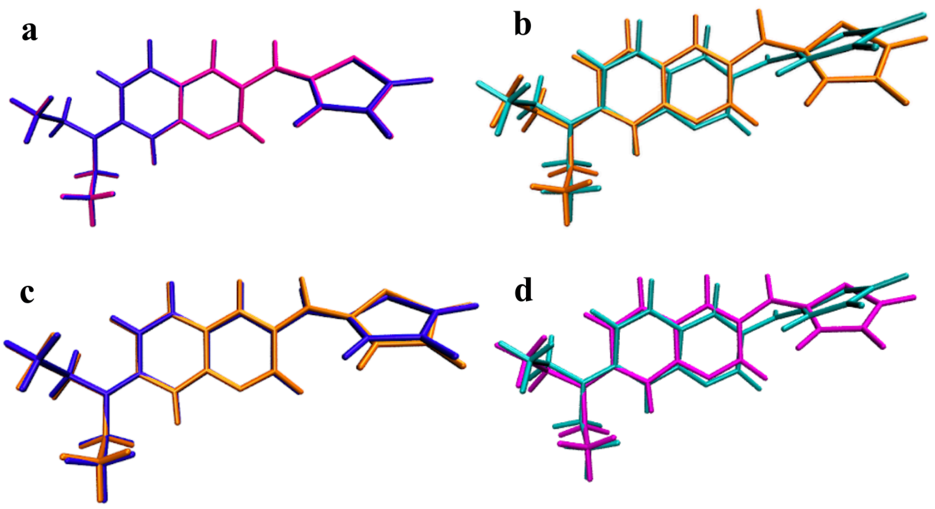

Fig. S9: Overlap of the optimized DETC structures obtained with different functionals: **a)** the optimized S_0 state with CAM-B3LYP (blue) and B3LYP (magenta), **b)** the optimized S_1 state with CAM-B3LYP (orange) and B3LYP (cyan), **c)** S_0 (blue) and S_1 (orange) geometries with CAM-B3LYP and **d)** S_0 (magenta) and S_1 (cyan) geometries with B3LYP.

The investigation of the bond length/order alternation (BLA/BOA) index, as well as the alteration of bond angle and dihedral for specific path of the optimized S_1 state of DETC in ACN with both B3LYP and CAM-B3LYP functional is reported in Table S15 and Fig. S10. The investigated chain contains atoms 3-5, 7-8, 11, 17-18 (see Fig. S1). The correspondence between bond, angle and dihedral index in the Fig. S10 with the atom labels in the molecular geometry are as follow:

Bond index: 1=[5-4], 2=[4-3], 3=[3-7], 4=[7-8], 5=[8-11], 6=[11-17], 7=[17-18]

Angle index: 3= [3, 7, 8], 4=[4, 3, 7], 5= [5, 4, 3], 7=[7, 8, 11], 8= [8, 11, 17], 9=[11, 17, 18]

Dihedral index: 3= [3, 7, 8,11], 4= [4, 3, 7, 8], 5=[5, 4, 3, 7], 7=[7, 8, 11, 17], 8=[8,11,17,18]

Table S15: Bonding relationship for the chain with the atom number 5-4-3-7-8-11-17-18 of the S_1 optimized state in CAM-B3LYP-D3(BJ)/def2-TZVP and B3LYP-D3-(BJ)/def2-TZVP. BLA and BOA stand for bond length alternation and bond order alternation, respectively. Results are displayed in ångström (Å).

	**CAM-B3LYP**	**B3LYP**
BLA	-0.0515	-0.1159
average length of even bonds	1.4317	1.3914
average length of odd bonds	1.4831	1.5072
BOA	-0.1170	0.0502

average bond order of even bonds	1.1908	1.2653
average bond order of odd bonds	1.3079	1.2152

Odd and even bonds are defined as bond number 1, 3, 5… and 2, 4, 6…, respectively, which are used for the calculation of the averaged values. The general definition is expressed as: [average bond length/order of even numbered keys] - [average bond length/order of odd number keys]. Since the atomic sequence from the beginning to the end of the chain is defined by atoms 5-4-3-7-8-11-17-18, then the BLA/BOA is [R(4-3)+R(7-8)+R(11-17)]/3 - [R(5-4)+R(3-7)+R(8-11)+R(17-18)]/4.

As reported in the Multiwfn manual[40], compared with BLA, which reflects the alternating characteristics of bonds at the geometric level, BOA reflects this from the level of electronic structure.

We see that both BLA and BOA differ between functionals. The CAM-B3LYP functional is more consistent in the calculation of bond lengths than the B3LYP functional, i.e. the change in the length of even and odd bonds is smaller.

From data reported in the Fig. S10, we can observe the capture of the torsion (dihedral angle with index "7", corresponding to the dihedral angle formed between atoms 7-8-11-17) of DETC using CAM-B3LYP. It is close to planar (~160 °), while the B3LYP functional results in a dihedral of 86°. As discussed above, it is not correlating with the Stoke shift observed in the experiment. Data related to valence angles computed with the two functionals show less significant differences.

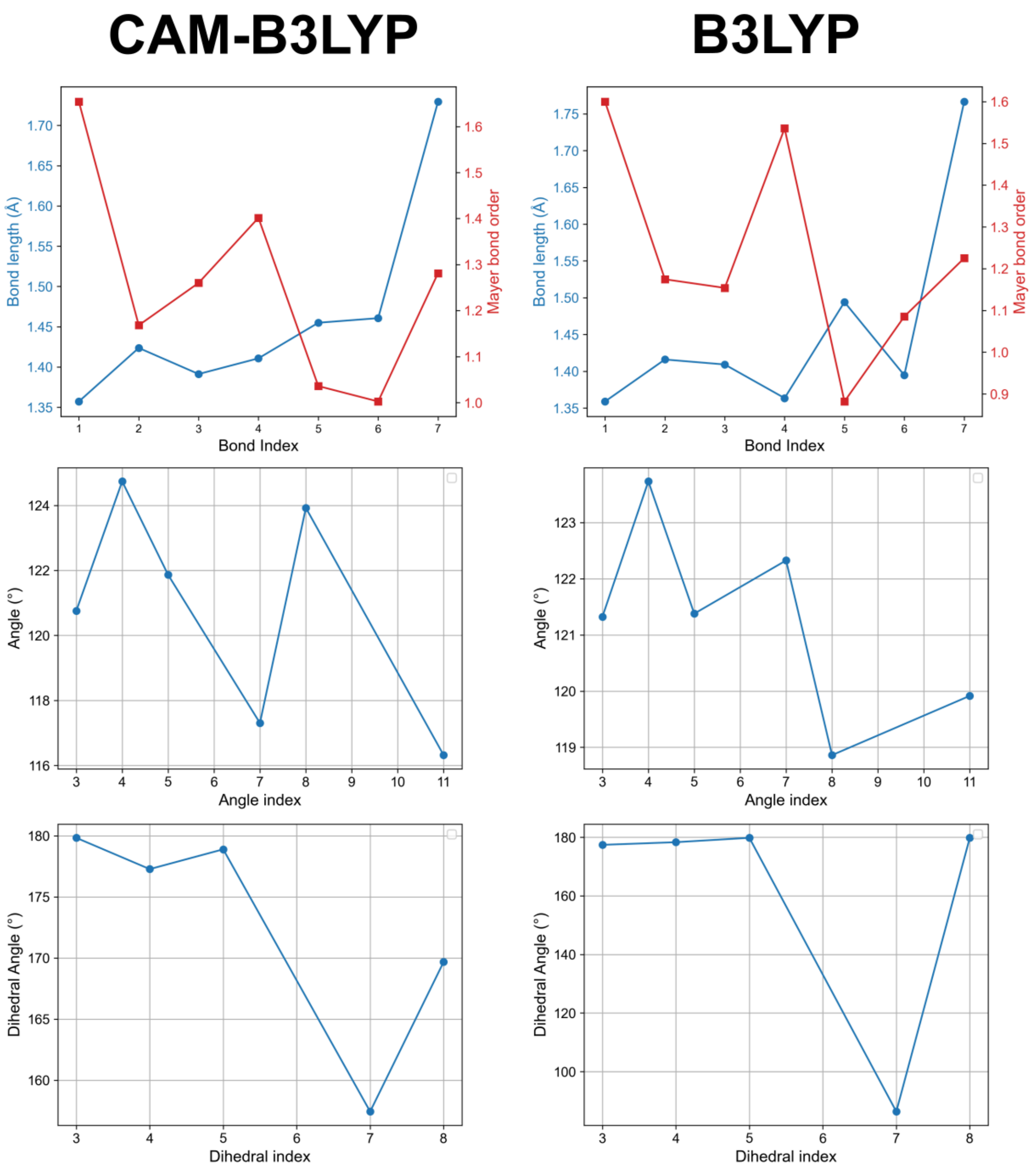

Fig. S10: Comparison of structural parameters of the S_1 optimized state obtained with the CAM-B3LYP and B3LYP functionals. Upper panel: representation of bond length of different DETC bonds (used for the BLA calculation) and bond order (used for BOA calculation). The values of valent and dihedral angles are shown in the middle and bottom panels, respectively.

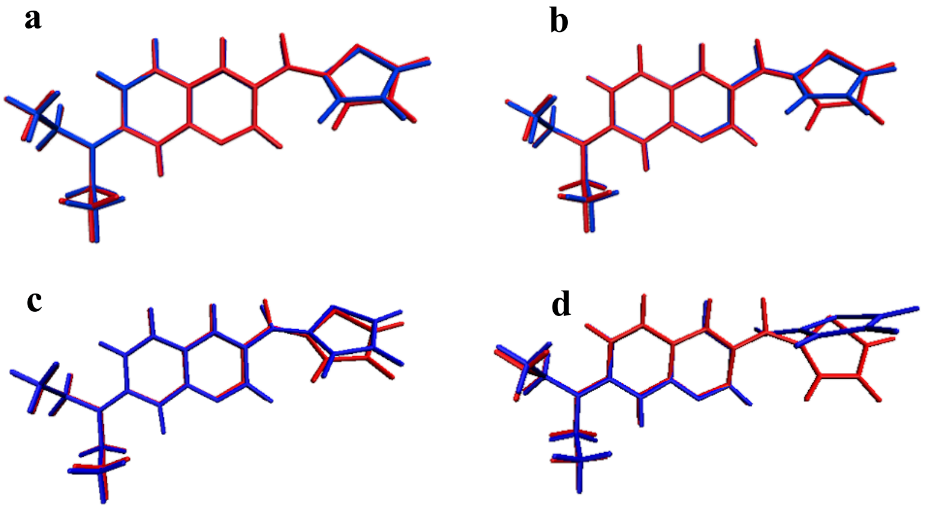

Fig. S11: Graphical comparison of S_0, S_1 and T_3 optimized geometries of DETC with CAM-B3LYP: **a)** ground state (in blue) to T_3 in gas-phase (in red) **b)** ground state in ACN (in blue) to T3 in gas-phase (in red) **c)** S_1 in ACN (in blue) to S_1 in gas-phase (in red) and **d)** T_3 in ACN (in blue) to T_3 in gas-phase (in red). Only slight changes have been observed for a), b) and c), while the relevant deformation of the dihedral $C_9C_8C_{11}C_{17}$ is observed for T_3 in ACN (see d) with respect to the T_3 in the gas-phase. Since we experience ISC and RISC convergence problems using T_3 optimized in ACN, we calculated ISC, RISC and IC rates also considering the T_3 state optimized in the gas phase.

Finally, we have used the S_1 geometry optimized with TD-CAM-B3LYP in ACN to compare the energy difference between the singlet and triplet states obtained in TD-DFT to wavefunction-based methods. Among them we have considered: coupled cluster (CC2), spin-component scaled CC2 (SCS-CC2), second order algebraic diagrammatic construction with SCS variant (SCS-ADC2), and scaled-opposite-spin CC2 (SOS-CC2) methods. All methods mentioned were applied in combination with the def2-TZVP basis set in *implicit* ACN. The vertical excitation energies computed by TDDFT-CAM-B3LYP (ACN) and all wavefunction-based methods are listed in Table S16-S17. The solvation effects were included by employing the COSMO model. In the case of SCS-ADC2, a corrected linear response was also applied. The latter were not computed for the other wavefunction-based methods, because they are not yet implemented in TURBOMOLE (up to V.7.7). To be consistent, we have also computed the singlet and triplet vertical excitation energies in TDDFT with the inclusion of the cLR effects (in Gaussian16).

Table S16: Vertical excitations obtained by SCS-CC2, SCS-ADC2, SOS-CC2, CC2 and CCS/def2-TZVP calculations in ACN (COSMO and corrected LR for SCS-ADC2) **on S_1 geometry obtained with TD-CAM-B3LYP in ACN**. Calculations were made in TURBOMOLE V.7.5.1. Results for TDDFT were obtained with CAM-B3LYP-D3(BJ)/def2-TZVP in ACN. Results are reported in eV. The energy differences are listed in Table S17.

	S_1	S_2	T_1	T_2	T_3	T_4	T_5	T_6	T_7
**DFT adiab.***	3.04	3.61	2.09	-	3.16	3.59	-	3.88	-
**DFT vert. from S1 cLR**	3.23	3.03	2.02	2.75	3.34	3.61	3.71	3.74	3.83
**SCS**	2.89	3.84	2.52	3.45	3.69	4.09	4.18	4.24	4.37
**SCS-ADC2**	2.78	3.70	2.49	3.40	3.58	4.02	4.12	4.19	4.32
**SCS-ADC2 cLR**	2.74	3.59	2.44	3.37	3.51	3.95	4.10	4.15	4.31
**SOS**	2.93	3.98	2.58	3.48	3.82	4.18	4.26	4.29	4.47
**CC2**	2.78	3.54	2.38	3.31	3.46	3.88	3.98	4.13	4.17

*cLR effects were not included in the optimization of excited states

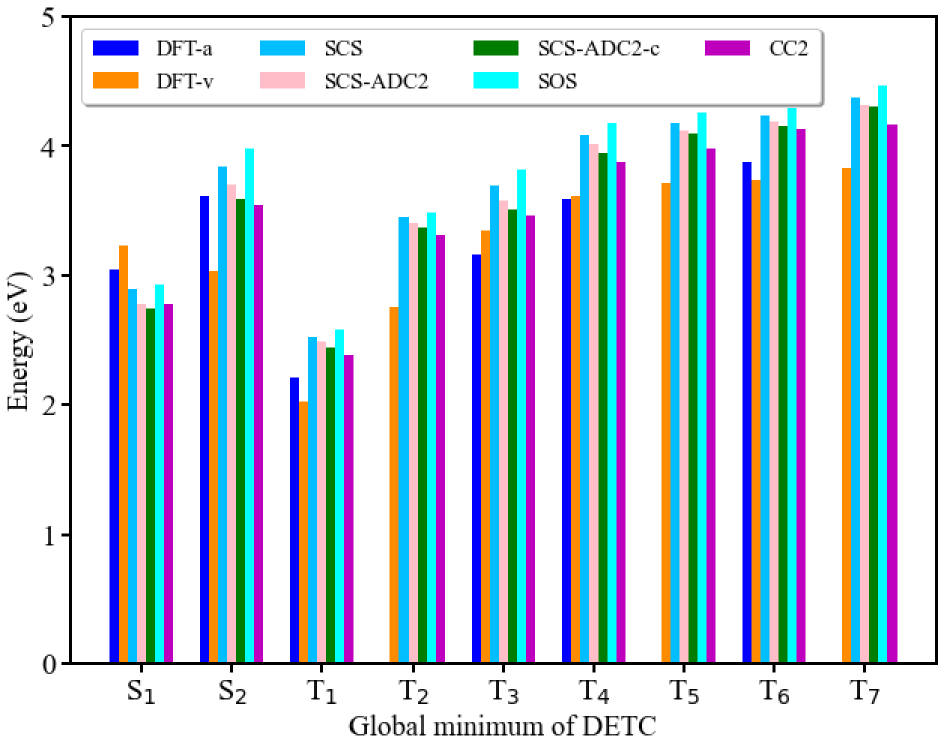

Fig. S12: Graphical visualization of the excitation energies, listed in Table S16 obtained with DFT adiabatic (DFT-a), DFT vertical (DFT-v), SCS-CC2 (SCS), SCS-ADC2 (SCS-ADC2), SCS-ADC2 corrected linear response (cLR) (SCS-ADC2-c), SOS-CC2 (SOS) and CC2 (CC2) with def2-TZVP basis set. All calculations were performed in ACN (COSMO and cLR for SCS-ADC2) on S1 geometry obtained with TD-CAM-B3LYP in ACN.

Table S17: Energy difference (ΔE) between singlet S_1 and triplet T_1-T_7 excitations computed on **S_1 geometry obtained with TD-CAM-B3LYP in ACN** with the approaches reported in Table S16. Results are reported in eV.

	**ΔE S_1-T_1**	**ΔE S_1-T_2**	**ΔE S_1-T_3**	**ΔE S_1-T_4**	**ΔE S_1-T_5**	**ΔE S_1-T_6**	**ΔE S_1-T_7**
**DFT adiab.***	0.95	-	-0.12	-0.55	-	-0.84	-
**DFT vert. from S1 cLR**	1.21	0.48	-0.11	-0.38	-0.48	-0.51	-0.60
**SCS**	0.37	-0.56	-0.80	-0.88	-0.91	-1.07	-1.15
**SCS-ADC2**	0.29	-0.62	-0.80	-1.24	-1.34	-1.41	-1.54

**SCS-ADC2 cLR**	0.30	-0.63	-0.77	-1.21	-1.36	-1.41	-1.57
**SOS**	0.35	-0.55	-0.89	-1.25	-1.33	-1.36	-1.54
**CC2**	0.40	-0.53	-0.68	-1.10	-1.20	-1.35	-1.39

*cLR effects were not included in the optimization of excited states

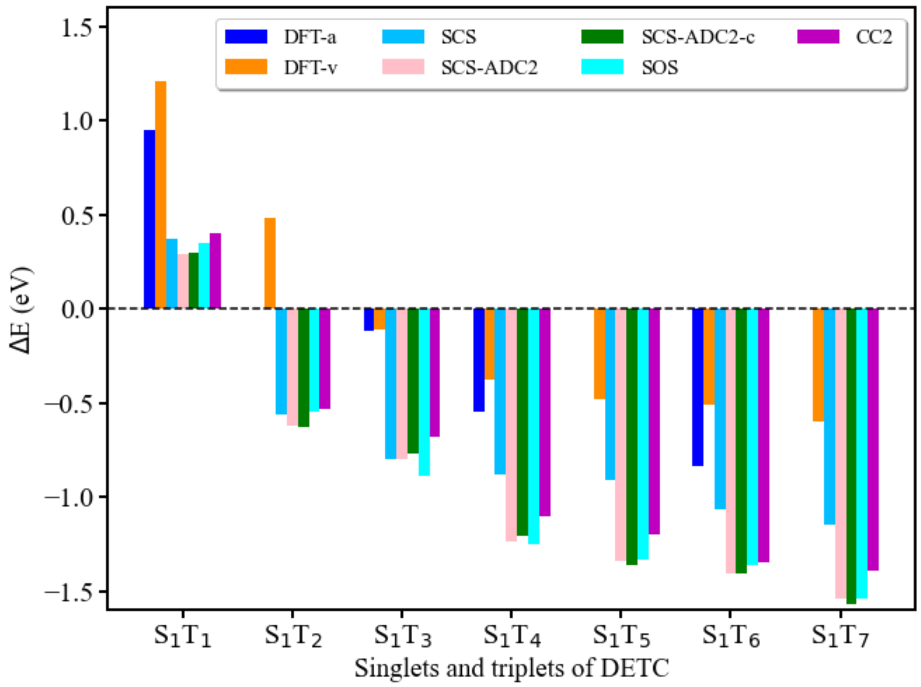

Fig. S13: Graphical visualization of the energy difference (ΔE) between the singlet S_1 and the triplet T_1-T_7 excitations of DETC computed on S_1 geometry obtained with TD-CAM-B3LYP in ACN with the approaches reported in Table S17.

Using the SCS approach, we can see that the T_2 state is more aligned to S_1 (ΔES_1-T_2=-0.56 eV) than T_3 (ΔES_1-T_3=-0.80 eV). On the other hand, with CAM-B3LYP and with the addition of cLR correction: ΔES_1-T_2 and ΔES_1-T_3 in ACN are 0.48 eV and -0.11 eV, respectively. Here, the position of T_2 state is more far away than the T_3 state. We see that TD-DFT data always indicate the T_3 state to be closer to S_1. However, vertical excitations in SCS-CC2 and all other wavefunction-based methods applied show that T_2 is closer to S_1 than T_3. Since the converged adiabatic T_2 state is lower than the T_1 state and ISC rates could not be converged for this state neither in ACN, nor in the gas phase, we cannot provide adiabatic energy differences. We assume the existence of subtle dynamical effects or coupling between T_1 and T_2 states (we clearly see that T_2 is close to T_1, see Fig. S12) that TDDFT cannot capture, which should be further analyzed by high level molecular dynamics simulations in excited states. The inclusion of the cLR effects, which are important for DETC (see Tables S6-S8) and has been performed by SCS-ADC2, shows a neglectable difference of ~0.01-0.03 eV with respect to COSMO. For this reason we can consider negligible the cLR effect on the results obtained with COSMO. The spin-component scaled versions of CC2 as SOS-CC2 and SCS-CC2 show deviations that rise up with high triplets i.e. ~0.39 eV as

maximum deviation for T_7 (see Table S17). The differences for SOS-CC2 and SCS-CC2 are slightly smaller than for unscaled CC2 and the accuracy is comparable[48], with the lowest deviations from the experimental values previously observed for SCS-CC2[26] for organic molecules. The ADC(2) method with the SOS variant showed results comparable to SOS-CC2[26,49].

6. Radiative and nonradiative rates

As previously reported[50], the electronically excited state is short-lived and may undergo deactivation through a number of chemical and physical processes that result in either emission of light (luminescence) or nonradiative conversion (IC, ISC) of energy into heat. Employing the Fermi Golden Rule (FGR) and second-order perturbation theory, the nonradiative decay rate constant can be defined as[51]:

$$K_{nr} = \frac{2\pi}{\hbar}\sum_{\nu_i\nu_f} P_{i\nu i}(T)\left|\hat{H}'_{\nu f,\nu i} + \sum_{\nu_j}\frac{H'_{\nu f,\nu j}H'_{\nu j,\nu i}}{E_{i\nu i}-E_{f\nu f}}\right|^2 \delta\times(E_{i\nu i} - E_{f\nu f}) \quad (5)$$

where ħ is the reduced Planck's constant, v_i, v_f, *and* v_j are the vibrational quanta of the initial, final and intermediate states, $P_{i\nu i}(T)$ is the Boltzmann distribution of the vibrational manifolds in the initial state, and $\hat{H}'$ is the sum of the non-adiabatic coupling ($\hat{H}^{BO}$) and spin-orbit coupling ($\hat{H}^{SO}$) operators. When the two states are in the same spin manifold, only the first-order perturbation is considered and the spin-orbit coupling can be neglected. The internal conversion (IC) rate constant becomes:

$$k_{IC} = \frac{2\pi}{\hbar}\sum_{\nu_i\nu_f} \hat{P}_{i\upsilon_i}(T)\left|\sum_k < \Phi_f\left|\hat{P}_k\right|\Phi_i >< \Theta_{f\upsilon_f}\left|\hat{P}_k\right|\Theta_{i\upsilon_i} >\right|^2 \delta\,(E_{i\upsilon_i} - E_{f\upsilon_f}) \quad (6)$$

where $\hat{P}_k$ is the nuclear momentum operator for the k_{th} mode and Φ and Θ are the electronic and vibrational state vectors, respectively. Applying the Fourier transformation to equation (6), IC rate is calculated as:

$$k_{IC} = \frac{1}{\hbar^2}\int dt e^{i\omega_{if}t}\sum_{k,l} Z_i^{-1} < \Theta_f\left|\hat{P}_k\right|\Theta_i >< \Phi_i\left|\hat{P}_l\right|\Phi_f > \quad \times Tr\left[\hat{P}_k e^{-i\tau_f\hat{H}_f}\hat{P}_l e^{-i\tau_i\hat{H}_i}\right] \quad (7)$$

where Z_i^{-1} is the partition function of the initial state, $\tau_i = -i\beta - t/\hbar$, $\tau_f = t/\hbar$, and $\hat{H}_i$ and $\hat{H}_f$ are the harmonic oscillator Hamiltonians of the initial and final states, respectively. The DFT/TDDFT approach can be used for solving the thermal vibration correlation function (TVCF)[52] in equation (7).

The intersystem crossing (ISC) rate constant between two electronic states with different spin multiplicities can be written as:

$$k_{ISC} = \frac{1}{\hbar^2}\langle\Phi_f|\hat{H}^{SO}|\Phi_i\rangle^2 \int dt\, e^{i\omega_{if}t}\, Z_i^{-1}\rho_{ISC}(t,T) \quad (8)$$

where $\rho_{ISC}(t,T)$ is the thermal-vibration correlation function. For nonradiative ISC between different spin states (eq. 4) can be expanded into three terms, corresponding to $\hat{H}^{SO}$ only, the

product of $\hat{H}^{SO}$ and $\hat{H}^{BO}$, and quadratic in $\hat{H}^{BO}$. Each term can be written as a time integration of vibrational correlation functions such as:

$$Tr\left[e^{-i\tau_f \hat{H}_f} e^{-i\tau_i \hat{H}_i}\right], Tr\left[\hat{P}_k e^{-i\tau_f \hat{H}_f} e^{-i\tau_i \hat{H}_i}\right] \text{or } Tr\left[\hat{P}_k e^{-i\tau_f \hat{H}_f} \hat{P}_l e^{-i\tau_i \hat{H}_i}\right] \tag{9}$$

multiplied by the appropriate prefactors from the corresponding electronic couplings[4].

Nonradiative decay rates (IC, ISC and RISC), according to equations (7) and (8), in ACN, were calculated using MOMAP Version 2022A (2.3.3) (Materials Property Prediction Package) based on optimized DETC structures considering the integral interval of the correlation function, i.e., t_{max} of 1000 fs combined with the integration timestep of the correlation function, i.e., dt of 0.01 fs neglecting the Duschinsky rotation. ΔE_{SS} and ΔE_{ST} were calculated as the adiabatic energy difference between optimized singlets and corresponding T_x excited state. ΔE_{SS} and ΔE_{ST} of DETC in ACN (*implicit* solvent model) are depicted in Fig. 4. The adiabatic energy gap was computed with Gaussian16 Rev.C.01. Nonadiabatic coupling elements, i.e. NACME, between singlet excited states and spin-orbit coupling i.e. SOC between singlet and triplet excited states were calculated at TDDFT level using Gaussian16 Rev.C.01 and ADF 2020.1, respectively utilizing the optimized DETC structures. In addition, SOC were computed taking into account relativistic effects (scalar) with ZORA[53–55] formalism (Zero Order Regular Approximated Hamiltonian), in ADF. The COSMO solvation (ACN) was used for SOC. Moreover, the effect of non-equilibrium solvation was applied, i.e. that the dielectric constant of the excited state is different from the ground state dielectric constant. Calculations with the triple-zeta basis set (TZP) have been performed. All SOC values were calculated using the optimized singlet and triplet excited states, and are reported in Tables 1, S18, S19. SOC for the non-radiative decay rate of T_1 (ISC T_1-S_0) was computed in ORCA Version 4.1, employing the conductor-like polarizable continuum model (CPCM) for ACN using quasi-degenerate perturbation theory, CAM-B3LYP functional and def2-TZVP basis set.

For radiative processes (fluorescence and phosphorescence) analogous vibrational correlation function formalisms, as in equations (6) and (7) were applied[37]. In this case, the electronic coupling term becomes the electric dipole moment μ. For the emission spectrum as an example, FGR gives:

$$\sigma_{em}(\omega) = \frac{4\omega^3}{3c^3\hbar} \sum_{\nu_i \nu_f} \hat{P}_{i\upsilon_i} |< \Theta_{f\upsilon_f} | \hat{\mu}_{fi} | \Theta_{i\upsilon_i} > |^2 \delta(\omega_{i\upsilon_i, f\upsilon_f} - \omega) \tag{10}$$

where ω is the frequency, c is the speed of light, ν_i *and* ν_f are the vibrational quanta of the initial and final state, Θ is the vibrational state vector associated to initial and final state, $\hat{\mu}_{fi}$ is the transition dipole moment between initial and final state. Equation (10) can be expressed as the transition dipole multiplied by a time integration over the correlation function. The radiative decay rate constant is therefore the integration over the emission spectrum:

$$k_r = \int_0^\infty \sigma_{em}(\omega) d\omega \tag{11}$$

The calculated radiative decay rates in MOMAP (fluorescence, phosphorescence), according to equations (10) and (11) are listed in Tables 1 and S20. Adiabatic Hessian (AH) PES model within TD-DFT was used for the computation of all the radiative rates. For further information about the methodology and approach used in this work, one could refer to the corresponding references[2,56].

Table S18: Spin-Orbit Coupling (SOC) between singlet and triplet states of DETC computed using CAM-B3LYP. The SOC value (using the singlet optimized geometry) was used to calculate ISC rate, while the RSOC value (using the triplet optimized geometry) was used for the RISC rate (see Table S19). All values were computed using ADF software. SOC in implicit ACN with the inclusion of non-equilibrium effects for the solvent are colored in blue, while values in implicit ACN and in the gas-phase are in purple and orange, respectively.

Transition	SOC (cm^{-1})	RSOC (cm^{-1})
S_1-T_1	0.97<br>0.67<br>5.62	0.80<br>0.56<br>4.93
S_1-T_2	3.34<br>3.00<br>8.63	2.87<br>2.70<br>6.68
S_1-T_3	7.55<br>8.16<br>11.85	5.47<br>5.77<br>19.04
S_1-T_4	4.49<br>4.52<br>7.54	7.68<br>11.00<br>2.01
S_1-T_6	9.51<br>9.65<br>7.41	2.09<br>2.09<br>3.13
S_2-T_1	22.96<br>22.03<br>37.71	15.52<br>14.51<br>20.58
S_2-T_2	21.20<br>19.93<br>7.32	23.68<br>22.57<br>25.89
S_2-T_3	26.96<br>27.56<br>31.31	6.41<br>5.63<br>7.56
S_2-T_4	25.81<br>24.96<br>22.88	1.49<br>2.97<br>1.45

**S_2-T_6**	9.43<br>9.77<br>3.06	2.35<br>1.97<br>5.45

Table S19a: Intersystem crossing (ISC) and reverse ISC (RISC) for S_1-T_n rates of DETC in implicit ACN calculated with TD-CAM-B3LYP-D3(BJ)/def2-TZVP level of theory. ISC and RISC rates (in s^{-1}) were calculated using SOC (in cm^{-1}) and RSOC (in cm^{-1}) in ACN with the inclusion of non-equilibrium effects (see Table S18) and reorganization energy from singlet to triplet (λ ST, in eV) and from triplet to singlet (λ TS, in eV) using an approach described above and in Methods. The adiabatic energy gap (in eV) between singlets and triplets was computed starting from the optimized singlet and triplet geometry, respectively. Rates were computed considering the integration time step (dt) of 0.01 fs without the inclusion of Duschinsky rotation. In comparison to the data in the main body, the zero point energy correction (ZPE) is not included here. The deviation in the rate values with regard to the inclusion of ZPE is below 19%.

	**ISC**	**RISC**	**ΔE**	**SOC**	**RSOC**	**λ ST**<br>**λ TS**
**S_1-T_3#**	$3.14x10^{6}$	$8.46x10^{7}$	-0.11	7.55	5.47	1.195<br>0.763
**S_1-T_3***	$6.09x10^{8}$	$1.08x10^{8}$	0.36	11.85	19.04	0.072<br>0.114
**S_1-T_3****	$1.26x10^{10}$	$7.02x10^{8}$		7.55	5.47	0.316<br>0.284
**S_1-T_3*****	$3.10x10^{10}$	$8.50x10^{8}$		11.85	19.04	
**S_1-T_3°°**	$5.15x10^{8}$	$6.46x10^{10}$	-0.11	11.85	19.04	0.284
**S_2-T_3#**	$1.59x10^{7}$	$2.23x10^{6}$	0.46	26.96	6.41	1.438
**S_2-T_3°°**	$2.04x10^{11}$	$7.15x10^{6}$	0.46	31.31	7.56	0.384

#S ACN , T ACN, SOC in ACN, ΔE in ACN; the distribution of time (in fs) vs real part (TVCF_RE) of correlation function is not converged (see Fig. S16)

*S gas , T_3 gas-phase, SOC in gas-phase, ΔE in gas-phase

** S ACN, T_3 gas-phase, SOC in ACN, ΔE in gas-phase

*** S ACN, T_3 gas-phase, SOC in gas-phase, ΔE in gas-phase

°°S ACN, T_3 gas-phase, SOC in gas-phase, ΔE in ACN

Table S19b: Internal conversion (IC) rates (in s^{-1}) of DETC in implicit ACN calculated with TD-CAM-B3LYP-D3(BJ)/def2-TZVP level of theory. IC rates (in s^{-1}) were calculated using Non-Adiabatic-Coupling-Matrix-Elements (NACME, in au) and reorganization energy (λ, in eV) using the approach described above and in Methods. The adiabatic energy gap (in eV) between triplets was computed starting from the optimized geometries. Rates were computed considering the integration time step (dt) of 0.01 fs without the inclusion of Duschinsky rotation. In comparison to the data in the main body, the zero point energy correction (ZPE) is not included here. T_1 is optimized with an unrestricted approach. The deviation in the rate values with regard to the inclusion of ZPE is below 3%.

<table>
<tr><th></th><th>IC</th><th>ΔE</th><th>NACME</th><th>λ T_1-T_3
λ T_3-T_1</th></tr>
<tr><td>T_3-T_1#</td><td>1.20×10^{6}</td><td>0.94</td><td rowspan="4">0.003</td><td>1.184
0.841</td></tr>
<tr><td>T_3-T_1*</td><td>2.91×10^{11}</td><td>0.72</td><td>0.448
0.476</td></tr>
<tr><td>T_3-T_1**</td><td>2.89×10^{11}</td><td>0.72</td><td>0.525
0.572</td></tr>
<tr><td>T_3-T_1°</td><td>2.10×10^{11}</td><td>0.94</td><td>0.448
0.476</td></tr>
</table>

#T_1 ACN, T_3 ACN, ΔE in ACN; the distribution of time (in fs) vs real part (TVCF_RE) of correlation function is not converged (see Fig. S15)
*T_1 gas, T_3 gas, ΔE in gas
** T_1 ACN, T_3 gas, ΔE in gas
°T_1 gas, T_3 gas, ΔE in ACN

Table S20: Rates of internal conversion (IC), fluorescence (fluo), respective lifetimes (in ns) computed with MOMAP using t of 1000 fs, dt of 0.01 fs without Duschinsky rotation.

	**IC 1/s**	**Fluo 1/s**	**IC lifetime ns**	**fluo lifetime ns**
**B3LYP**	1.21×10^{7}	1.00×10^{6}	82.34	1000.27
**CAM-B3LYP**	1.60×10^{8}	4.30×10^{8}	6.25	2.33

Lifetime of the S1 state in exp. is ~1 ns[4] and 0.25-0.44 ns[57](in toluene and methanol)

Fluorescence of DETC is better described by CAM-B3LYP functional than with the B3LYP, which results in emission rate of 4.30×10^{8} s^{-1} and IC of 1.60×10^{8} . This is consistent with reported experiments, where the lifetime of S_1 state was found to be of ~1 ns[4] and 0.25-0.44 ns (with the rate of ~2-4x10^{9} s^{-1}) in toluene and methanol[57]. Since the B3LYP functional does not properly describe the mixed charge transfer character of the relaxed S_1 excited state of DETC and the S_1 state differs significantly (see Fig. S9), it underperforms CAM-B3LYP.

To validate the correctness of the IC rates calculated by MOMAP, the evolution of the IC rates as a function of the adiabatic energies, i.e. the distribution of time vs real part of correlation function and the IC rate (k_{ic}) in Y in log scale versus adiabatic energy (energy in

Hartree) in X in linear scale, were plotted in Fig. S14 and S15). All plots were calculated employing the adiabatic Hessian (AH) model in MOMAP. The Adiabatic Shift (AS) model, defined as a ground state Hessian on the optimized S_1 geometry, gave similar results.

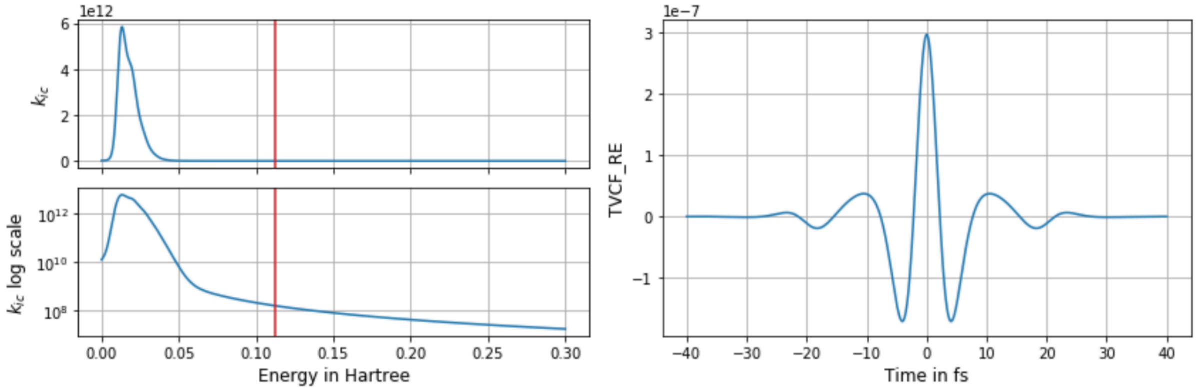

Fig. S14: Comparison of the evolution of the IC rates related to the transition $S_1 \rightarrow S_0$ as a function of the adiabatic energies, i.e. IC rate (k_ic) in Y in log scale versus adiabatic energy (Energy in Hartree) in X in linear scale (left) and the distribution of time vs real part of the correlation function (right). Adiabatic energy for S_1-S_0 is shown as a red straight line. Plots were computed with CAM-B3LYP in *implicit* ACN.

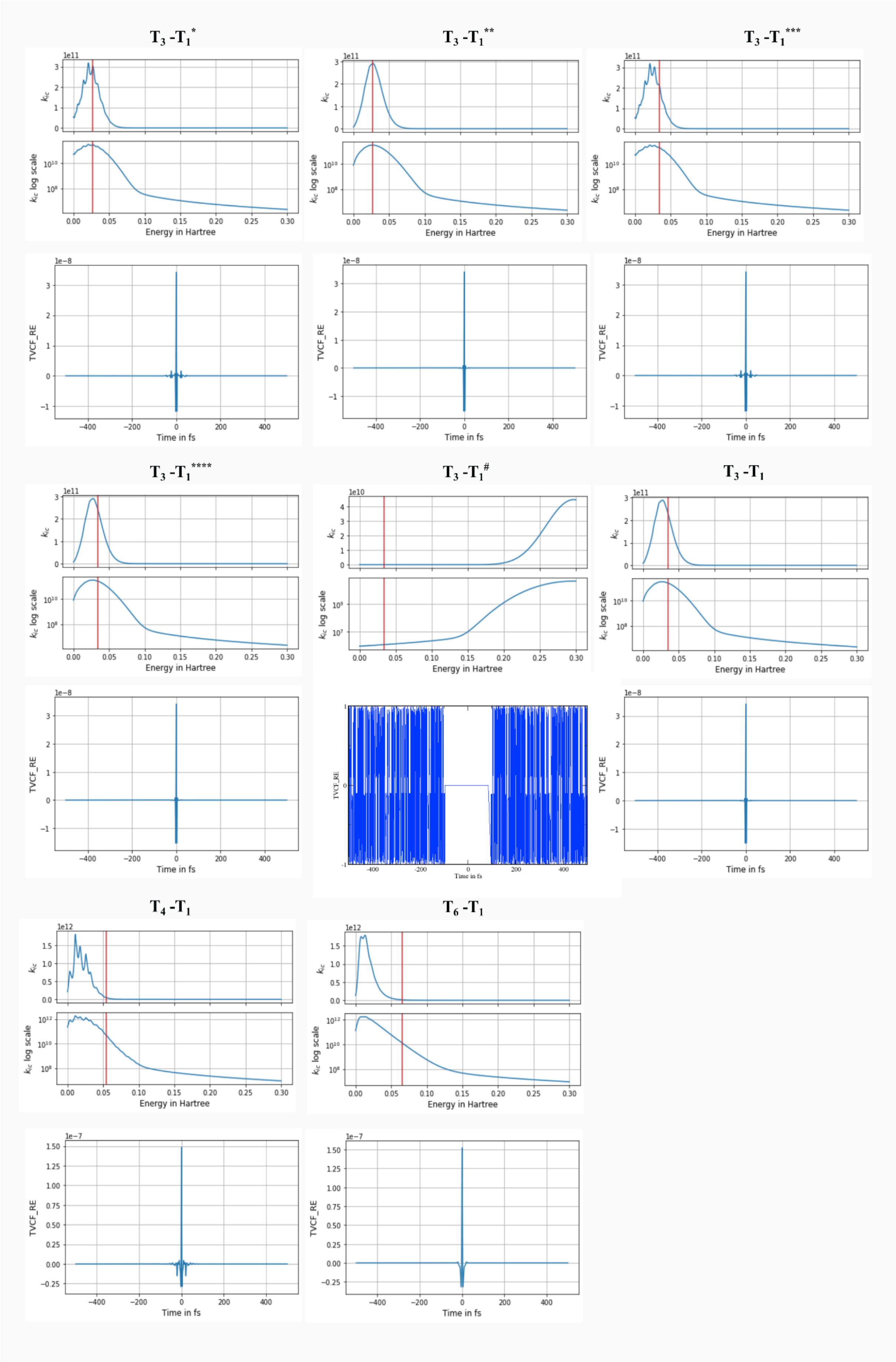

T3 -T1*
T3 -T1**
T3 -T1***
T3 -T1****
T3 -T1#
T3 -T1
T4 -T1
T6 -T1
kic
kic log scale
Energy in Hartree
TVCF_RE
Time in fs

Fig. S15 (cont.): Comparison of the evolution of the **IC rates** related to the transition $T_n \rightarrow T_1$ as a function of the adiabatic energies, i.e. IC rate (k_ic) in Y in log scale versus adiabatic energy (Energy in Hartree) in X in linear scale (up) and the distribution of time vs real part of correlation function (down). Adiabatic energy for T_n-T_1 is shown as a red straight line. Plots were computed with CAM-B3LYP in *implicit* ACN and in the gas-phase. The distribution of time (in fs) vs real part (TVCF_RE) of correlation function is not converged for $T_3 \rightarrow T_1^{\#}$. Plots for $T_2 \rightarrow T_1$ and $T_5 \rightarrow T_1$ are not available due to the convergence issues related to the TDDFT optimization of these excited states using CAM-B3LYP.

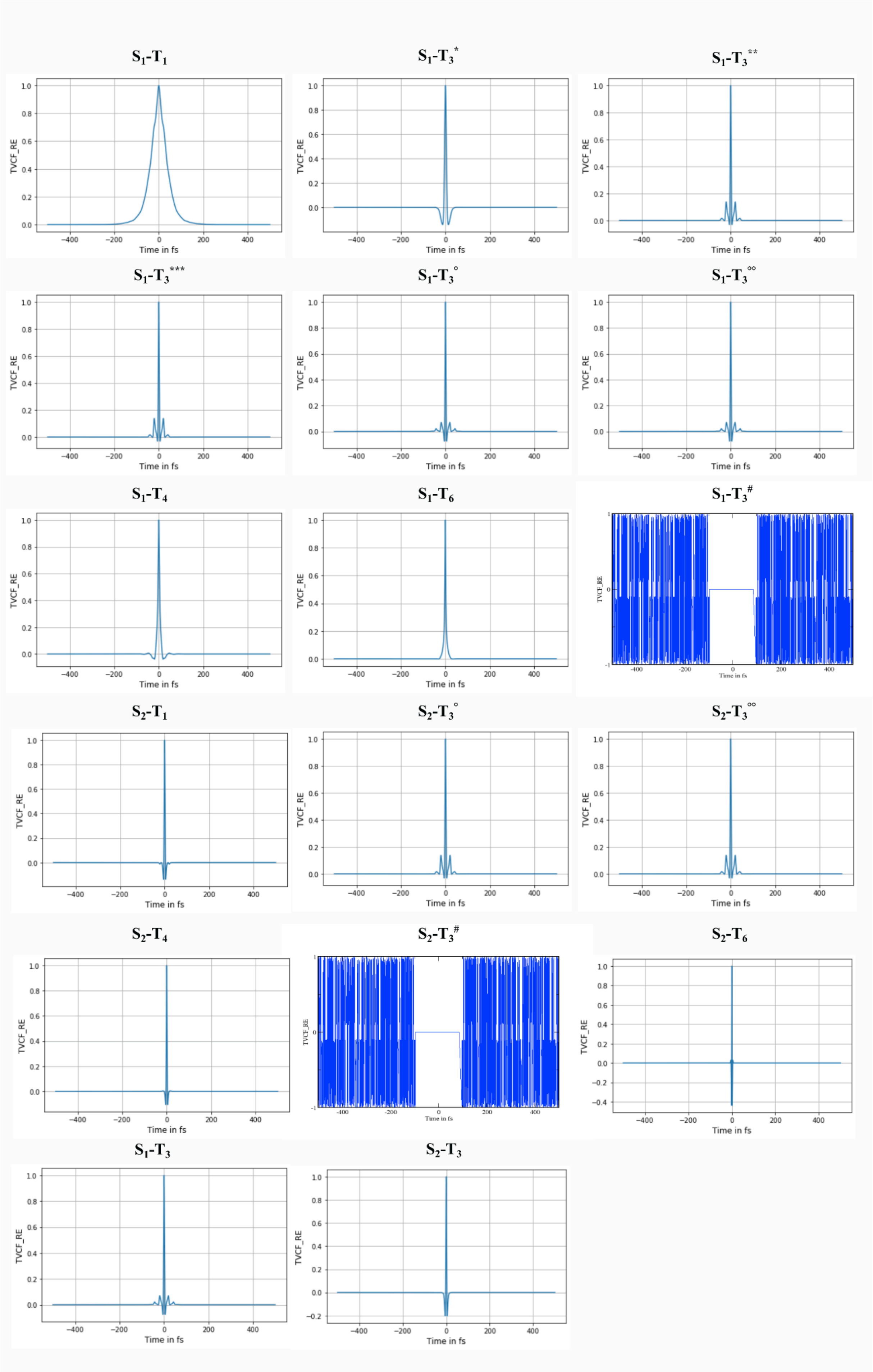

S1-T1
S1-T3*
S1-T3**
S1-T3***
S1-T3°
S1-T3°°
S1-T4
S1-T6
S1-T3#
S2-T1
S2-T3°
S2-T3°°
S2-T4
S2-T3#
S2-T6
S1-T3
S2-T3
TVCF_RE
Time in fs

Fig. S16 (cont.): Comparison of the distribution of time (Time in fs) vs real part (TVCF_RE) of correlation function for **ISC rates** reported in the paper. Plots are computed with CAM-B3LYP in *implicit* ACN and in the gas-phase. The distribution of time (time in fs) vs real part (TVCF_RE) of correlation function is not converged for $S_1 \rightarrow T_3^{\#}$ and $S_2 \rightarrow T_3^{\#\#}$ and therefore plots are not reported. Plots for $S_n \rightarrow T_2$ and $S_n \rightarrow T_5$ are not available due to the convergence issues related to the TDDFT optimization of these excited states using CAM-B3LYP.

In our study, we encountered challenges while investigating the correlation function for $S_1 \rightarrow T_3^{\#}$ and $S_2 \rightarrow T_3^{\#}$ ISC rates, as illustrated in Table S19 and Fig. S16. Unfortunately, the distribution of time versus the real part of the correlation function (Time in fs vs TVCF_RE) did not converge, raising concerns about the reliability of the computed rates in MOMAP, as indicated in Fig. S16. This lack of convergence might be attributed to high reorganization energies between S_1 or S_2 and T_3 states, as noted in Table S19, coupled with the presence of a twist of the thiophenyl group of DETC with respect ot the dye core in the optimized geometry of state T_3 in ACN (see Fig. S11), which is unphysical and extensively discussed in the main paper. Additionally, the IC rate for $T_3 \rightarrow T_1$ (considering optimized structures in ACN) did not converge either, as shown in Fig. S15. Attempts were made on IC and ISC rates involving T_3 using different methods (AS and VH), computing the Hessian according to the chosen approach and starting from various geometries (T_3, S_1, and T_1). However, the presence of multiple negative eigenvalues in the respective state Hessian suggested that this approach might not be meaningful. This indicates that the potential energy surfaces at the geometries investigated significantly differ in shape, making it challenging to compute rates using MOMAP. To address these issues, we opted to use the T_3 state computed with the CAM-B3LYP functional in the gas-phase. This state exhibited the correct CT character for the transition, similar to the results obtained in ACN (as shown in Fig. S6). The electron density difference in this state is similar to the T_3 in SCS-CC2. Importantly, the optimized geometry of this state did not display a twist in the thiophenyl side group of DETC with respect to the dye core, as illustrated in Fig. S11. Therefore, we considered the converged T_3 state in the gas phase for the ISC, RISC and IC rates calculation.

We combined this state with the S_1 state optimized in both the gas phase and ACN, and the S_2 state optimized in ACN (S_2 in gas-phase did not converge). Additionally, we incorporated SOC and energy gap (ΔE) values computed in both gas phase and ACN for comparison. Utilizing this approach, all rates calculated using gas-phase T_3 state converged successfully. Among them, the rate calculated by combining S_1 and S_2 optimized in ACN and T_3 optimized in gas-phase with the SOC values calculated in ACN and ΔE in ACN was selected to maintain the highest possible coherence of the parameters used for rate calculation, as detailed in the main paper.

Plots for the transition involving T_2 and T_5 were not reported and rates were not computed due to the convergence issues related to the TDDFT optimization of these states encountered both in the gas-phase and ACN.

7. Triplet-triplet absorption spectra

Table S21: Triplet-Triplet vertical excitation energies (in eV and nm) of DETC **in ACN** starting from optimized T_1 geometry. TA excitations were computed using (U)CAM-B3LYP-D3(BJ)/def2-TZVP level of theory. Transitions labeled "exc 1" to "exc 10" are not directly related to triplet Excited States numbering used in Table S1. They are described with the mixed molecular orbitals contributions, therefore can be assigned to the specific triplet state after additional analysis (see Fig. S6). Spin contamination is negligible, i.e. lower than 10%[58] of the value of $<S^2>$, i.e. s(s+1), generally accepted for organic molecules. TD-DFT results are qualitatively comparable to evGW-BSE+CD data in Table S22.

TA excitation	Energy (eV)	Wavelength (nm)	Oscillator strength
exc 1	1.52	815.20	0.1778
exc 2	1.75	705.75	0.1182
exc 3	1.82	679.83	0.1858
exc 4	2.26	548.21	0.0098
exc 5	2.38	520.97	0.0023
exc 6	2.47	501.85	0.0231
exc 7	2.56	483.20	0.4869
exc 8	3.06	405.21	0.0172
exc 9	3.24	382.50	0.0022
exc 10	3.35	369.22	0.0026

Table S22: GW-BSE triplet vertical excitation energies (in eV and nm) of DETC **in ACN** starting from optimized T_1 geometry. Excitations were computed in TURBOMOLE V7.4 using eigenvalue-only self-consistent GW (evGW) calculations, employing the contour deformation (CD) variant with CAM-B3LYP functional, def2-TZVP basis set and def2-TZVP auxiliary basis set. RI approximation for the Coulomb term was also included. Transitions labeled “exc 1” to “exc 10” are not directly related to triplet Excited States numbering used in Table S1.

	**Triplet-triplet excitations (evGW-BSE+CD)**		
	**ACN**		
**TA excitation**	**Energy (eV)**	**Wavelength (nm)**	**Oscillator strength**
exc 1	1.39	888.04	0.058
exc 2	1.50	825.79	0.020
exc 3	1.63	758.11	0.290
exc 4	2.07	598.73	0.012
exc 5	2.22	556.71	0.002
exc 6	2.33	530.44	0.028
exc 7	2.41	512.96	0.411
exc 8	2.85	434.22	0.027
exc 9	3.04	407.75	0.001
exc 10	3.17	390.34	0.002

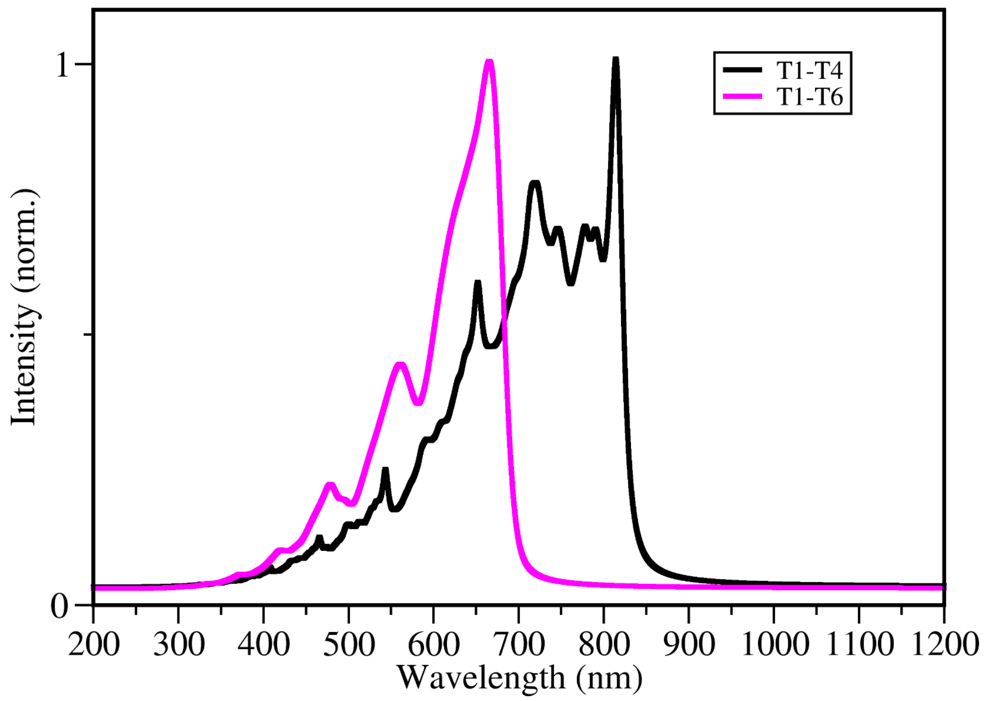

Fig. S17: Vibrationally resolved triplet-triplet spectra for computed at TDDFT level of theory within the Franck-Condon (FC) approximation at 100K with Adiabatic Hessian (AH-G16) as PES model. Spectra were plotted with HWHM of 80 cm^{-1} (i.e. 0.01 eV), convergence factor of $1.0x10^{-4}$ and Lorentzian type broadening function. Spectra were computed in *implicit* ACN using Dynavib with CAM-B3LYP-D3(BJ)/def2-TZVP level of theory. Due to significant geometry differences between T_1 and T_3 states in ACN (see Fig. S11), the vibronically resolved spectrum for the transition $T_1 \rightarrow T_3$ could not be generated. Spectra for $T_1 \rightarrow T_2$ and $T_1 \rightarrow T_5$ are not available due to TD-DFT convergence problems during the optimization of the related excited states.

Triplet-Triplet vibrationally resolved spectra using CAM-B3LYP-(BJ)/def2-TZVP show that $T_1 \rightarrow T_4$ transition happens at around ~800 nm, while $T_1 \rightarrow T_6$ transition occurs in the range of ~600-700 nm i.e. blue shifted with respect to the experimental employed wavelengths of femtosecond lasers of ~800 nm.

8. Reactivity of DETC with a co-initiator and PETA

DETC is a ketocoumarin, which should behave as Norrish Type II PIs (Fig. 1c) and react with co-initiators being in T_1 state. To understand mechanisms of the reaction in Fig. 1f, DBA co-initiator was used as a model amine-based co-initiator, used in 3D printing[59] (see Fig. 3). Since T_1 triplet state of Norrish Type II PIs is hypothesized to be active in the polymerization activation[60,61] without co-initiators, reaction between DETC and PETA was calculated for clarity. Homolytic cleavage of C-H, C-C, and C-N bonds of PETA, DBA and DETC at different electronic states (ground, singlet and triplet excited) was estimated through Bond Dissociation Enthalpies (BDE)[62] as well as the Gibbs free energy calculation according to equation (12) and (13):

$$BDE_{(A-B)} = \Delta H_{AB} - (\Delta H_A + \Delta H_B), \quad (12)$$

where AB is the bond broken and formed in the chemical reaction, ΔH_A and ΔH_B are enthalpy values of respective optimized radicals. As such, BDE is a cumulative difference between BDE values of all bonds broken and formed for reactants and products in a chemical reaction.

$$\Delta G = \Sigma(G_{products} - G_{reagents}), \quad (13)$$

where $G_{products}$ and $G_{reagents}$ denote Gibbs free energy obtained as a sum of electronic and thermal free energy of reagents and products involved in the reaction, respectively. Energies were calculated in *implicit* ACN (PCM model) using (U)CAM-B3LYP-D3(BJ)/def2-TZVP.

The Norrish Type II reaction mechanism of DETC with DBA was calculated using the approach based on transition state (TS) and Intrinsic Reaction Coordinate[63] (IRC), as implemented in Gaussian16. The energy profiles of forward and reverse directions for DETC in the first triplet state was explored. The transition state geometry was verified by vibrational analysis, and the final activation energy barrier was used in the Eyring equation[60] to determine the rate constant of the HAT reaction.

Table S23: Bond dissociation enthalpies (BDEs) for PETA and dibutylamine (DBA). Different types of possible PETA and DBA radicals (see Fig. S18) were investigated in order to detect which C-H bond is the most probable to be broken during the first step of the inter-HAT reaction with DETC. Values reported are in kcal mol^{-1}.

	**PETA**	**DBA**
R_1	**84.047**	**81.208**
R_2	85.253	88.198
R_3	99.576	87.622

R_4	92.642	91.642
R_5	103.263	-

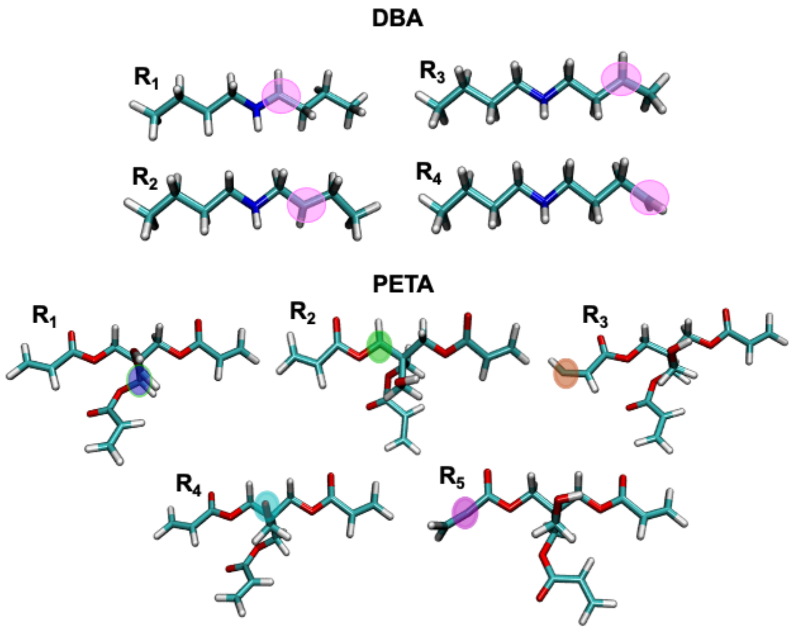

Fig. S18: The visualization of the possible types of dibutylamine (DBA) and PETA C-H bond dissociations toward radical formation. It was performed in order to detect which C-H bond is the most probable to be broken during the first step of the inter-HAT reaction with DETC.

From data reported in Table S23, we can see that the more probable radical to be formed on DBA and PETA upon reaction with DETC is radical "R_1" (for PETA see Fig. 6 and Tables S24-S5).
All computed BDEs energies for molecules in the ground state should be treated with the mean absolute error of around 2.4 kcal mol^{-1}, which was reported for BDEs calculated using CAM-B3LYP functional combined with def2-TZVP basis set in comparison to experiments[64,65]. It should be mentioned that the computed value reported in Table S23 for the BDE of R_1 in DBA (see Fig. S18) is in a very good agreement with experimentally reported BDEs for the same α(C−H) bond at ACN, i.e. of 88.9 kcal mol^{-1} [66] (here: 81.2 kcal mol^{-1}).

8.1 Reaction of DETC with amine co-initiator

The reaction between the first triplet excited state of DETC and the ground state of dibutylamine (DBA) was computed as the first (Fig. S19a). According to the radical formation mechanisms, the intermolecular H-atom transfer process involves the carbon-hydrogen bond of the alkyl chain of the amine and the carbonyl functionality of DETC. In the first step, the carbonyl group of DETC (with electronic structure in T_1) attacks the methyl group of DBA in the nearest positioning to the N atom, resulting in partial intermolecular H-atom transfer and later in the formation of transition state complex. Once this complex is formed, the complete

H-atom transfer to DETC results in the formation of the ketyl radical and α-aminoalkyl radical (Fig. S19b). The whole HAT reaction should be both thermodynamically favored and kinetically accessible, proceeding according to Norrish Type II reactions. Due to the steric hindrance, the ketyl radical formed on the photoinitiator is less active in initiating the PETA polymerization reaction than aminoalkyl radical on DBA (see Section 10). The energy barrier of such an intermolecular HAT reaction is 6.72 kcal mol^{-1} in *implicit* ACN for CAM-B3LYP allowing, in principle, for fast formation of reactive aminoalkyl and ketyl radicals, starting polymerization. The corresponding reaction rate using the activation energy barrier is $7.3x10^{7}$ s^{-1} (see Table S25).

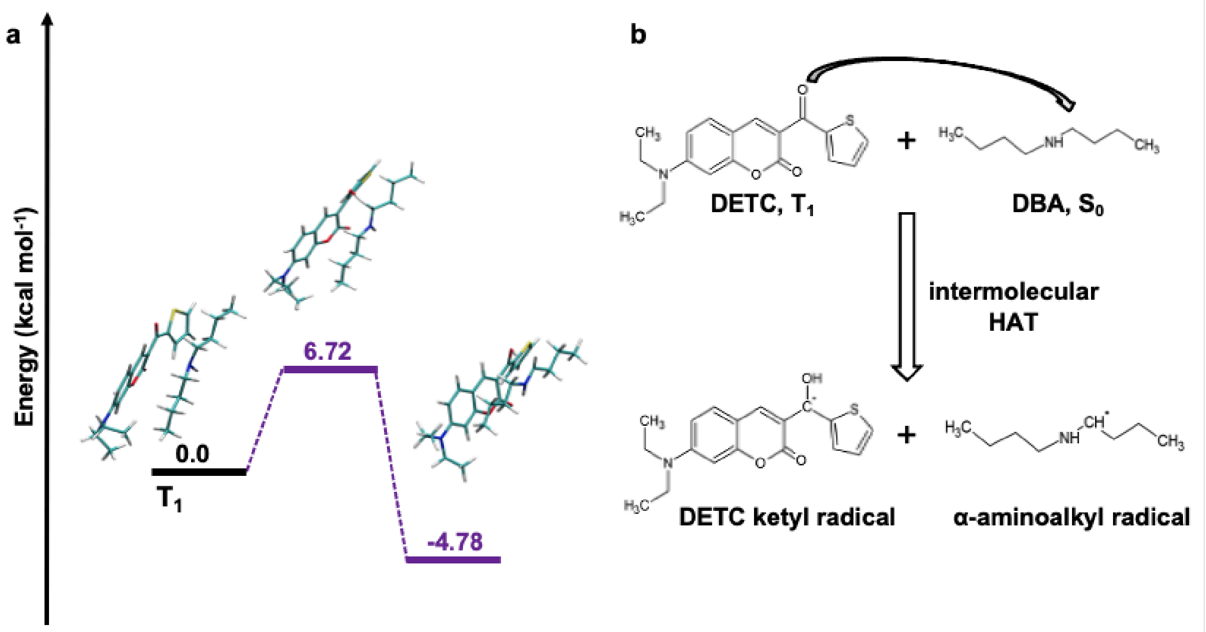

Fig. S19: The activation energy barrier for the formation of radicals according to the Norrish Type II mechanism calculated using CAM-B3LYP. a) The energy barrier of hydrogen atom transfer (HAT) reaction between the carbonyl moiety of DETC and the C15-H19 bond of dibutylamine (DBA) co-initiator forming a ketyl and an α-aminoalkyl radical, respectively. The reaction energy barrier was obtained performing Intrinsic Reaction Coordinate (IRC) calculation in *implicit* ACN starting from the T_1 state of DETC and S_0 state of DBA. b) The mechanism of HAT[67] reaction between DETC and the DBA co-initiator.

8.2 Reaction of DETC with PETA

Table S24: Gibbs free energy related to the Norrish type II radical formation reactions (inter-HAT mechanism) **between DETC, either in S_0 or in different excited states, with PETA.** Different types of possible PETA radicals were investigated (R_1-R_5) and reported in Fig. 6 in the main body in order to detect which C-H bond is the most probable to be broken in the first step of the reaction with the photoinitiator. The change in the Gibbs free energy of reaction was computed according to eq. 6 in the main body. All calculations were performed in *implicit* ACN with **(U)CAM-B3LYP-D3BJ/def2-TZVP** level of theory.

	PETA R_1	PETA R_2	PETA R_3	PETA R_4	PETA R_5

DETC S_0	**52.77**	**52.47**	62.42	66.40	70.75
DETC S_1	**-17.33**	**-17.62**	-7.68	-3.70	0.65
DETC T_1	**5.47**	**5.18**	15.11	19.10	23.45
DETC T_3	**-20.14**	**-20.43**	-10.49	-6.51	-2.156
DETC T_4	**-29.47**	**-29.76**	-19.82	-15.84	-11.49
DETC T_6	**-35.62**	**-35.92**	-25.98	-21.99	-17.64

Table S25: Comparison of Gibbs free energies and related to the Norrish type II radical formation reactions (inter-HAT mechanism) between DETC (either in S_0 or in different excited states) with dibutylamine (DBA) and PETA. The change in the Gibbs free energy of reaction was computed according to eq. 6 in the main body considering the formation of the R_1 type radical. E^a refers to the computation of the reaction energy barrier with the transition state and Intrinsic Reaction Coordinate (IRC). Rates were calculated employing the Eyring equation. All calculations were performed in *implicit* ACN with (U)CAM-B3LYP-D3BJ/def2-TZVP level of theory.

	**DBA**	**PETA**
**State**	**ΔG (kcal mol^{-1})**	**ΔG (kcal mol^{-1})**
S_0	48.19	52.77
S_1	-21.91	-17.33
T_1	0.44 (E^a: 6.72)*	5.47
T_2	-	-
T_3	-24.72	-20.14
T_4	-34.05	-29.47
T_5	-	-
T_6	-40.20	-35.62
T_7	-	-

*Rate for the reaction is $7.33x10^7$ s^{-1}

From the Gibbs free energies reported in Table S25, we can conclude that the inter-HAT reaction involving either PETA or DBA and DETC can not happen in absence of light (i.e. from S_0 state of DETC). However, values change if DETC reaches higher triplet excited states.

9. DETC radical formation mechanisms

Since DETC was shown to start polymerization without co-initiator (see Fig. 3), here we have investigated other hypothetical reaction mechanisms towards DETC radical formation from T_1 and from high triplet excited states that could lead to the formation of radicals. Three main reactions were modeled:

1) photoactivated H-abstraction in DETC,
2) DETC biradical formation,
3) DETC cleavage, i.e. photolysis.

To predict the affinity of the aminoalkyl chain of DETC to transfer H-atom inter- and intra-molecularly towards the formation of carbon-centered radical, with the same mechanism as previously reported for the reaction with DBA co-initiator (see Fig. S19), and to investigate the probability for photolysis involving different C-C and C-N bonds of DETC to occur spontaneously, Gibbs free energy of reactions and respective bond homolytic dissociation enthalpies, *i.e.* bond dissociation enthalpies (BDEs) as a function of the electronic state of DETC, were computed. Fig. S21 illustrates the key finding with regards to the possible formation of DETC radicals: data shows significant differences in the Gibbs free energy for different bonds and in different electronic states of DETC. A huge decrease of the energies is associated with DETC in higher triplet states. However, the process is not spontaneous in comparison to internal conversion to the lowest triplet state.

9.1. H-abstraction of DETC

Table S26: Bond dissociation enthalpies (BDEs) for different C-H bonds of DETC in the ground state (S_0), singlet (S_1), and triplet excited states. Bonds marked as $\mathbf{H_1}$-$\mathbf{H_5}$ are explained in Fig. S21a. All BDEs were calculated with **CAM-B3LYP-D3(BJ)/def2-TZVP** level of theory in implicit ACN. Results are reported in kcal mol^{-1}.

	**Bond dissociation energy (BDE)**				
	**H_1**	**H_2**	**H_3**	**H_4**	**H_5**
**S_0**	105.57	104.34	106.36	91.34	81.97
**S_1**	35.21	33.98	36.00	20.98	11.62
**T_1**	57.59	56.36	58.38	43.36	33.99
**T_3**	32.40	31.18	33.20	18.17	8.81
**T_4**	22.54	21.32	23.33	8.31	-1.06

T6	15.71	14.49	16.51	1.49	-7.88

Table S27: Gibbs free energy of reactions related to the cleavage of bond H_5 of DETC in the ground state (S_0), singlet (S_1), and triplet excited state structures. All ΔG were calculated with CAM-B3LYP-D3(BJ)/def2-TZVP level of theory in implicit ACN. Results are reported in kcal mol^{-1}.

Gibbs free energy of reaction (ΔG)	
**State**	**HAT in DETC (H_5)**
S_0	89.89
S_1	19.80
T_1	42.15
T_3	16.99
T_4	7.66
T_6	1.50

Energies calculated indicate significant impact of photoactivation of DETC on the "movable" affinity of H atom towards radical formation. The H-atom abstraction process for "H5" is associated with the lowest Gibbs free energy, which agrees with the reactivity induction in molecules containing heteroatoms. It is known that polar functionalities, as *e.g.* N-atoms, may influence the reactivity of neighboring C-H bonds through the polarity match and hyperconjugation effect enabling higher tendency of bond cleavage by the H-abstractor and stabilization of the radical intermediate for further HAT[67]. This process can not occur from the T_1 state of DETC due to the high Gibbs free energies and BDE related to the abstraction of "H5": 33.99 kcal mol^{-1}.

Data reported are in agreement with previously reported works[64,66,68] and BDEs calculated for primary and secondary C-H bonds in aliphatic hydrocarbons (of *ca.* 100 kcal mol^{-1}). Moreover, it is similar to the BDE necessary to cleave the C-H bond of DBA (*i.e.* 88.9 kcal mol^{-1} in ACN) for intermolecular HAT, discussed in Section 8. All computed BDEs energies for molecules in the ground state should be treated with the mean absolute error of around 2.4 kcal mol^{-1}, which was reported for BDEs calculated using CAM-B3LYP combined with def2-TZVP basis set in comparison to experiments[64,65].

9.2. Photolysis of DETC

We have also calculated the Gibbs free energies and BDEs of other bonds that may be considered of forming radicals upon cleavage in photoexcited DETC, i.e. photolysis. Here, we investigated the probability of possible C-C and C-N bonds to be broken (Fig. S21c, Table S28, S29). In comparison to H-abstraction, we have found lower Gibbs free energies for α-scission in T_4 state towards the formation of carbon centered radicals, e.g.. the value for C8-C11 bond is -0.52 kcal mol^{-1}, for C11-C17 is -2.21 kcal mol^{-1} for C15-C16 is -19.17 kcal mol^{-1} (Table S29) vs 7.66 kcal mol^{-1} for H-abstraction (Table S27). However, this is still around 10 kcal mol^{-1} less spontaneous than the Gibbs free energy of the reaction between DETC and PETA or DBA (Table S25). Only C15-C16 and N14-C15 bonds show a tendency towards breaking even in the T_3 state. They are also less spontaneous than the reaction with PETA, however the process is an example of a unimolecular reaction in comparison to the bimolecular reaction with PETA (or DBA). To estimate, if this pathway is capable of competing with IC processes to the lowest triplet state, experimental validation would be necessary.

Table S28: Bond dissociation enthalpies (BDEs) for different bonds of DETC in the ground state (S_0), singlet (S_1), and triplet excited state structures. Bonds are depicted in Fig. S21c. Atom numbering is reported in Fig. S1. All BDEs were calculated with CAM-B3LYP-D3(BJ)/def2-TZVP level of theory in implicit ACN. Results are reported in kcal mol^{-1}.

	**Bond dissociation enthalpy (BDEs)**				
	**C11-C17**	**N14-C6**	**C8-C11**	**C15-C16**	**N14-C15**
**S_0**	95.22	99.23	95.72	76.17	70.06
**S_1**	24.86	28.88	25.36	5.81	-0.30
**T_1**	47.24	51.25	47.73	28.19	22.08
**T_3**	22.06	26.07	22.55	3.01	-3.10
**T_4**	22.19	16.20	12.68	-6.86	-12.97
**T_6**	5.37	9.38	5.86	-13.69	-19.80

Table S29: Gibbs free energies for the bond scission in the ground state (S_0), singlet (S_1) and triplet excited state structures. ΔG were calculated with CAM-B3LYP-D3(BJ)/def2-TZVP level of theory in implicit ACN. Results are reported in kcal mol^{-1}.

	Gibbs free energy of reaction (ΔG)				
	**C11-C17**	**N14-C6**	**C8-C11**	**C15-C16**	**N14-C15**
S_0	80.03	85.34	81.72	63.07	55.28
S_1	9.93	15.24	11.62	-7.09	-14.82
T_1	32.18	37.59	33.97	15.32	7.53
T_3	7.13	12.43	8.82	-9.84	-17.63
T_4	-2.21	3.10	-0.52	-19.17	-26.96
T_6	-8.35	-3.06	-6.67	-25.39	-33.11

9.3. Biradicals formation of DETC

The intramolecular HAT has been previously suggested for other chromophore molecules[62,69,70], however, the realization of distance criteria for such HAT was never confirmed. If this process is possible, DETC could form biradicals after the activated H-abstraction (see Section 9.1) and aminoalkyl radical would be the one that can initiate polymerization due to the higher steric hindrance of the ketyl radical.

We have studied biradicals of DETC via enthalpy and Gibbs free energies of hydrogen abstraction and attachment using global minima structures of DETC at different potential energy surfaces. Ten biradicals divided into two main groups, marked as “a” and “b”, differing by the carbonyl group considered, *i.e.* C11-O12 for biradicals **1-5Bi-a** and C9-O13 for biradicals **1-5Bi-b** are reported in Fig. S20. For DETC biradicals, $products$ in eq. 6 in the main paper referred to the biradicals formed (**1Bi-a** to **5Bi-a** and **1Bi-b** to **5Bi-b**) and $reagents$ to each optimized state considered (ground state, singlet or triplet).

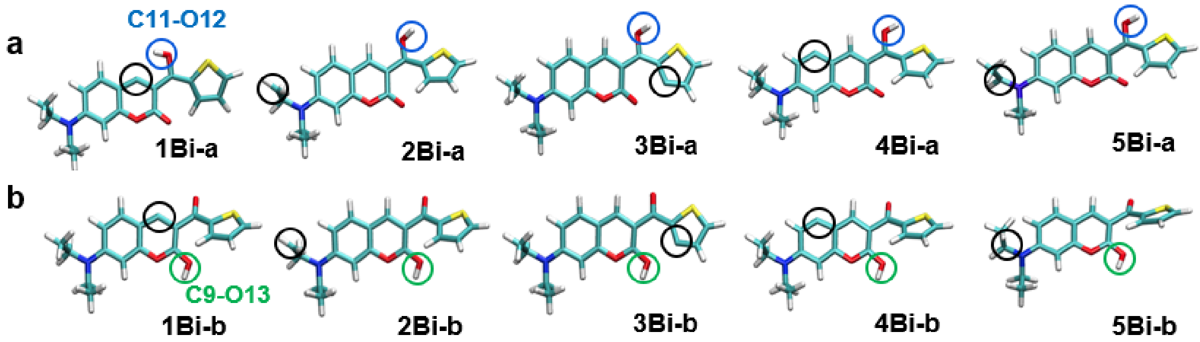

Fig. S20: **a**) DETC biradicals calculated: **1Bi-a** to **5Bi-a** formed via ketyl radical on C11 (C11-O12 carbonyl bond, marked in blue) and aminoalkyl radical on C-atom in H_1-H_5 positions (Fig. S21) (**c**) with the corresponding Gibbs free energies. **b**) Biradicals **1Bi-b** to **5Bi-b** formed via C9-O13 carbonyl (marked in green) and aminoalkyl moiety (marked in black) (**d**) with the corresponding Gibbs free energies.

Even if expected biradical formation could happen mainly from the T_4 triplet excited state, all electronic states of DETC analyzed in this report were considered for comparison. Results obtained demonstrate that the formation of biradicals should be spontaneous even from triplet states higher than T_3. The formation of biradical **5Bi-a** is associated with the lowest Gibbs free energies, i.e. in the range -31 to -37 kcal mol^{-1}, related to high triplet excited states (see Table S30). However, **considering the BDE energies and the Gibbs free energies of C-H bond breaking (Section 9.1) that are still high, hindering spontaneity of H-atom abstraction (see Table S27) in comparison to internal conversion to T_1 state ($k_{Tx\text{-}T1}$ of ca. 10^{11}, see Table 1 and S19b), we assume the formation of these biradicals is inaccessible.** The presence of such a long range intra-HAT has been never confirmed experimentally.

Table S30: Bond dissociation enthalpies (BDEs) of DETC biradicals formation from the ground state (S_0), singlet (S_1), and triplet excited state structures. Formation of biradicals, considering ketyl radical generation on **C11-O12** carbonyl bond, labeled from **1Bi-a** to **5Bi-a** is depicted in Fig. S20. Energies obtained with CAM-B3LYP-D3(BJ)/def2-TZVP level of theory in *implicit* **ACN**. Results are reported in kcal mol^{-1}.

	**Bond dissociation energies (BDEs)**				
	**1Bi-a**	**2Bi-a**	**3Bi-a**	**4Bi-a**	**5Bi-a**
**S_0**	69.95	60.22	73.38	72.70	**52.28**
**S_1**	-0.40	-10.13	3.02	2.35	**-18.08**
**T_1**	21.97	12.24	25.39	24.72	**4.30**
**T_3**	-3.21	-12.94	0.21	-0.46	**-20.88**

**T_4**	-1308	-22.81	-9.65	-10.33	**-30.75**
**T_6**	-19.90	-29.63	-16.47	-17.15	**-35.58**

Table S31: Gibbs free energy of DETC biradicals formation (ΔG) from the ground state (S_0), singlet (S_1), and triplet excited state structures. Formation of biradicals, considering alkoxy radical generation on **C11-O12** carbonyl bond, labeled from **1Bi-a** to **5Bi-a** and energies are depicted in Fig. S20. Energies obtained with CAM-B3LYP-D3(BJ)/def2-TZVP level of theory in *implicit* **ACN**. Results are reported in kcal mol^{-1}.

	**Gibbs free energy of reaction (ΔG)**				
	**1Bi-a**	**2Bi-a**	**3Bi-a**	**4Bi-a**	**5Bi-a**
**S_0**	68.89	58.75	72.41	71.78	**50.77**
**S_1**	-1.20	-11.35	2.32	1.69	**-19.32**
**T_1**	21.59	11.45	25.11	24.48	**3.47**
**T_3**	-4.01	-14.15	-0.49	-1.12	**-22.13**
**T_4**	-13.34	-23.49	-9.82	-10.45	**-31.46**
**T_6**	-19.50	-29.64	-15.98	-16.61	**-37.62**

The intermolecular HAT between DETC molecules has not been investigated due to the low concentration of the photoinitiator (0.25 and 0.50 wt%[59,71–73]), limited by its low solubility into the monomer, during the printing condition.

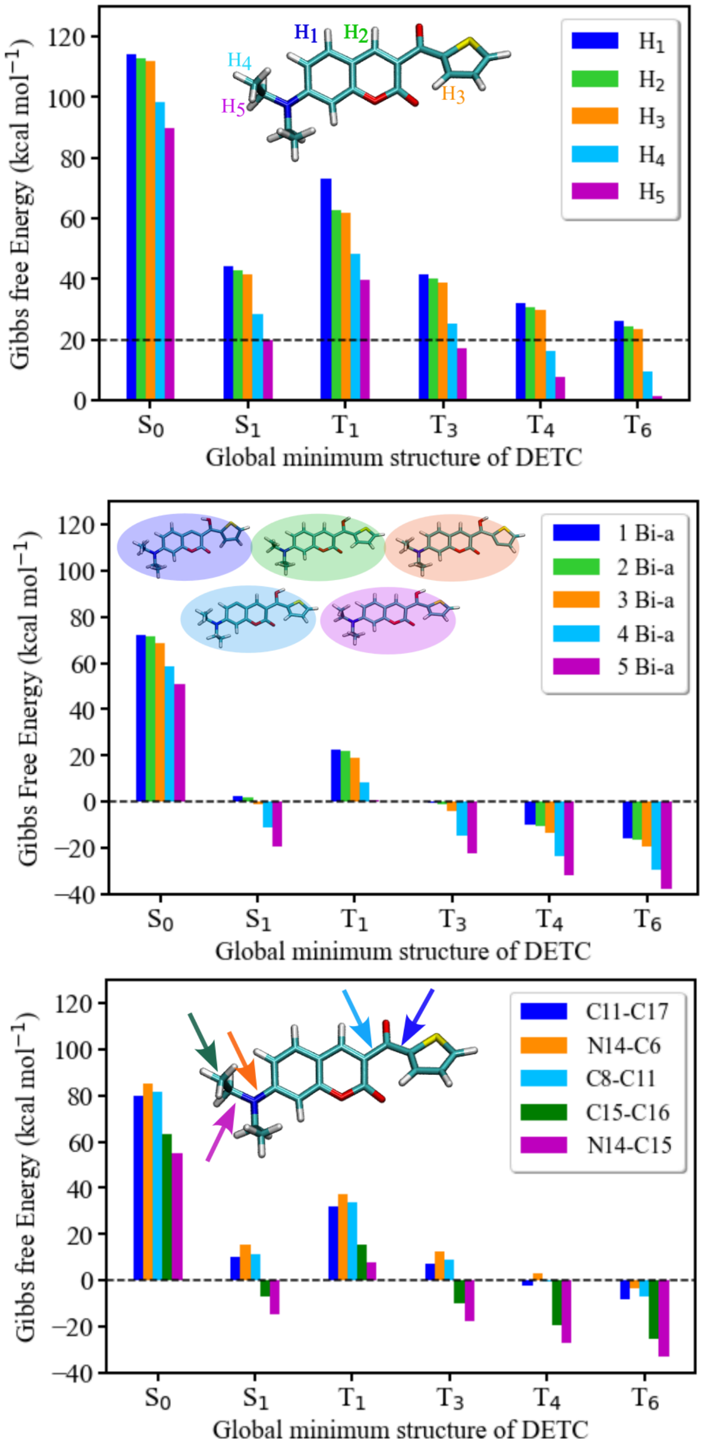

Fig. S21: Gibbs free energy for different hypothetical DETC radical formations: a) C-H bond breaking (H_1-H_5), b) biradical formation c) photolysis as a function of the DETC electronic state. Energies were computed employing (U)CAM-B3LYP-D3(BJ)/def2-TZVP approach in *implicit* ACN.

10. Polymerization mechanism

In general, during the free-radical polymerization (FRP) of PETA, chain-reaction is allowed due to the conversion of the α,β-unsaturated double bond of PETA monomer into a single C-C bond[74] (Fig. S22). This reaction can be triggered by different types of radicals, which are experimentally unknown. To estimate the spontaneity of FRP of PETA by hypothetically considered radicals, explained in Section 8 and 9, Gibbs free energy of these reactions were calculated. Specifically, we have considered:

- α-aminoalkyl radical of DETC formed either via a) intramolecular HAT, i.e. DETC biradical (**step 1a** in Fig. S22) or b) intermolecular HAT (**step 1b** in Fig. S22),
- DETC ketyl radical (**step 1c** in Fig. S22), as well as the
- alkyl radical of PETA (**step 1d** in Fig. S22) generated by intermolecular HAT with DETC in (probably) high triplet states,
- α-aminoalkyl radical of DBA (**step 1e** in Fig. S22) generated by intermolecular HAT with DETC in (probably) high triplet states,
- alkyl radicals (**step 1f** and **1g** in Fig. S22) formed on DETC fragments upon photolysis of bond C8-C11. We have also calculated radicals formed upon the cleavage of the N14-C15 bond.

Upon initiation of the radical formed (**step 2a-g**), it should react with the next PETA monomer to form another alkyl radical, which subsequently forms a radical chain (**step 3**) or crosslinks until the termination step occurs. Important to mention that the propagation step might be characterized by many transfer processes since polymerization mechanisms are usually complex and characterized by many different processes e.g disproportion or radical coupling reactions that can terminate the growing polymer chain.

In Fig. S22, we report hypothetical radical polymerization mechanisms during the FRP initiation**.** In Section 8 and 9 of SI we reported the computed Gibbs free energies related to each of the radical generation reactions mentioned above. In Table S32, we summarized the key findings of the FRP of PETA monomers in the case of the DETC usage. Data reported show that the reaction between the α-aminoalkyl DETC radical (**step 1a, 1b**) or alkyl DETC radical (**step 1f, 1g**) with PETA may be spontaneous, therefore if these radicals could be formed, they could possibly initiate FRP. However, in Section 9 we have explained that the formation of most of these radicals is hindered due to faster internal conversion to T_1. In addition, we show only the values of the Gibbs free energy calculated with implicit solvation using harmonic approximation and without the consideration of non-equilibrium processes, subtle dynamical changes, explicit entropic contributions and kinetics to make more definite conclusions. Moreover, the experimental validation would be highly beneficial.

We also see that the radicals generated upon the photolysis of the N14-C15 bond show more negative Gibbs free energies (see Table S29) than other bond cleavage possibilities. Here, the radical generated on the C atom should be more reactive than the N-centered radical (see Table S32). If it is formed, it could spontaneously react with PETA similarly to the reaction of the α-aminoalkyl radical of DBA with PETA. Still, the initiation reaction between two PETA molecules should be more spontaneous. Ketyl radical of DETC does not allow spontaneous FRP, so it is less reactive than all the others, which was also reported experimentally[75,76].

FRP is spontaneous, when the α-aminoalkyl radical generated on DBA and the alkyl radical of PETA (**step 1d, 1e**) are present. The formation of these radicals was shown to be

permitted due to the DETC excitation to higher triplet states (see Table S25). Since the nonlinearity of 3D printing with DETC+DBA+PETA has not changed from ~3 to ~2, we tend to believe that the formation of these radicals is hindered in the presence of DETC in T_1, therefore, TA to higher triplets states is necessary to excite DETC towards DBA and PETA radical activation.

Table S32: Gibbs free energies and the respective entropy factors of hypothetical radical generation mechanisms that should lead to the conversion of the α,β-unsaturated double C=C bond of PETA into a single C-C bond (**step 1a-g**). All calculations were performed in Gaussian16 at (U)CAM-B3LYP-D3(BJ)/def2-TZVP level of theory.

**Step**	**Components**	**ΔG (kcal mol^{-1})**	**ΔS (kcalmol^{-1}K^{-1})**
step 1a	biradical DETC + PETA	-5.458	-0.064
step 1b	aminoalkyl radical DETC + PETA	-11.998	-0.044
step 1c	ketyl radical DETC + PETA	-0.373	0.089
step 1d	alkyl radical PETA + PETA	-25.105	-0.057
step 1e	aminoalkyl radical DBA + PETA	-14.898	-0.075
step 1f	photolysis C radical (C11) + PETA	-41.418	0.114
step 1g	photolysis C radical (C8) + PETA	-20.283	-0.041
step 1h*	photolysis C radical (C15) + PETA	-15.331	-0.043
step 1i*	photolysis N radical (N14) + PETA	-2.033	-0.043

*not reported in Fig. S22 and related to the cleavage of bond N14-C15

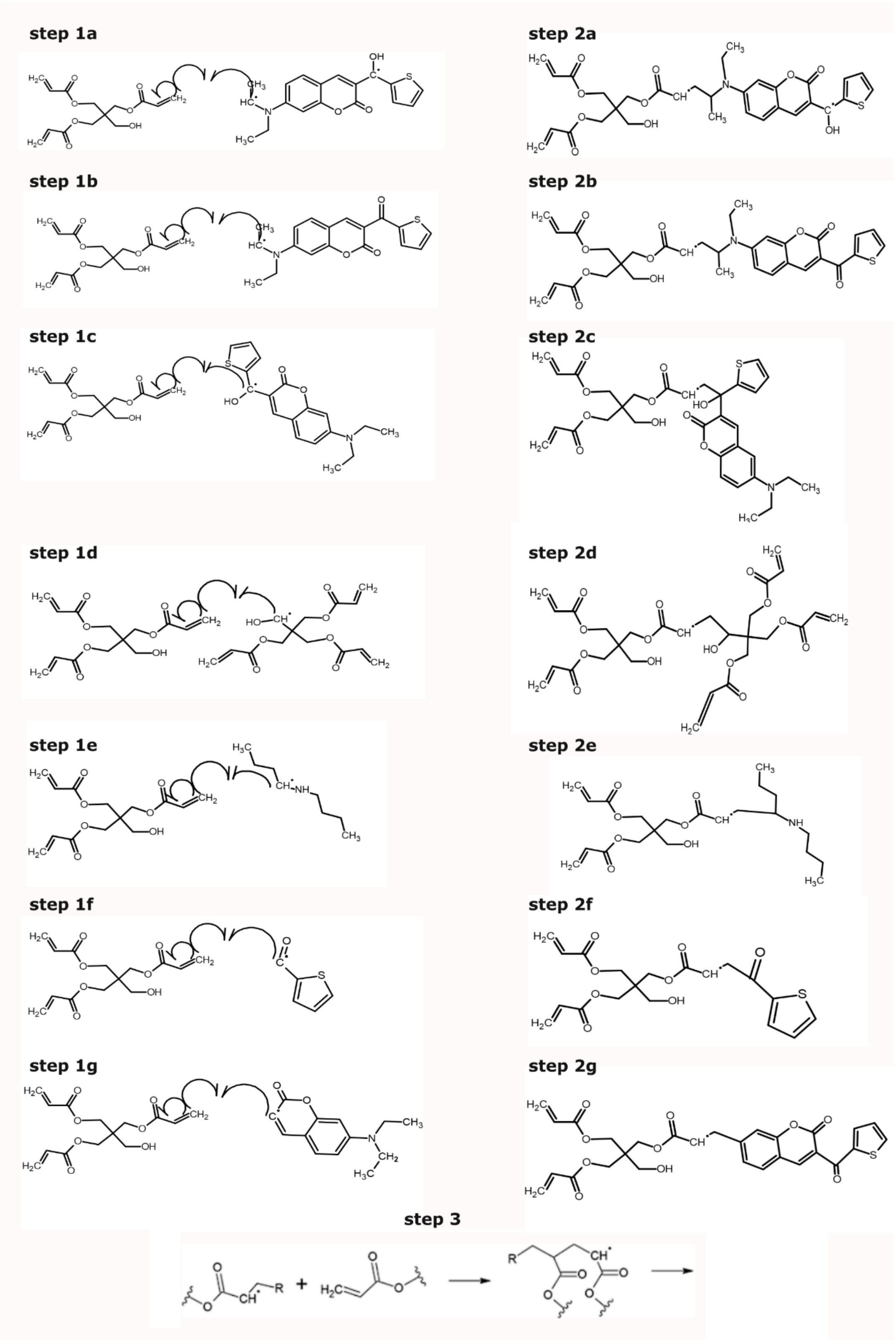

Fig. S22: Hypothetical photopolymerization reaction mechanisms that could activate PETA monomer through the free-radical polymerization (FRP) that should lead to the conversion of the α,β-unsaturated double C=C bond of PETA into a single C-C bond (**step 1a-g**). Propagation radical that could be formed (**step 2a-g**) with the general polymerization propagation scheme (**step 3**).

References

1. Frisch, M. J. *et al. Gaussian 16*. (Gaussian, Inc. Wallingford, CT, 2016).
2. Niu, Y. *et al.* MOlecular MAterials Property Prediction Package (MOMAP) 1.0: a software package for predicting the luminescent properties and mobility of organic functional materials. *Molecular Physics* **116**, 1078–1090 (2018).
3. Peng, Q., Yi, Y., Shuai, Z. & Shao, J. Toward Quantitative Prediction of Molecular Fluorescence Quantum Efficiency: Role of Duschinsky Rotation. *J. Am. Chem. Soc.* **129**, 9333–9339 (2007).
4. Peng, Q., Niu, Y., Shi, Q., Gao, X. & Shuai, Z. Correlation Function Formalism for Triplet Excited State Decay: Combined Spin–Orbit and Nonadiabatic Couplings. *J. Chem. Theory Comput.* **9**, 1132–1143 (2013).
5. Niu, Y., Peng, Q. & Shuai, Z. Promoting-mode free formalism for excited state radiationless decay process with Duschinsky rotation effect. *Sci. China Ser. B-Chem.* **51**, 1153–1158 (2008).
6. Shuai, Z. & Peng, Q. Organic light-emitting diodes: theoretical understanding of highly efficient materials and development of computational methodology. *National Science Review* **4**, 224–239 (2017).
7. Shuai, Z. & Peng, Q. Excited states structure and processes: Understanding organic light-emitting diodes at the molecular level. *Physics Reports* **537**, 123–156 (2014).
8. te Velde, G. *et al.* Chemistry with ADF. *J. Comput. Chem.* **22**, 931–967 (2001).
9. Aidas, K. *et al.* The Dalton quantum chemistry program system: The Dalton program. *WIREs Comput Mol Sci* **4**, 269–284 (2014).
10. Tian, G., Duan, S., Hua, W. & Luo, Y. DynaVib Version 1.0. *Royal Institute of Technology, Sweden* (2012).
11. Pan, Y. *et al.* On the spectral profile change in the Q band absorption spectra of metalloporphyrins (Mg, Zn, and Pd): A first-principles study. *J. Chem. Phys.* **150**, 164308 (2019).

12. TURBOMOLE V7.4 2019, a development of University of Karlsruhe and Forschungszentrum Karlsruhe GmbH, 1989-2007, TURBOMOLE GmbH, since 2007; available from \ \tt http://www.turbomole.com.

13. Scalmani, G. & Frisch, M. J. Continuous surface charge polarizable continuum models of solvation. I. General formalism. *J. Chem. Phys.* **132**, 114110 (2010).

14. Yanai, T., Tew, D. P. & Handy, N. C. A new hybrid exchange–correlation functional using the Coulomb-attenuating method (CAM-B3LYP). *Chemical Physics Letters* **393**, 51–57 (2004).

15. Krause, K. & Klopper, W. Implementation of the Bethe−Salpeter equation in the TURBOMOLE program. *J. Comput. Chem.* **38**, 383–388 (2017).

16. van Setten, M. J., Weigend, F. & Evers, F. The *GW* -Method for Quantum Chemistry Applications: Theory and Implementation. *J. Chem. Theory Comput.* **9**, 232–246 (2013).

17. Santoro, F., Improta, R., Lami, A., Bloino, J. & Barone, V. Effective method to compute Franck-Condon integrals for optical spectra of large molecules in solution. *The Journal of Chemical Physics* **126**, 084509 (2007).

18. Santoro, F., Lami, A., Improta, R., Bloino, J. & Barone, V. Effective method for the computation of optical spectra of large molecules at finite temperature including the Duschinsky and Herzberg–Teller effect: The Qx band of porphyrin as a case study. *The Journal of Chemical Physics* **128**, 224311 (2008).

19. Herzberg, G. & Teller, E. Schwingungsstruktur der Elektronenübergänge bei mehratomigen Molekülen. *Zeitschrift für Physikalische Chemie* **21B**, 410–446 (1933).

20. Avila Ferrer, F. J., Cerezo, J., Stendardo, E., Improta, R. & Santoro, F. Insights for an Accurate Comparison of Computational Data to Experimental Absorption and Emission Spectra: Beyond the Vertical Transition Approximation. *J. Chem. Theory Comput.* **9**, 2072–2082 (2013).

21. Jacquemin, D. & Escudero, D. The short device lifetimes of blue PhOLEDs: insights into the photostability of blue Ir(III) complexes. *Chem. Sci.* **8**, 7844–7850 (2017).

22. Jacquemin, D., Planchat, A., Adamo, C. & Mennucci, B. TD-DFT Assessment of

Functionals for Optical 0–0 Transitions in Solvated Dyes. *J. Chem. Theory Comput.* **8**, 2359–2372 (2012).

23. Caricato, M. *et al.* Formation and relaxation of excited states in solution: A new time dependent polarizable continuum model based on time dependent density functional theory. *The Journal of Chemical Physics* **124**, 124520 (2006).

24. Improta, R., Barone, V., Scalmani, G. & Frisch, M. J. A state-specific polarizable continuum model time dependent density functional theory method for excited state calculations in solution. *The Journal of Chemical Physics* **125**, 054103 (2006).

25. Fang, C., Oruganti, B. & Durbeej, B. How Method-Dependent Are Calculated Differences between Vertical, Adiabatic, and 0–0 Excitation Energies? *J. Phys. Chem. A* **118**, 4157–4171 (2014).

26. Winter, N. O. C., Graf, N. K., Leutwyler, S. & Hättig, C. Benchmarks for 0–0 transitions of aromatic organic molecules: DFT/B3LYP, ADC(2), CC2, SOS-CC2 and SCS-CC2 compared to high-resolution gas-phase data. *Phys. Chem. Chem. Phys.* **15**, 6623–6630 (2013).

27. Autschbach, J. & Srebro, M. Delocalization Error and “Functional Tuning” in Kohn–Sham Calculations of Molecular Properties. *Acc. Chem. Res.* **47**, 2592–2602 (2014).

28. Beerepoot, M. T. P., Friese, D. H., List, N. H., Kongsted, J. & Ruud, K. Benchmarking two-photon absorption cross sections: performance of CC2 and CAM-B3LYP. *Phys. Chem. Chem. Phys.* **17**, 19306–19314 (2015).

29. Jacquemin, D. *et al.* On the Performances of the M06 Family of Density Functionals for Electronic Excitation Energies. *J. Chem. Theory Comput.* **6**, 2071–2085 (2010).

30. Adamo, C. & Jacquemin, D. The calculations of excited-state properties with Time-Dependent Density Functional Theory. *Chem. Soc. Rev.* **42**, 845–856 (2013).

31. Batra, K., Zahn, S. & Heine, T. Benchmark of Simplified Time-Dependent Density Functional Theory for UV–Vis Spectral Properties of Porphyrinoids. *Adv. Theory Simul.* **3**, 1900192 (2020).

32. Holzer, C. & Klopper, W. Ionized, electron-attached, and excited states of molecular systems with spin–orbit coupling: Two-component *GW* and Bethe–Salpeter implementations. *J. Chem. Phys.* **150**, 204116 (2019).

33. Cortés-Mejía, R., Höfener, S. & Klopper, W. Effects of rotational conformation on electronic properties of 4,4′-bis(carbazol-9-yl)biphenyl (CBP): the single-molecule picture and beyond. *Molecular Physics* **119**, e1876936 (2021).

34. Rohlfing, M. & Louie, S. G. Excitonic Effects and the Optical Absorption Spectrum of Hydrogenated Si Clusters. *Phys. Rev. Lett.* **80**, 3320–3323 (1998).

35. Blase, X. & Attaccalite, C. Charge-transfer excitations in molecular donor-acceptor complexes within the many-body Bethe-Salpeter approach. *Appl. Phys. Lett.* **99**, 171909 (2011).

36. Weigend, F. Accurate Coulomb-fitting basis sets for H to Rn. *Phys. Chem. Chem. Phys.* **8**, 1057 (2006).

37. Eichkorn, K., Treutler, O., Öhm, H., Häser, M. & Ahlrichs, R. Auxiliary basis sets to approximate Coulomb potentials (Chem. Phys. Letters 240 (1995) 283-290). *Chemical Physics Letters* **242**, 652–660 (1995).

38. Sierka, M., Hogekamp, A. & Ahlrichs, R. Fast evaluation of the Coulomb potential for electron densities using multipole accelerated resolution of identity approximation. *The Journal of Chemical Physics* **118**, 9136–9148 (2003).

39. Ahlrichs, R. Efficient evaluation of three-center two-electron integrals over Gaussian functions. *Phys. Chem. Chem. Phys.* **6**, 5119 (2004).

40. Lu, T. & Chen, F. Multiwfn: A multifunctional wavefunction analyzer. *J. Comput. Chem.* **33**, 580–592 (2012).

41. Liu, Z., Lu, T. & Chen, Q. An sp-hybridized all-carboatomic ring, cyclo[18]carbon: Electronic structure, electronic spectrum, and optical nonlinearity. *Carbon* **165**, 461–467 (2020).

42. Le Bahers, T., Adamo, C. & Ciofini, I. A Qualitative Index of Spatial Extent in Charge-Transfer Excitations. *J. Chem. Theory Comput.* **7**, 2498–2506 (2011).

43. Hättig, C., Christiansen, O. & Jo/rgensen, P. Multiphoton transition moments and absorption cross sections in coupled cluster response theory employing variational transition moment functionals. *The Journal of Chemical Physics* **108**, 8331–8354 (1998).

44. Cronstrand, P., Norman, P., Luo, Y. & Ågren, H. Few-states models for three-photon absorption. *The Journal of Chemical Physics* **121**, 2020–2029 (2004).

45. Zhang, Y., Su, Y., Zhao, Y., Wang, Z. & Wang, C. Two-Photon 3D Printing in Metal–Organic Framework Single Crystals. *Small* **18**, 2200514 (2022).

46. Wiggins, P., Williams, J. A. G. & Tozer, D. J. Excited state surfaces in density functional theory: A new twist on an old problem. *The Journal of Chemical Physics* **131**, 091101 (2009).

47. Cao, C. *et al.* A twisted-intramolecular-charge-transfer (TICT) based ratiometric fluorescent thermometer with a mega-Stokes shift and a positive temperature coefficient. *Chem. Commun.* **50**, 15811–15814 (2014).

48. Suellen, C., Freitas, R. G., Loos, P.-F. & Jacquemin, D. Cross-Comparisons between Experiment, TD-DFT, CC, and ADC for Transition Energies. *J. Chem. Theory Comput.* **15**, 4581–4590 (2019).

49. Tajti, A., Tulipán, L. & Szalay, P. G. Accuracy of Spin-Component Scaled ADC(2) Excitation Energies and Potential Energy Surfaces. *J. Chem. Theory Comput.* **16**, 468–474 (2020).

50. Shuai, Z., Wang, D., Peng, Q. & Geng, H. Computational Evaluation of Optoelectronic Properties for Organic/Carbon Materials. *Acc. Chem. Res.* **47**, 3301–3309 (2014).

51. Mebel, A. M., Hayashi, M., Liang, K. K. & Lin, S. H. Ab Initio Calculations of Vibronic Spectra and Dynamics for Small Polyatomic Molecules: Role of Duschinsky Effect. *J. Phys. Chem. A* **103**, 10674–10690 (1999).

52. Li, F. *et al.* Thermally activated delayed fluorescence emitters with dual conformations for white organic light-emitting diodes: mechanism and molecular design. *Phys. Chem. Chem. Phys.* **22**, 1313–1323 (2020).

53. Lenthe, E. van, Baerends, E. J. & Snijders, J. G. Relativistic regular two-component Hamiltonians. *The Journal of Chemical Physics* **99**, 4597–4610 (1993).

54. van Lenthe, E., Baerends, E. J. & Snijders, J. G. Relativistic total energy using regular approximations. *The Journal of Chemical Physics* **101**, 9783–9792 (1994).

55. van Lenthe, E., Ehlers, A. & Baerends, E.-J. Geometry optimizations in the zero order regular approximation for relativistic effects. *The Journal of Chemical Physics* **110**, 8943–8953 (1999).

56. Klamt, A. & Schüürmann, G. COSMO: a new approach to dielectric screening in solvents with explicit expressions for the screening energy and its gradient. *J. Chem. Soc., Perkin Trans. 2* 799–805 (1993) doi:10.1039/P29930000799.

57. Jana, B., Inamdar, S. R. & H.M., S. K. Effect of quencher and temperature on fluorescence intensity of laser dyes: DETC and C504T. *Spectrochimica Acta Part A: Molecular and Biomolecular Spectroscopy* **170**, 124–130 (2017).

58. Young, D. *Computational Chemistry: A Practical Guide for Applying Techniques to Real World Problems*. (John Wiley & Sons, 2004).

59. Kiefer, P. *et al.* Sensitive Photoresists for Rapid Multiphoton 3D Laser Micro- and Nanoprinting. *Advanced Optical Materials* **8**, 2000895 (2020).

60. Johnson, J. E., Chen, Y. & Xu, X. Model for polymerization and self-deactivation in two-photon nanolithography. *Opt. Express* **30**, 26824 (2022).

61. Liaros, N. *et al.* Elucidating complex triplet-state dynamics in the model system isopropylthioxanthone. *iScience* **25**, 103600 (2022).

62. Ackermann, T. Thermochemical kinetics. Methods for the estimation of thermochemical data and rate parameters. Von S. W. Benson. John Wiley & Sons, Inc., New York 1976. 2. Aufl., XI, 320 S., 15 Abb., geb. $ 28.50. *Angew. Chem.* **89**, 921–921 (1977).

63. Gonzalez, Carlos. & Schlegel, H. Bernhard. Reaction path following in mass-weighted internal coordinates. *J. Phys. Chem.* **94**, 5523–5527 (1990).

64. St. John, P. C., Guan, Y., Kim, Y., Kim, S. & Paton, R. S. Prediction of organic

homolytic bond dissociation enthalpies at near chemical accuracy with sub-second computational cost. *Nat Commun* **11**, 2328 (2020).

65. Yu, L.-J., Dale, S. G., Chan, B. & Karton, A. Benchmark study of DFT and composite methods for bond dissociation energies in argon compounds. *Chemical Physics* **531**, 110676 (2020).

66. Lalevée, J., Allonas, X. & Fouassier, J.-P. N−H and α(C−H) Bond Dissociation Enthalpies of Aliphatic Amines. *J. Am. Chem. Soc.* **124**, 9613–9621 (2002).

67. Capaldo, L., Ravelli, D. & Fagnoni, M. Direct Photocatalyzed Hydrogen Atom Transfer (HAT) for Aliphatic C–H Bonds Elaboration. *Chem. Rev.* **122**, 1875–1924 (2022).

68. Gerö, L. Bond Energies of Hydrocarbons. *The Journal of Chemical Physics* **16**, 1011–1013 (1948).

69. Hioe, J. & Zipse, H. Radical stability and its role in synthesis and catalysis. *Org. Biomol. Chem.* **8**, 3609 (2010).

70. Ding, G. *et al.* Conjugated dyes carrying N, N-dialkylamino and ketone groups: One-component visible light Norrish type II photoinitiators. *Dyes and Pigments* **137**, 456–467 (2017).

71. Fischer, J. *et al.* Exploring the Mechanisms in STED-Enhanced Direct Laser Writing. *Advanced Optical Materials* **3**, 221–232 (2015).

72. Yang, L. *et al.* On the Schwarzschild Effect in 3D Two-Photon Laser Lithography. *Advanced Optical Materials* **7**, 1901040 (2019).

73. Somers, P. *et al.* Photo-activated polymerization inhibition process in photoinitiator systems for high-throughput 3D nanoprinting. *Nanophotonics* **0**, (2023).

74. Leggesse, E. G., Tong, W.-R., Nachimuthu, S., Chen, T.-Y. & Jiang, J.-C. Theoretical study on photochemistry of Irgacure 907. *Journal of Photochemistry and Photobiology A: Chemistry* **347**, 78–85 (2017).

75. Fouassier, J.-P., Ruhlmann, D., Takimoto, Y., Harada, M. & Kawabata, M. New three-component initiation systems in UV curing: A time-resolved laser-spectroscopy investigation. *J. Polym. Sci. A Polym. Chem.* **31**, 2245–2248 (1993).

76. Dadashi-Silab, S., Doran, S. & Yagci, Y. Photoinduced Electron Transfer Reactions for Macromolecular Syntheses. *Chem. Rev.* **116**, 10212–10275 (2016).
